# Therapeutic Potential of Metabotropic Glutamate Receptor Modulators

**DOI:** 10.2174/157015912799362805

**Published:** 2012-03

**Authors:** N Hovelsø, F Sotty, L.P Montezinho, P.S Pinheiro, K.F Herrik, A Mørk

**Affiliations:** 1 Department of Neurophysiology, H. Lundbeck A/S, Ottiliavej 9, 2500 Copenhagen-Valby, Denmark; 2 Department of Neuroscience and Pharmacology, Faculty of Health Sciences, University of Copenhagen, 2200 Copenhagen N, Denmark

**Keywords:** Addiction, alzheimer’s disease, anxiety, depression, epilepsy, fragile X syndrome, Huntington’s disease, metabotropic glutamate receptors, pain, Parkinson’s disease, schizophrenia.

## Abstract

Glutamate is the main excitatory neurotransmitter in the central nervous system (CNS) and is a major player in complex brain functions. Glutamatergic transmission is primarily mediated by ionotropic glutamate receptors, which include NMDA, AMPA and kainate receptors. However, glutamate exerts modulatory actions through a family of metabotropic G-protein-coupled glutamate receptors (mGluRs). Dysfunctions of glutamatergic neurotransmission have been implicated in the etiology of several diseases. Therefore, pharmacological modulation of ionotropic glutamate receptors has been widely investigated as a potential therapeutic strategy for the treatment of several disorders associated with glutamatergic dysfunction. However, blockade of ionotropic glutamate receptors might be accompanied by severe side effects due to their vital role in many important physiological functions. A different strategy aimed at pharmacologically interfering with mGluR function has recently gained interest. Many subtype selective agonists and antagonists have been identified and widely used in preclinical studies as an attempt to elucidate the role of specific mGluRs subtypes in glutamatergic transmission. These studies have allowed linkage between specific subtypes and various physiological functions and more importantly to pathological states. This article reviews the currently available knowledge regarding the therapeutic potential of targeting mGluRs in the treatment of several CNS disorders, including schizophrenia, addiction, major depressive disorder and anxiety, Fragile X Syndrome, Parkinson’s disease, Alzheimer’s disease and pain.

## INTRODUCTION

Glutamate is the main excitatory neurotransmitter in the central nervous system (CNS) and exerts its effects through the activation of several receptor subtypes. Glutamate mediates fast excitatory synaptic transmission between neurons through the ionotropic receptors α-amino-3-hydroxy-5-methyl-4-isoazolepropionicacid (AMPA), N-methyl-d-aspartate (NMDA) and kainate receptors. Furthermore, glutamate exerts a prominent modulatory role of the fast excitatory tone set by the ionotropic receptors by activation of the metabotropic glutamate receptor (mGluR) family. Ionotropic and metabotropic glutamate receptors interact in the fine tuning of neuronal responses under different conditions of activity and their co-localisation indicates that glutamate modulates neurotransmission and neuronal excitability at the same synapses [1,2]. In addition, mGluRs are also present at synapses releasing neurotransmitters other than glutamate.

The actions of glutamate on ionotropic receptors are responsible for numerous physiological processes, including basic neuronal communication, axonal pathfinding, mood regulation, and memory formation. It has been suggested that dysregulation of the glutamatergic system is implicated in a variety of psychiatric and neurological disorders such as schizophrenia, major depression disorder, and Parkinson’s disease. In this review some basic features of mGluRs will briefly be revisited before a comprehensive overview of their therapeutic potential for the treatment of several psychiatric and neurological disorders will be discussed.

### Classification, Structure, and Function

Metabotropic glutamate receptors belong to the superfamily of G-protein-coupled receptors. Structurally, mGluRs are formed by a large extracellular N-terminal domain containing the glutamate binding site and seven α-helical transmembrane segments [3]. The most conserved domains between the different mGluRs subtypes are the site involved in coupling to the G-protein and the glutamate binding site [2]. Functional mGluRs comprise homodimers stabilized by both an inter-subunit disulphide bond and hydrophobic interactions [2]. Eight different metabotropic receptors have been cloned (mGluRs 1-8). They are classified into three groups according to their sequence homology, pharmacological properties and intracellular signal transduction pathways Fig. (**1**). Group I consists of mGluR1 and mGluR5, and are positively coupled to phospholipase C through G-proteins of the G_q_/G_11_ type. Activation of group I receptors leads to stimulation of phospholipase C, production of inositol triphosphate, release of Ca^2+^ from intracellular stores and production of diacylglycerol, which in turn activates protein kinase C [4-6]. Group II, consisting of mGluR2 and mGluR3, and Group III, consisting of mGluR4, mGluR6, mGluR7 and mGluR8, are coupled to the inhibition of adenylyl cyclase activity through G-proteins of the G_i_/G_0_ type. Approximately 70% sequence homology exists within each group of mGluRs, while approximately 45% sequence homology has been described between groups. Alternative splice variants have also been described for mGluR1, mGluR4, mGluR5, and mGluR7 [7].

### Expression Pattern and Subcellular Localization 

#### Group I mGluRs

Immunohistochemical studies have shown that high levels of mGluR1 are present in the hippocampus, globus pallidus, substantia nigra, thalamus, cerebellum and the olfactory bulb [7,8]. Lower levels of mGluR1 have also been found in the striatum, neocortex, and hypothalamus [7,9,10]. The mGluR5s are expressed in the cortex, striatum, caudate nucleus, nucleus accumbens and septum, as well as in the hippocampus and olfactory bulb, where they are highly co-expressed with mGluR1. Lower levels have been described in the cerebellum and thalamus. Both mGluR1 and mGluR5 are mainly concentrated in postsynaptic structures [11,12] and with a few exceptions almost undetectable in presynaptic structures [9,13]. At the subcellular level, mGluR5s are found both synaptically and extrasynaptically on postsynaptic spines of principal neurons [9,12-14] Fig. (**2**).

#### Group II mGluRs

Both mGluR2s and mGluR3s are highly expressed in the hippocampus, cortex, nucleus accumbens, striatum and amygdala. In addition, mGluR2s are found at high levels in the caudate nucleus, cerebellar cortex and olfactory bulb, while mGluR3s are enriched in the septum and substantia nigra [14]. In the hippocampus, mGluR2s are mainly found at presynaptic sites [15,16], whereas mGluR3s are mostly expressed postsynaptically [17]. Furthermore, presynaptically localized mGluR2s and mGluR3s are mostly found on axons and pre-terminal regions of both glutamatergic and GABAergic synapses [17]. In some brain structures, e.g. hippocampus, cortex, and striatum, mGluR2s and mGluR3s have been found at both presynaptic and postsynaptic sites [17-22]. Postsynaptically, mGluR2s are mostly concentrated in cell bodies and dendritic shafts, whereas mGluR3s are found to be perisynaptic on spines [14,17] Fig. (**2**).

#### Group III mGluRs

The mGluR4s show a widespread brain distribution, but high distribution levels are only reported in the hippocampus and cerebellar cortex, while the expression of mGluR6s is limited to the retina [23]. The mGluR7s have a widespread distribution and can be found at high levels in several brain regions including hippocampus, cortex, globus pallidus, amygdala, colliculi and olfactory bulb. Lower expression levels are found in the striatum, substantia nigra, caudate nucleus and nucleus accumbens. The mGluR8s are found predominantly in the hippocampus, hypothalamus and olfactory bulb, but at low levels.

Group III mGluRs mainly serve as presynaptic autoreceptors involved in reducing glutamate release from presynaptic terminals [16,24-26], and in contrast to Group II mGluRs they are mostly located synaptically. Furthermore, mGluR7s are not only localized within the presynaptic active zone [25,26], but also on GABAergic terminals Fig. (**2**). 

#### mGluRs in the Spinal Cord

Almost all mGluR subtypes have been described in the spinal cord [27-31]. In particular, both Group I and II mGluRs have been found in the soma and terminals of dorsal root ganglion neurons [32-34].

#### mGluRs in Non-Neuronal Cells

Several mGluR subtypes are expressed by non-neuronal brain cells, including microglia, oligodendrocytes and astrocytes. In microglial cells, activation of group III mGluRs activates a neuroprotective pathway [35], while activation of group II mGluRs induces an apoptotic pathway [35,36]. In oligodendrocytes, mGluRs have been suggested to be involved in the control of oxidative stress and excitotoxicity [37]. In astrocytes, mGluRs have been suggested to play a role in the modulation of synaptic transmission within neighboring neurons. For instance, activation of astrocytic mGluR5 induces calcium oscillations, in turn leading to glutamate release that can affect neurotransmission [38]. The activation of astrocytic mGluRs has also been shown to alter the expression of glutamate transporters [39], and stimulate the release of inflammatory cytokines [40].

### Molecular Mechanisms of Action

The mGluRs generally exert modulatory roles although some members of the family have been found to mediate synaptic transmission directly *via *activation of slow postsynaptic potentials [41]. Modulation is achieved either through activation of intracellular second messenger pathways, or through a direct action of the βγ subunits of the heterotrimeric G-proteins in modulating, e.g. ion channels activity [42]. Despite their widespread distribution in the brain, there is currently limited knowledge on how mGluRs modulate the physiological release of glutamate [43]. However, the discovery of selective mGluR ligands has revealed that mGluRs localised presynaptically modulate neurotransmitter release in a number of ways which include the modulation of presynaptic Ca^2+^ and K^+^ channels and regulation of the release machinery.

Group I mGluRs trigger a signalling cascade involving protein kinase and ultimately leading to the inhibition of presynaptic K^+^ channels, thereby delaying repolarisation of the nerve terminals [44,45]. They have also been shown to exert a positive effect by promoting the activity of presynaptic voltage dependent Ca^2+^ channels [46], although bidirectional actions have been reported [47]. A negative impact on transmitter release can also be exerted indirectly by postsynaptic Group I mGluRs through stimulation of endocannabinoid release and activation of presynaptic cannabinoid CB1 receptors [48]. 

Group II and III mGluRs generally act to reduce neurotransmitter release. For instance, their activation reduces glutamate release through depression of P/Q-type Ca^2+^ channels [49,50]. This might be achieved by a direct action of the dissociated G-protein βγ subunits [51]. The mGluR7 mediated depression of Ca^2+^ channel function requires phospholipase C or protein kinase C activity [52,53]. Additionally, the actions of mGluR7s are independent of cAMP and protein kinase A, although group III mGluRs typically operate by reducing cyclic AMP at the synapses between mossy fibres and stratum lucidum interneurons [1,52]. Inhibitory action of presynaptic mGluRs may involve a direct regulation of the release/exocytosis machinery downstream of Ca^2+^ entry. Indeed, the βγ subunits of G proteins can directly modulate release [54] through interactions with the SNARE complex [55,56] and also lead to a reduction in the number of active release sites [57].

### Modulation of Synaptic Plasticity

The mGluRs have been implicated in a number of synaptic plasticity phenomena, including short-term modification of synaptic strength, long-term depression (LTD) and long-term potentiation (LTP). 

#### Long-Term Depression

Activation of Group I mGluRs, either synaptically or pharmacologically, has been shown to induce long-term depression of synaptic transmission [58-64]. For instance, Group I mGluRs were found to play a critical role in the induction of long-term depression at the corticostriatal synapse, presumably by the regulation of intracellular calcium [65]. Some studies have suggested that the induced long-term depression was mediated indirectly through postsynaptic release of retrograde messengers [66]. 

A classical example of group II mGluR-mediated LTD is found at hippocampal mossy fiber synapses, where prolonged low-frequency stimulation activates Group II mGluRs localized preterminaly, causing a persistent reduction in glutamate release [67,68] that is exclusively presynaptic in nature. At this particular synapse, both LTD and LTP share a common mechanism and the same protocols that induce LTD through presynaptic mGluRs can be used to reverse LTP, a phenomenon termed depotentiation [69]. Group II mGluR-dependent depotentiation is also observed in the amygdala [70].

Presynaptic Group III mGluRs were shown to be involved in the induction of LTD at synapses between hippocampal mossy fibers and stratum lucidum interneurons, as well as at excitatory inputs onto stratum radiatum interneurons [49,52,71]. This form of plasticity, however, requires a rise in postsynaptic Ca^2+^ through Ca^2+^-permeable AMPA receptors.

The mechanisms of mGluR-dependent LTD are discussed in section “Fragile X Syndrome” [64,72].

#### Long-Term Potentiation

 The activation of mGluRs has also been shown to be required for both NMDA receptor-dependent and independent forms of LTP [73,74]. Group I mGluRs have been involved in the induction of LTP at the cortico-striatal synapse, where co-activation of both mGluR1 and mGluR5 is required, thereby contributing to a strong increase in intracellular calcium levels [75]. In addition, Group I mGluRs are required for the induction of a new form of LTP specific of NMDA receptors at hippocampal mossy fiber synapses [76,77]. In NMDA receptor-dependent LTP, mGluR activation apparently serves as a priming factor or molecular switch and mGluR5 [78-80], but not mGluR1 [81] seems to be critically involved. Some forms NMDA receptor-independent LTP also require mGluR activation [82,83] and Group I mGluRs seems to be the major player [81,84,85]. A recent report showed a role for Group I mGluRs in the induction of long-term plasticity of non-synaptic, synchronized neuronal activity [86]. A role for group II mGluRs in LTP has also been evidenced [87,88], whereas Group III mGluRs presumably have an indirect effect on LTP induction at mossy fiber synapses onto stratum lucidum interneurons, a process that has been attributed to presynaptic mGluR7 functioning as a metaplastic switch [52].

### Pharmacological Tools

In the present section, we will introduce compounds used in preclinical studies aimed at elucidating the role of specific mGluR subtypes in the pathophysiology of several psychiatric and neurological disorders (Table **1**). 

## SCHIZOPHRENIA

Schizophrenia is a psychiatric disorder affecting 1% of the population worldwide [89,90]. Symptoms associated with schizophrenia have traditionally been categorised into positive symptoms e.g. delusions and hallucinations, negative symptoms e.g. blunting of affect, social withdrawal, lack of motivation, cognitive deficits e.g. impairment in memory, executive function, working and long-term memory and affective symptoms [91]. The dopamine hypothesis of schizophrenia, implying a hyperactivity of the dopaminergic system, has been the main neurochemical hypothesis for many years [90,92]. Current antipsychotic drugs are all dopamine D_2_ receptor antagonists with varying degrees of potency, and while these drugs to some extent are effective against the positive symptoms, the effects on negative and cognitive symptoms are very limited. Furthermore, the treatment is often accompanied by a range of side-effects such as extrapyramidal motor symptoms and the metabolic syndrome [90]. There is increasing evidence that a primary glutamatergic dysfunction is associated with schizophrenia, leading secondarily to a dopaminergic imbalance. More precisely, a current model for the pathophysiology of schizophrenia involves a mechanism by which NMDA receptor hypofunction would induce a dysregulation of GABA fast-spiking interneurons in cortical areas, leading in turn to a disinhibition of pyramidal glutamatergic output and contributing to a reduction in the synchronized neuronal activity of these neurons [93,94]. Further support of the involvement of an altered glutamatergic neurotransmission in the pathophysiology of schizophrenia stems from the observation that the NMDA antagonist, ketamine, induces schizophrenia-like symptoms in healthy volunteers, including transient psychosis, disrupted affect, and cognitive deficits, and exacerbates specific symptoms in schizophrenic patients [95,96]. This growing body of evidence involving disturbances in glutamatergic transmission in schizophrenia is at the basis of an increasing effort aimed at modulating metabotropic glutamate receptors as a possible therapeutic strategy (Table **2**). 

### Selective Group I Modulation

Based on the purported disinhibition of pyramidal glutamatergic output as a core feature of schizophrenia, modulation of glutamate signalling at the postsynaptic levels *via *group I metabotropic receptors, and in particular mGluR5s, has gained interest as a potential promising therapeutic strategy.

#### mGluR1

There is only limited evidence that mGluR1 positive modulation may be of interest for the treatment of schizophrenia. On one hand, increased mGluR1 expression has been reported in the prefrontal cortex of schizophrenics [97]. In addition, mGluR1 knockout mice have impaired sensorimotor gating as assessed in a prepulse inhibition paradigm [98], a deficit also encountered in schizophrenic patients. However, the sensorimotor gating deficits observed in mGluR1 knockout mice were only reversed by lamotrigine, an anticonvulsant drug used in the treatment of bipolar disorder, but not by a classical antipsychotic. Pharmacological modulation of mGluR1s using the selective antagonist EMQMCM has no effect on either MK-801-induced locomotor hyperactivity or MK-801-induced deficit in prepulse inhibition [99]. These observations further weaken a potential link between mGluR1 and psychotic symptoms. 

#### mGluR5 

Extensive evidence exists in the literature supporting a therapeutic potential of mGluR5 positive modulation for the treatment of schizophrenia. The mGluR5s are expressed in both GABAergic interneurons and pyramidal neurons in cortical and hippocampal areas, and are functionally coupled to NMDA receptors. Activation of mGluR5 leads to potentiation of NMDA receptor-mediated currents in cortical areas [100]. Thus, based on the NMDA hypofunction hypothesis of schizophrenia, positive modulation of mGluR5 might provide a viable approach to restore NMDA receptor function. In line with this assumption, mGluR5 knockout mice display locomotor hyperactivity in response to a novel environment as well as in response to MK-801, an NMDA antagonist, sensorimotor gating deficits in a prepulse inhibition paradigm, and short-term memory deficit in a Y-maze test [101]. Studies using pharmacological modulation of mGluR5s further strengthen the involvement of mGluR5 in the pathophysiology of schizophrenia. For instance, the selective mGluR5 antagonist MPEP was found to potentiate MK-801-induced locomotor hyperactivity and stereotypies [102], indicative of a pro-psychotic effect. On the other hand, amphetamine-disrupted PPI in rats was found to be reversed by a selective positive allosteric modulator of mGluR5, compound 8q [103]. Recently, another selective mGluR5 allosteric potentiator, ADX47273, was reported to reduce conditioned avoidance responding in rats, a standard screening model for antipsychotic efficacy, as well as prevent PCP- and amphetamine-induced locomotor hyperactivity, suggestive of an antipsychotic potential [104]. The antipsychotic potential of mGluR5 positive modulators may arise from a modulatory effect on the mesolimbic dopaminergic pathway, as suggested by the decrease in basal dopamine levels in the nucleus accumbens induced by ADX47273 [105]. Additionally, mGluR5 agonism has also been shown to modulate the ventral striatopallidal GABAergic pathway [102], which is a common target for antipsychotic drugs.

With respect to modulation of cognitive function, blockade of mGluR5s by MPEP has been reported to potentiate MK-801-induced impairments of spatial working memory and instrumental learning in rats [102]. Conversely, allosteric potentiation of mGluR5s using ADX47273 was found to enhance recall after a 48h delay in a novel object recognition task [104]. A modulatory role of mGluR5s on cognitive function is also indicated by the finding that CDPPB, another selective mGluR5 positive allosteric modulator, reduces the characteristic set-shifting impairment induced by NMDA receptor blockade in rats [106]. The modulatory effect of mGluR5s on cognitive processing has been hypothesized to rely on changes in neuronal activity in the prefrontal cortex. In fact, inhibition of mGluR5s by MPEP was reported to decrease burst firing activity of cortical neurons in awake rats and potentiated the increase in firing rate induced by NMDA receptor blockade [107]. Conversely, the mGluR5 positive allosteric modulator CDPPB was shown to increase bursting in PFC neurons in awake rats, and to prevent the robust excitatory effect of NMDA receptor blockade by MK-801 [108]. Taken together, these studies indicate that mGluR5 positive modulation may be effective in ameliorating cognitive dysfunction induced by NMDA hypofunction by restoring the function of prefrontal cortical neurons, and thereby be of clinical usefulness for the treatment of cognitive symptoms associated with schizophrenia.

Besides an antipsychotic and procognitive potential of mGluR5 positive modulation, the selective mGluR5 antagonist, MPEP, has interestingly been reported to induce social interaction deficits in rats, suggesting a potential link between mGluR5 and social deficits characteristic of negative symptoms in schizophrenia [109].

Besides the above-mentioned preclinical evidence, an involvement of the GRM5 gene in schizophrenia has been suggested in a genetic study showing a significant difference in allele frequency distribution between schizophrenics and controls [110]. An increased neuronal mGluR5 expression has also been reported in the pyramidal cell layer in the prefrontal cortex of schizophrenics [111].

In summary, mGluR5 positive modulation may be a promising therapeutic strategy for the treatment of positive and cognitive symptoms associated with schizophrenia. Moreover, a potential benefit on negative symptoms, and particularly on social withdrawal, may also be achieved, although additional studies with positive modulators would be required to draw any conclusion in this regard.

### Selective Group II Modulation

Stimulation of group II receptors inhibits synaptic release of glutamate presynaptically [112]. A considerable amount of evidence supports a therapeutic potential of group II receptor agonism for the treatment of schizophrenia. Recently, clinical proof of concept has been obtained in schizophrenic patients [113], thereby identifying mGluR2/3 modulation as one of the most attractive strategies amongst all mGluRs for the treatment of schizophrenia. The first preclinical evidence was obtained using the highly selective agonist of group II mGluR, LY354740. In these early studies, activation of mGluR2/3 with LY354740 was found to block phencyclidine-induced glutamate efflux in the nucleus accumbens and prefrontal cortex as well as phencyclidine-induced locomotor hyperactivity in rats [114]. Interestingly, dopaminergic neurotransmission was not affected by LY354740. Another selective mGluR2/3 receptor agonist with improved bioavailability, LY404039, was also reported to produce similar antipsychotic effects in psychostimulant-induced locomotor hyperactivity as well as in a conditioned avoidance response paradigm without producing motor impairment [115]. Recently, selective positive allosteric modulators of mGluR2 have been identified, and have been shown to reduce NMDA blockade-induced hyperactivity in rats [116,117]. Another selective mGluR2 positive allosteric modulator, BINA, was found to block locomotor hyperactivity induced by phencyclidine, but not by amphetamine, as well as prevent phencyclidine-induced disruption in sensorimotor gating in mice [118]. In line with this assumption, the attenuation of phencyclidine- and amphetamine-induced locomotor hyperactivity produced by the mGluR2/3 agonists, LY379268 and LY404039, was completely abolished in mGluR2 knockout mice whereas it was not affected in mGluR3 knockout mice [119,120]. Taken together, these findings suggest that targeting mGluR2s, rather than mGluR3s, is a relevant mechanism for the treatment of schizophrenia. 

Few preclinical studies have suggested a procognitive potential of mGluR2/3 agonism. For instance, LY354740 reversed cognitive deficits induced by phencyclidine in a delayed alternation task in rats [114]. Both mGluR2/3 agonism using LY354740 and selective allosteric potentiation of mGluR2 using LY487379 was reported to attenuate deficits in social discrimination induced by neonatal phencyclidine in rats, an effect attributed to a selective attention deficit, while no effect of either compound was found on the total time spent in social interaction [121]. However, others have reported that mGluR2/3 agonism produced no effect or impairment in cognitive function. For instance, LY354740 was found to impair performance in a delayed alternation task in rats, while it had no effect on phencyclidine-induced deficits in the same task [122]. The reason for these discrepancies has not been clearly established.

Neurons in the prefrontal cortex are assumed to play a critical role in the cognitive impairing and psychotomimetic effects of NMDA receptor antagonists. In this respect, it has been hypothesized that the putative antipsychotic and procognitive effects of mGluR2/3 agonism would attenuate the disruption of neuronal activity in the prefrontal cortex produced by NMDA hypofunction. In fact, stimulation of mGluR2/3 by LY354740 was reported to decrease the firing rate and increase burst firing of pyramidal cells in the prefrontal cortex in awake rats, while it was able to reverse the increased firing rate and decreased burst firing induced by the NMDA receptor antagonist MK-801 [123]. 

Ketamine is a non-competitive NMDA antagonist widely used in clinical studies as it induces schizophrenia-like symptoms, including hallucinations, delusions, and cognitive impairment [95]. For instance, it has been shown to disrupt working memory in humans at subanesthetic doses, an effect attributed to NMDA receptor blockade in the prefrontal cortex. Interestingly, LY354740 was reported to attenuate working memory deficits induced by ketamine administration in healthy volunteers [124]. A study in post-mortem brain tissues from schizophrenic patients using an antibody directed against mGluR2 and 3 also revealed an increased mGluR2/3 expression in the prefrontal cortex [97]. It was shown recently that mGluR2 expression, rather than mGluR3, was increased in the prefrontal cortex of schizophrenics [125]. Interestingly, an oral prodrug of LY404039 (LY2140023) was evaluated in schizophrenic patients. Treated patients showed significant improvement in both positive and negative symptoms compared to placebo [113]. 

In summary, stimulation of mGluR2/3, and most likely selectively mGluR2, represents an attractive therapeutic strategy for the treatment of schizophrenia. More precisely, beneficial effects have been observed on both positive and negative symptoms in schizophrenic patients [113]. In addition, a beneficial effect on working memory has also been reported in humans [124], further suggesting that improvement of cognitive symptoms associated to schizophrenia may also be achieved. However, this clinical endpoint would need to be specially considered in future schizophrenia trials.

### Selective Group III Modulation

#### mGluR7 

Few genetic studies have suggested a role of mGluR7 in the pathophysiology of schizophrenia. GRM7 analysis in a population of Japanese schizophrenics identified a single nucleotide polymorphism (SNP) with lower promoter activity, suggesting that lower expression of mGluR7 may increase the risk of developing schizophrenia [126]. Another study from a Japanese sample has also identified highly significant association of schizophrenia with the two other SNPs in GRM7 [127]. A mutation in PICK-1 protein, which is crucial for clustering of mGluR7 at presynaptic release sites, has also been associated with schizophrenia [128].

In addition to these genetic findings, mGluR7 have been shown to modulate the mesolimbic dopaminergic system and the ventral striatopallidal feedback loop, suggesting a potential link to positive symptoms of schizophrenia. Interestingly, the selective mGluR7 antagonist, AMN082, was reported to decrease the frequency of mEPSCs in dopaminergic neurons of the ventral tegmental area *in vitro* [129]. Since glutamatergic innervation of the ventral tegmental area plays a critical role in burst firing of dopaminergic neurons, the ability of mGluR7 to modulate these excitatory inputs may be indicative of an antipsychotic potential of mGluR7 agonism. However, AMN082 did not affect basal or cocaine-induced increase in dopamine levels in the nucleus accumbens, while it decreased GABA and increased glutamate levels [130,131]. The effect of AMN082 on glutamate levels was further shown to be partly mediated through reduction of GABA levels. In line with the absence of modulation of dopamine levels by mGluR7 activation, AMN082 did not affect basal or cocaine-induced locomotor hyperactivity in rats [131]. However, AMN082 was found to block cocaine-induced decrease in GABA levels in the ventral pallidum. In summary, mGluR7 agonism does not seem to affect dopaminergic neurotransmission in the nucleus accumbens, but is able to modulate the ventral striatopallidal pathway in condition of excessive dopaminergic tone in the nucleus accumbens, which may be relevant to the treatment of positive symptoms. 

Several studies have also suggested that mGluR7 may regulate cognitive function. For instance, spatial and working memory has been investigated in mGluR7 knockout mice [132]. In a Morris water maze task, mGluR7 knockout mice show a significant delay in acquiring the location of the hidden platform, as well as in recall during the probe trial. In a working memory version of the Morris water maze, mGluR7 knockout mice were impaired and consistently slower to solve the matching-to-position task, possibly due to impairment in short-term memory. In the consecutive extinction trials, mGluR7 knockout mice were also delayed to adopt a new search strategy. Taken together, these data suggest that mGluR7 knockout mice have impaired reference memory acquisition and spatial working memory, and a dysfunctional glutamatergic signalling particularly in the hippocampus and prefrontal cortex where mGluR7 are expressed has been hypothesized to cause these deficits. Performances in complex working memory tasks such as 8-arm radial maze task were also impaired in mGluR7 knockout mice [133]. Interestingly, the working memory deficit was associated with an increased hippocampal theta power while performing the task, which was suggested to reflect a lack of modulation of local inhibition, in turn leading to decreased neuronal firing threshold and altered spike timing [134]. At the cellular level, mGluR7 knockout mice were reported to exhibit deficits in short-term, but not long-term potentiation in the hippocampus [135], findings in agreement with the hypothesis that short-term potentiation represents the cellular substrate for short-term memory and critical for working memory performances. 

Taken together, these findings indicate that mGluR7 positive modulation may represent a new therapeutic strategy potentially beneficial for the treatment of positive as well as cognitive symptoms. In addition, since mGluR7s are also highly expressed in the amygdala and have been implicated in anxiety (see “Major Depression Disorder and Anxiety”), a potential effect on negative symptoms might also be achieved. However, since the lines of evidence rely on the use of knockout mice and a single pharmacological tool, additional studies using other selective positive or negative modulators of mGluR7 would be needed. 

#### mGluR8 

In a genetic study, one susceptibility locus for schizophrenia was identified within the GRM8 region in Japanese [136], suggesting that mGluR8 may have a therapeutic potential. In this respect, the antipsychotic potential of mGluR8 modulation was investigated in a preclinical study. While the selective mGluR8 agonist (S)-3,4-DCPG was devoid of any effect on PCP-or amphetamine-induced locomotor hyperactivity in rats when administered systemically, intracerebroventricular (S)-3,4-DCPG administration prevented amphetamine-induced locomotor hyperactivity in mice, although a trend to reduce spontaneous locomotor activity was also observed [137]. In addition, mGluR8 knockout mice did not show any change in locomotor activity or deficit in sensorimotor gating as assessed in a prepulse inhibition of the startle response paradigm, further ruling out a possible antipsychotic effect of mGluR8 modulation. However, the same study reported that mGluR8 knockout mice exhibit an anxiogenic phenotype as assessed in an open field as well as an elevated plus maze, suggesting that mGluR8 modulation might be relevant for negative symptoms of schizophrenia. 

## ADDICTION

Drug addiction is a chronic illness arising as a consequence of frequent drug taking. It involves the progression from acute drug use to the development of drug-seeking behavior, the vulnerability to relapse, and the decreased, slowed ability to respond to naturally rewarding stimuli. Several lines of evidence suggest that glutamate neurotransmission plays a key role in the processes leading to drug addiction and relapse. In particular, hyperglutamatergic response in the corticostriatal pathway in response to drug-related cues and leading to drug-seeking behaviour is believed to be a core component underlying relapse.

Glutamate homeostasis refers to the regulation of extracellular glutamate levels in the synaptic and extrasynaptic spaces and is highly dependent on the balance between glial and synaptic glutamate release and elimination. Impairment in glutamate homeostasis leads in turn to altered synaptic activity and plasticity by affecting glutamate levels available for stimulation of ionotropic and metabotropic glutamate receptors. Preclinical studies have shown that chronic administration of several drug of abuse, including cocaine, heroin and nicotine, induced neuroadaptative changes at the glutamatergic synapse. These changes include reduced basal extrasynaptic glutamate levels, presumably as a consequence of reduced expression of the glial cystine-glutamate exchanger, in turn leading to down-regulation of presynaptic mGluR2/3 [138,139]. Since presynaptic mGlu2/3 regulates synaptic glutamate release, its down-regulation has been suggested to result in enhanced synaptic glutamate release during cue-, stress- or drug-induced reinstatement. This increased synaptic glutamate transmission results in post-synaptic adaptations, including up-regulation of AMPA Glu1 receptors (GRIA1), and a compensatory down-regulation of mGluR1/5, ultimately leading to neuroplasticity. These glutamate-induced neuroplastic changes have been suggested to be the common factor in relapse for many types of drugs. In addition, preclinical evidence supports the occurrence of similar glutamate dependent-neuroplastic alterations in other key brain areas such as the ventral tegmental area and the amygdala [140,141] (Table **3**). 

### Selective Group I Modulation

#### mGluR1

Only a few preclinical studies have investigated the effect of selective mGluR1 negative allosteric modulators on drug-related behaviours. For instance, the mGluR1 antagonist, CPCCPOEt, was shown to reduce measures of ethanol reward in an operant self-administration paradigm, block the expression of ethanol-induced place conditioning, as well as ethanol consumption under 24-h free-access conditions in alcohol-preferring mice [142]. At the neurochemical level, CPCCPOEt was found to inhibit dopamine and glutamate increases induced by ethanol administration in the nucleus accumbens, while it facilitated GABA-induced release, suggesting that mGluR1a blockade may regulate the rewarding properties of ethanol by facilitating its inhibitory effect in the nucleus accumbens. In a cocaine sensitization paradigm, EMQMCM, another mGluR1 negative allosteric modulator, was reported to reduce the expression of the sensitized ambulatory motor activity of cocaine-experienced rats acutely challenged with cocaine [143,144]. However, the same dose range of EMQMCM also reduced ambulatory horizontal activity in control animals, which may confound the apparent reduction observed in sensitized animals. Interestingly, another mGluR1 antagonist, JNJ 16259685, infused locally in the dorsal hippocampus was recently reported to block drug context-induced reinstatement of cocaine-seeking behaviour in rats, while it did not alter locomotor activity or food-reinforced instrumental behavior [145]. In another report, the role of mGluR1 in the reinstatement of nicotine-seeking behaviour in rats was investigated [146]. In this study, blockade of mGluR1 by EMQMCM (5 and 10 mg/kg) was found to prevent both cue-induced and nicotine priming-induced reinstatement of nicotine-seeking behaviour. However, the highest dose of EMQMCM (10 mg/kg) also inhibited cue-induced reinstatement of food-seeking behaviour. This observation is in contrast to that reported by Xie *et al.* [145] showing that mGluR1 blockade selectively in the hippocampus did not affect food-reinforced instrumental behaviour, but may indicate that non regional selective mGluR1 blockade may not only inhibit the motivational value of drugs, but also that of natural reinforcers. In another study, blockade of mGluR1 by EMQMCM (5, 10 and 20 mg/kg) was reported to prevent the expression of sensitization to the locomotor effect of morphine, but did not affect the expression of withdrawal syndrome in mice [147], further suggesting that mGluR1 blockade may be of potential benefit in the treatment of opiate seeking behaviour.

In conclusion, preclinical evidence suggests that mGluR1 blockade may represent a potential pharmacological mechanism relevant for the treatment of drug addiction, including alcohol, cocaine, nicotine, and opiates. However, one concern might be that such a mechanism would also affect the motivational value of natural reinforcers. 

#### mGluR5

Numerous preclinical studies have investigated the role of mGluR5 in drug-related behaviours. For instance, the mGluR5 antagonist, MPEP, was shown to reduce measures of ethanol reward in an operant self-administration paradigm, block the expression of ethanol-induced place conditioning, as well as ethanol consumption under 24-h free-access conditions in alcohol-preferring mice [142]. Along the same line, mGluR5 null mutant mice show decreased alcohol consumption [148]. In addition, MPEP was reported to block cue-induced reinstatement of alcohol-seeking behavior in alcohol-preferring rats [149], further strengthening a potential benefit of mGluR5 blockade in alcohol addiction. With respect to other drugs of abuse, MPEP has been shown to block the expression of sensitization to the locomotor effect of nicotine, as well as reduce nicotine-induced drug-seeking behaviour in a model of nicotine-triggered relapse to nicotine-seeking in rats [150]. Blockade of mGluR5 has also been reported to affect cocaine-induced behaviours. In particular, both acute and chronic blockade of mGluR5 by MPEP attenuate cocaine self-administration in squirrel monkeys [151,152]. Acute MPEP treatment has also been shown to prevent cocaine-induced reinstatement of drug seeking in squirrel monkeys [151], as well as block the expression of cocaine sensitization in rats [150]. In contrast to the latter findings, another mGluR5 antagonist, MTEP, was devoid of effect on the expression of cocaine sensitization in rats [143]. It is generally agreed that MTEP exhibits superior selectivity for mGluR5 compared to MPEP, with fewer off- targets identified compared to the former [153]. Of particular interest is the observation that MPEP is able to interact functionally with NMDA receptors in rat [154]. Therefore, the effects of MPEP should be interpreted cautiously, especially when considering behaviours expected to be affected by NMDA receptors. Another study investigating the role of mGluR5 in methamphetamine-induced behaviours revealed that MTEP dose-dependently reduced the reinforcing effects of methamphetamine and attenuated cue- and drug-induced reinstatement of methamphetamine-seeking behaviour in rats [155]. Blockade of mGluR5 with MTEP was further found to inhibit the expression of morphine sensitization [147] as well naloxone-induced symptoms of morphine withdrawal in morphine-dependent mice [147,156]. Interestingly, blockade of mGluR5 has been shown to be devoid of effect on food-motivated instrumental behaviour and cue-induced reinstatement of food-seeking [152,155]. Taken together, these findings suggest that selective blockade of mGluR5 might represent an interesting mechanism for the treatment of addictive behaviours to various drug classes. With contrast to mGluR1 blockade, no effect on the motivational value of natural reinforcers would be expected based on the available preclinical data.

### Selective Group II Modulation

Presynaptic mGluR2/3s act as autoreceptors, their stimulation attenuating synaptic glutamate release. Agonising mGluR2/3 has been suggested to be of potential interest for the treatment of drug addiction. The recent discovery of selective mGluR2/3 agonists has offered the opportunity to test this hypothesis in preclinical models. LY354740 has been shown to block naloxone-induced symptoms of morphine withdrawal in morphine-dependent mice [157]. Another mGluR2/3 agonist, LY379268, was reported to attenuate ethanol self-administration and cue-induced reinstatement of ethanol seeking, although at doses that also reduced spontaneous locomotor activity [158]. The same mGluR2/3 agonist was found to block drug-induced reinstatement of cocaine seeking in rats [159]. In the latter study, LY379268 was also found to attenuate reinstatement of food seeking, although at doses 3 times higher than those effective in reinstatement of cocaine seeking. These nonspecific inhibitory effects on responding for natural rewards at higher doses of LY379268 have been reported by others [160-162]. Whether these effects are a general consequence of stimulating mGluR2/3 or whether they are compound specific is currently unknown. Stimulation of mGluR2/3 by LY379268 was further found to prevent nicotine self-administration and drug-induced reinstatement of nicotine seeking in rats [162], as well as stress- and drug context-induced reinstatement of ethanol seeking in rats [163]. It was also reported to be effective in preventing cue-induced reinstatement of heroin seeking [161]. However, a tolerance to the effect of LY379268 on nicotine self-administration was observed after daily administration for 14 days [162].

Studies using intracerebral administration of mGluR2/3 agonists have also identified critical brain areas involved in the modulation of drug seeking and relapse-like behaviours by mGluR2/3. For instance, LY379268 administered into the ventral tegmental area or the nucleus accumbens blocked reinstatement of heroin and cocaine seeking [159,161,164] and nicotine self-administration [162], further supporting the importance of mGluR2/3 located at excitatory synapses in the nucleus accumbens as well as on mesolimbic dopaminergic neurons in the ventral tegmental area.

In summary, mGluR2/3 agonism might represent a novel approach for the treatment of addiction to various drugs, including ethanol, cocaine, opiates and nicotine. A potential concern may arise from the observation that mGluR2/3 agonism induced an inhibitory effect on the motivational value of natural rewards in animals and may need further clarification. 

### Selective Group III Modulation

Similarly to metabotropic receptors of group II, subtypes belonging to group III are also located presynaptically, where they act as autoreceptors, regulating synaptic glutamate release. One major difference however between the two groups is their subcellular localization. While group II receptors are mostly perisynaptic, group III receptors are mainly expressed within the synapse. Evidence linking mGluRs belonging to group III to drug addiction is only limited, including a potential role of mGluR7 and mGluR8 subtypes, but may still be of possible value. 

Glutamate receptor metabotropic 7 (Grm7) has been identified as a candidate risk gene linked to alcohol addiction, as suggested by the observation that Grm7 mRNA expression levels in different mouse strains were inversely correlated to their alcohol consumption in a preference drinking behaviour paradigm [165]. In a recent report, the selective mGluR7 agonist, AMN082, was found to inhibit the rewarding effects of cocaine, while the rewarding effects of a natural reinforcer were not affected [131]. AMN082 injection in the nucleus accumbens or the ventral pallidum mimicked the effects observed after systemic administration. In addition, activation of mGluR7 by AMN082 was found to prevent cocaine-induced inhibition of GABA release in the ventral pallidum. Taken together, these data suggest that mGluR7 modulate cocaine reward by regulating GABAergic transmission in the ventral striatopallidal pathway. The recent availability of selective ligands for mGluR7 will hopefully help to further evaluate the therapeutic potential of mGluR7 modulation in the treatment of addictive behaviours.

A genome-wide association approach has identified Grm8, the gene encoding mGluR8, as having a strong association with heroin addiction [166]. Only few preclinical studies have investigated the role of mGluR8 in drug-induced behaviours. Amongst these, the reported selective mGluR8 agonist, (S)-3,4-DCPG, was demonstrated to attenuate ethanol self-administration and cue-induced reinstatement of ethanol seeking, although at doses that also reduced spontaneous locomotor activity [158]. In conclusion, there is only a very weak biological rationale supporting a potential interest of mGluR8 agonism for the treatment of drug addiction. 

## MAJOR DEPRESSION DISORDER AND ANXIETY

Major depressive disorder (MDD) and anxiety disorders are severe, disabling diseases that are highly prevalent and associated with negative impact on medical health, life quality and productivity [167-170]. Preclinical and clinical evidence suggest that MDD arises due to a decreased availability of serotonin and norepinephrine, since tricyclic antidepressants such as imipramine block the transporters for serotonin and norepinephrine leading to increased levels of serotonin and norepinephrine in the cerebrospinal fluid [171,172]. Furthermore, anxiety is believed to result mainly from a hyperactive state of the serotonergic system, where especially dysfunction of the 5-HT_1A _receptors is of significance [173]. The introduction of selective serotonin reuptake inhibitors (SSRIs) and combined serotonin and norepinephrine reuptake inhibitors (SNRIs) into clinical practice led to an improvement in the treatment of MDD and anxiety disorders by producing therapeutic benefit without the serious side-effects associated with the older tricyclic antidepressants [174]. Although SSRIs and SNRIs are effective, a meaningful therapeutic improvement is only apparent after several weeks of treatment [175]. Furthermore, many depressed patients respond only partially and a substantial proportion of patients fail to respond at all to first-line treatment [168]. Moreover, in those patients that do respond, side-effects such as sexual dysfunction, sleep disturbances and gastrointestinal disturbances have been reported [168]. During the past years various data support the idea that compounds working through multitarget mechanisms will have a better effect on both cardinal and comorbid symptoms of depression than selective compounds [176]. The above studies emphasize the need for improved treatment against MDD working through new mechanisms of action, either as monotherapy or add-on therapy.

Interestingly, the NMDA receptor antagonist, ketamine, is effective acutely in treatment-resistant depression [177], suggesting that improved treatment for MDD may be possible by targeting the glutamatergic neurotransmission (Table **4**).

### Selective Group I Modulation

Group I mGluR activation in rat hippocampus by DHPG was decreased after subchronic treatment with the antidepressant treatments, electroconvulsant stimulation and imipramine [178,179]. The immunoreactivity of both hippocampal mGluR1 and mGluR5 was upregulated following subchronic electroconvulsant stimulation [180]. The observed receptor upregulations may reflect a compensatory mechanism caused by the receptor subsensitivity provoked the antidepressant treatment. 

Although effects of mGluR1 antagonists have not been investigated as frequently as mGluR5 antagonists for anxiolytic and antidepressant effects, recent studies reported that the selective mGluR1 antagonists, JNJ16259685 and AIDA, exerted anxiolytic-like effect in the rat lick suppression test [181] and blocked anxiogenic behaviour in the rat light-dark test and open-field test [182], respectively.

#### mGluR5 

The potential use of mGluR5 negative modulators for the treatment of anxiety and depression has been broadly investigated. The mGluR5 antagonist, MPEP was shown to exert anxiolytic-like effect in several anxiety-like behaviour tests, including elevated-plus maze, social exploration, fear-potentiated startle, Vogel-conflict and light-dark box test [182-187]. In addition, MTEP exerted anxiolytic-like effects in contextual fear conditioning following acute or subchronic treatment, indicating that tolerance does not develop to the anxiolytic effect of MTEP [188]. Furthermore, it has been suggested that mGluR5 antagonism exerts its anxiolytic effect in the conditioned emotional response paradigm by a mechanism different from that of diazepam [189].

In the chronic mild stress model, a validated model to screen for antidepressant activity, increased expression of hippocampal mGluR5 has been reported [190]. It has been demonstrated that the mGluR5 antagonists, MPEP and MTEP, shortened the immobility time in the tail-suspension test and forced-swim test in mice [186,191-193], indicative of an antidepressant-like effect. Both MPEP and MTEP displayed antidepressant-like effects in an animal model of depression, the olfactory bulbectomy model [191,194]. Interestingly, it has been reported that mGluR5 knockout mice display an antidepressant-like behavioural phenotype [193]. In these mice imipramine, but not MPEP, exerted an antidepressant effect [193]. In the same study a synergy of MPEP and imipramine was observed in the mouse forced-swim test. The above studies indicate that mGluR1 and mGluR5 antagonists may have therapeutic potential in the treatment of MDD and anxiety disorders. Moreover, the antidepressant efficacy of tricyclic antidepressants and SSRIs might be enhanced by concomitant treatment with mGluR5 negative modulators.

### Selective Group II Modulation

A human postmortem study showed increased levels of mGluR2/3 in samples from the prefrontal cortex [195]. In a Japanese population an association between GRM3 (group II mGluR gene) and MDD has been reported [196]. Variations in GRM3 have been found to affect prefrontal glutamatergic neurotransmission and cognitive functions [197]. Preliminary evidence suggests that hippocampal levels of mGluR2/3 are decreased in the olfactory bulbectomy model [198] and in depressed flinders sensitive line rats [199]. However, the functional consequences of these alterations are unknown.

An association between classical antidepressants and mGluR2/3 compounds might exist. Chronic imipramine treatment was observed to reduce mGluR2/3 agonist-mediated inhibition of forskolin-stimulated cAMP production in rat hippocampus [200]. Moreover, administration of the selective mGluR2/3 antagonists, LY341495 and MGS0039, increased the firing rate of serotonergic dorsal raphe neurones [201], and administration of MGS0039 increased extracellular levels of serotonin in the rat prefrontal cortex [202]. Chronic treatment with MGS0039, furthermore, increased hippocampal neurogenesis [203], a mechanism demonstrated for some of the current antidepressants [204].

Both LY341495 and MGS0039 reduced immobility and in the tail-suspension test and forced-swim test [205,206]. In the forced-swim test, the two compounds increased swimming behaviour without affecting climbing behaviour [206]. Those antidepressants that increase serotonergic neurotransmission predominantly increase swimming behaviour whereas those that increase catacholaminergic neurotransmission increase climbing behaviour [207]. Thus, mGluR2/3 compounds may indirectly interact with the serotonergic neurotransmission. mGluR2/3 knockout mice displayed antidepressant-like behaviour, i.e. increased mobility, in the forced-swim test but not in the tail suspension test [208]. It has been reported that following administration of imipramine in combination with LY-341495 neuroadaptations to imipramine occurred faster than with imipramine alone [209], indicating that mGluR2/3 antagonism may shorten the lag time required to obtain full therapeutic improvement of current antidepressant, which is only apparent after several weeks of treatment [174].

LY341495 was effective in the marble burying test in mice [210]. However, in the same study LY341495 had no effects in the elevated plus maze and stress-induced hyperthermia tests in mice or on punished drinking. Thus, the behavioural profile of an mGluR2/3 antagonist seems to be different from that of conventional anxiolytic and antidepressant drugs. However, MGS0039 was reported to show anxiolytic-like effects in the conditioned fear model [211], indicating that mGluR2/3 antagonists may be beneficial in the treatment of some anxiety disorders.

Despite the fact that mGluR2/3 antagonists affect serotonergic activity the above studies suggest that mGluR2/3 antagonists work through different mechanism of actions compared to those of SSRIs and SNRIs and may provide beneficial effects in the treatment of MDD and anxiety disorders. 

### Selective Group III Modulation

Chronic treatment with citalopram, but not imipramine, decreased immunoreactivity of mGluR7 but not mGluR4 in the rat hippocampus and cerebral cortex [212]. However, the same study reported that chronic citalopram or imipramine did not change the effect of ACPT-1, a group III mGluR agonist, on forskolin-stimulated cAMP production. The lack of effect of chronic imipramine on mGlu4R expression in the rat brain has also been observed by another group [200]. Further studies are needed to clarify the effect of antidepressant treatments on group III mGluRs. 

Pharmacological studies of group III mGluRs have been limited due to lack of subtype-selective compounds. However, antidepressant-like effect of the group III mGluR agonist, ACPT-I, has been described in the forced-swim test [156,213,214]. Moreover, the mGluR4 positive allosteric modulator, PHCCC [214] and the selective mGluR8 agonist, RSPPG [156] induced an antidepressant-like in the forced-swim test. Moreover, mGluR7 knockout mice displayed an antidepressant-like phenotype in the forced-swim test and the tail suspension test [215]. In line with the latter study, the selective mGluR7 agonist AMN082 induced a dose-dependent decrease in the immobility time in the forced swim test and tail suspension test, supporting the hypothesis of antidepressant-like potency of mGluR7 agonists [216]. In the same study, AMN082 did not change the behaviour of mGluR7 knockout mice in the tail suspension test, whilst imipramine significantly reduced their immobility, indicating an mGluR7-dependent mechanism of the antidepressant-like activity of AMN082. ACPT-I, a group III mGluR agonist, produced anxiolytic-like effect after central administration [217] as assessed by the stress-induced hyperthermia and elevated plus-maze tests in mice, and the Vogel test in rats. The potential anxiolytic effect of ACPT-I in the stress-induced hyperthermia test was inhibited by the benzodiazepine receptor antagonist flumazenil and by the 5-HT_1A_ receptor antagonist, WAY100635. At the same time, ritanserin, a 5-HT_2A/C_ receptor antagonist, did not change the anxiolytic-like effects of ACPT-I. The results of these studies indicate that both GABAergic and serotonergic systems are involved in the potential anxiolytic action of ACPT-I [217].

Currently, little is known about the dysfunction of the mGluR8. A recent study evaluated the behaviour of mGluR8 knockout mice in different behavioural tasks commonly used in neuropsychiatric research [218]. These mice expressed no anxiogenic phenotype in unconditioned anxiety models, including elevated plus maze, elevated zero maze and light-dark box test. However, a contextual fear deficit was observed in the mGluR8 knockout mice, indicating that these receptors may be implicated in some anxiety disorders, such as generalised anxiety [219].

Despite the limited number of studies on selective group III mGluR ligands the present studies indicate that compounds belonging to this group may possess antidepressant and anxiolytic properties. 

## PARKINSON’S DISEASE

Parkinson’s disease (PD) is the second most common neurodegenerative disease affecting up to 3% of the elderly population worldwide [220,221]. It is characterized by motor symptoms such as rigidity, tremor, bradykinesia, postural instability and gait disturbances. PD is a progressive neurodegenerative disease affecting dopaminergic neurons in the substantia nigra pars compacta selectively. The resulting loss of dopaminergic innervation in the striatum is believed to be the primary event underlying the motor symptoms of PD. This loss of dopaminergic tone within the striatum leads to secondary disturbances in the two efferent striatal systems arising from the medium spiny neurons, termed the direct and indirect pathways [222]. Both pathways converge in substantia nigra pars reticulata/globus pallidus interna. However, the indirect pathway comprises projections to the globus pallidus externa, then to the subthalamic nucleus, and finally to the substantia nigra pars reticulata/globus pallidus interna. Accordingly, stimulation of these two pathways oppositely regulates the main basal ganglia output pathway, the nigrothalamic pathway. In this respect, a loss of dopamine tone in the striatum is assumed to induce an unbalance between the two pathways, namely a disinhibition of striatopallidal neurons and an inhibition of striatonigral neurons, ultimately resulting in an increased activity of the GABAergic nigrothalamic pathway [223]. It is generally believed that restoration of a normal level of activity within the indirect pathway would provide symptomatic effect in PD patients. Within this pathway, the subthalamic nucleus seems to play a critical role since it displays a continuous abnormal bursting mode of activity in PD patients [224,225]. Interestingly, similar findings have also been reported in rodent models based on the use of a neurotoxin, e.g. 6-hydroxydopamine (6-OHDA) [226]. In the clinic, long-term use of L-DOPA, the most prescribed anti-Parkinsonian drug, often results in a loss of efficacy and the apparition of dyskinesias [227]. It is thus essential to develop novel pharmacotherapies exhibiting a sustained symptomatic effect in the advanced stage of the disease, but also the potential to delay or stop the progression of the disease in its early stage. Since several subtypes of mGluRs are expressed at relevant synapses along the indirect pathway, they may represent a promising strategy for the treatment of PD and have been suggested to provide symptomatic as well as neuroprotective potential (Table **5**).

### Selective Group I Modulation

mGluR1 and mGluR5 are located postsynaptically to glutamatergic terminals in almost all striatal medium spiny neurons, as well as in the globus pallidus, subthalamic nucleus and substantia nigra reticulate [228], and are therefore in key positions to modulate neuronal activity within the indirect pathway. Activation of group I mGluRs has been shown to enhance NMDA currents in striatal neurons [229], as well as induce overactivity of the striatopallidal pathway as measured by proenkephalin mRNA expression [230]. Based on these and other evidence, it was hypothesized that blockade of group I mGluRs might induce antiparkinsonian-like effects *in vivo*. In line with this assumption, it was reported that intra-striatal blockade of mGluR1/5 by AIDA, reversed haloperidol-induced catalepsy in rats, an effect attributed to normalization of the activity of striatopallidal neurons [231]. Several studies have used subtype-selective antagonists of group I mGluRs to further dissect out the contribution of mGluR1 and mGluR5 to a potential antiparkinsonian-like effect in relevant animal models. For instance, intrapallidal injections of the selective mGluR1 agonist DHPG have been shown to reduce amphetamine-induced rotational behavior in 6-OHDA-lesioned rats [232]. In addition, the selective mGluR1 antagonist EMQMCM, was shown to slightly inhibit haloperidol-induced catalepsy, while it was devoid of effect on hypoactivity induced by a lower dose of haloperidol [233]. Similar findings were reported with the selective mGluR5 antagonist MTEP. In the same study, both EMQMCM and MTEP were ineffective to induce rotations in unilateral DA-depleted rats, further weakening a potential symptomatic benefit of group I antagonism. Interestingly, an antiparkinsonian-like profile has been reported for another non-competitive mGluR5 antagonist, MPEP [234]. However, it was shown that chronic, but not acute treatment with MPEP, reversed akinesia in a reaction time test in bilateral 6-OHDA-lesioned rats. Furthermore, acute treatment with MPEP did not induce rotations in unilateral 6-OHDA-lesioned rats, finding in agreement with the study by Dekundy *et al.* [233]. In contrast, chronic administration of MPEP induced a significant increase in the number of rotations in unilateral 6-OHDA-lesioned rats. In a more recent study, it was shown that the increased metabolic activity in the subthalamic nucleus of dopamine-depleted rats, as detected by cytochrome oxidase staining, was reversed in animals chronically treated with MPEP [235]. Taken together, these data indicate that mGluR5 blockade would normalize the hyperactive state of the subthalamic nucleus, most likely indirectly through modulation of striatopallidal neurons [236], thereby leading to antiparkinsonian-like effects. In line with this assumption, local injections of MPEP into the subthalamic nucleus attenuated motor asymmetries in unilateral 6-OHDA-lesioned rats [237]. In summary, mGluR5 antagonism may offer symptomatic improvement based on the reported antiparkinsonian-like effects in animal models, whereas mGluR1 antagonism holds less promise.

Besides a potential symptomatic effect, group I mGluR antagonism has been suggested to be of potential interest with respect to alleviation of L-DOPA-induced dyskinesias. Whereas selective mGluR1 antagonists such as EMQMCM or AIDA were ineffective in a rodent model of L-DOPA-induced dyskinesias [233], several preclinical studies have suggested such a potential for mGluR5 blockade. Dyskinesias induced by an acute challenge of L-DOPA after three weeks of priming with L-DOPA were prevented by acute administration, as well as by chronic co-administration of MTEP in 6-OHDA-lesioned rats [233,238]. These data suggest that mGluR5 antagonism can prevent priming of dyskinesias, as well as reverse the expression of dyskinesias in primed animals, an effect attributed to the normalization of an excessive GABA overflow in the substantia nigra reticulata [239]. Furthermore, it was reported in an early study that another mGluR5 antagonist, SIB-1893, alleviated dyskinesia induced by L-DOPA without changing its therapeutic effect in a primate model of PD [240]. Further supporting a role of mGluR5 in L-DOPA-induced dyskinesias, it was recently reported that mGluR5 binding was increased in the putamen and pallidum of dyskinetic MPTP-treated primates, while mGluR5 binding was normalized when dyskinesias were prevented by NMDA receptor blockade [241]. Taken together, these findings strongly suggest a potential benefit of mGluR5 antagonism in the alleviation of L-DOPA-induced dyskinesias typically observed after long-term treatment in patients. Furthermore, the preclinical findings also indicate that the symptomatic effect of L-DOPA would not be affected when considering mGluR5 antagonism as an adjunct therapy to L-DOPA.

In addition to putative symptomatic and antidyskinetic effects of mGluR5 blockade relevant in the late stage of Parkinson’s disease, a neuroprotective potential relevant in the early stage of the disease has also been suggested. Preclinical studies have shown that repeated intranigral injection of either LY367385, a mGluR1 antagonist or MPEP, a mGluR5 antagonist, produced robust neuroprotection of nigral dopaminergic neurons in 6-OHDA-lesioned rats [242]. Interestingly, subchronic intranigral injections with LY367385 or MPEP were also reported to slow down dopamine cell loss in rats already undergoing nigrostriatal degeneration, a more clinically relevant model [243], indicative of a potential rescuing effect of group I mGluR antagonism. One of the caveats in the latter studies is related to the fact that MPEP also acts as a NMDA receptor antagonist depending on the concentration used, which may account for the observed neuroprotective effect. Glutamate excitotoxicity has thus been hypothesized to contribute to the progression of dopamine neurodegeneration in Parkinson’s disease, and would occur as a consequence of the hyperactive state of glutamatergic neurons in the subthalamic nucleus [244,245]. However, it was reported that MPEP lost its neuroprotective properties against MPTP toxicity in mGluR5 knockout mice, ruling out a possible contribution of NMDA receptors [246]. Taken together, preclinical findings indicate that mGluR5, and possibly mGluR1 antagonism, may offer neuroprotective benefit slowing down the progression of the disease in its early stages. 

### Selective Group II Modulation

The mGluR2/3 are expressed presynaptically on glutamatergic and GABAergic terminals in several nuclei of the basal ganglia, including striatum, globus pallidus and substantia nigra [16,16], where their activation acts to reduce synaptic neurotransmitter release. Interestingly, an adaptive down-regulation of mGluR2/3 in Parkinsonian patients has been reported and suggested to compensate for increased glutamatergic transmission [247]. In preclinical studies, several group II mGluR agonists have been shown to reduce motor abnormalities observed in animal models of Parkinson’s disease. For instance, intraventricular administration of DCG-IV reversed reserpine-induced akinesia in rats [248]. In addition, another group II mGluR agonist, LY354740, was reported to reverse haloperidol-induced catalepsy in rats, an effect attributed at least partly to mGluR2/3-mediated reduction in glutamate release from subthalamic terminals [249]. However, contrasting results were obtained with LY379268, a selective mGluR2/3 agonist, which failed to reverse reserpine-induced akinesia and failed to affect rotational behaviour in 6-OHDA-lesioned rats [250]. In neuroprotective studies, LY379268 provided some protection against 6-OHDA neurotoxicity in rats, an effect correlated to both functional improvement and correction of dopamine turnover [250], as well as against MPTP neurotoxicity in mice as assessed by reduction of the extent of nigrostriatal degeneration [251]. In agreement with the assumption that glutamate excitotoxicity contributes to dopamine cell loss, mGluR2/3 may exert neuroprotective effects *via *regulation of glutamatergic transmission at the subthalamonigral synapse onto dopaminergic neurons [252]. 

In summary, activation of mGluR2/3 may offer symptomatic as well as neuroprotective benefit in the treatment of Parkinson’s disease. However, more studies would be needed especially with respect to motor improvement as contradictory data have been reported in the literature. 

### Selective Group III Modulation

Group III mGluRs are presynaptically localized on GABAergic and glutamatergic terminals in several basal ganglia nuclei, including globus pallidus and substantia nigra, and therefore represent potential targets for reduction of abnormal activity associated with PD [253]. Several preclinical studies support a possible therapeutic benefit of mGluR4 agonism, while only few studies have investigated the role of other subtypes, e.g. mGluR7 and mGluR8.

#### mGluR4

Positive modulation of mGluR4 has recently gained interest as a potential strategy aimed at improving motor symptoms in both early and late stages of Parkinson’s disease, as well as offering neuroprotection in early stages. Early preclinical studies have shown that the selective group III agonist L-AP4 produced symptomatic improvement in both acute and chronic rodent models of Parkinson's disease, including haloperidol-induced catalepsy, reserpine-induced akinesia, and forelimb asymmetry in unilateral 6-OHDA-lesioned rats [254]. It was further reported in the same study that L-AP4 modulated glutamatergic transmission at the striato-pallidal synapse through activation of mGluR4, as indicated by the loss of effect in brain slices from mGluR4 knockout mice. This mechanism was also suggested to contribute to the observed behavioral improvements. In line with this hypothesis, intrapallidal administration of L-AP4 was later reported to alleviate 6-OHDA-induced akinesia assessed in a reaction time task in rats [255]. A selective allosteric potentiator of mGluR4, PHCCC, was also reported to reverse reserpine-induced akinesia in rats, an effect again attributed to the modulation of striatopallidal glutamate transmission [256]. Recently, a novel mGluR4-preferring agonist, LSP1-2111, was shown to reverse akinesia assessed by a reaction time task in 6-OHDA-lesioned rats after intrapallidal injection, and block haloperidol-induced catalepsy after systemic administration [257]. Furthermore, LSP1-2111 reduced glutamate transmission at the striatopallidal sysnapse by a presynaptic mechanism, as shown for L-AP4. Taken together, a symptomatic potential of mGluR4 agonism is strongly supported by preclinical studies in animal models of Parkinson’s disease. 

In neuroprotection studies, both acute and subchronic intranigral injections of L-AP4 are found to prevent dopaminergic cell loss induced by 6-OHDA in rats [242,243]. Subchronic intranigral injections of L-AP4 were further shown to slow down dopaminergic neurodegeneration in 6-OHDA-lesioned rats already undergoing degeneration [243]. In addition, L-AP4 was also reported to protect cultured dopaminergic neurons against rotenone toxicity [258]. Based on the glutamate excitotoxicity hypothesis of dopaminergic degeneration, mGluR4 activation on glutamatergic terminals of subthalamonigral neurons have been suggested to contribute to such a neuroprotective effect. In fact, L-AP4 was shown to inhibit excitatory transmission in the substantia nigra pars compacta, and the effect was potentiated by PHCCC, further involving a selective effect of mGluR4 [259]. These studies suggest that a decrease in excitatory glutamatergic transmission from the subthalamic nucleus may contribute to a possible neuroprotective benefit of mGluR4 positive modulation. 

In summary, preclinical evidence support a putative symptomatic and neuroprotective benefit of mGluR4 positive modulation in the treatment of Parkinson’s disease. However, more selective and brain penetrant compounds would be needed to strengthen this assumption.

#### mGluR7

Recently, the selective allosteric mGluR7 agonist, AMN082, was shown to reverse haloperidol-induced catalepsy in rats after intrastriatal or systemic administration [260]. The same anticataleptic effect of AMN082 was also found in wild-type, but not in mGluR7 knockout mice, indicative of a selective mGluR7-mediated effect. In addition, AMN082 was found to reverse apomorphine-induced rotations in unilateral 6-OHDA-lesioned rats as well as reverse akinesia in a reaction time task in bilateral 6-OHDA-lesioned rats [260]. This unique study suggests that mGluR7 positive modulation may represent an interesting strategy with respect to alleviation of motor symptoms in Parkinson’s disease. Additional studies are needed to address whether a neuroprotective potential can also be achieved.

## FRAGILE X SYNDROME

Fragile X syndrome (FXS) is the most common form of inherited mental retardation and is characterized by intellectual disabilities, autistic features, hyperactivity, audiogenic seizures, and certain physical features, e.g. elongation of the face, enlargement of the ears and postpubertal macroorchidism [261,262]. It is usually caused by a mutation of the fragile X mental retardation-1 gene (FMR1), leading to either decreased levels or complete loss of the FMR1 gene product fragile X mental retardation protein (FMRP) [263,264]. FMRP is an RNA binding protein that controls for instance synthesis of certain components of the postsynaptic density (PSD) in both the neocortex and hippocampus, as well as translational efficiency of dendritic mRNAs in response to stimulation of mGluRs [265-271]. 

Fmr1 knockout mice display physical and behavioural phenotypes comparable to those of the human counterpart, such as macroorchidism, increased locomotor activity, audiogenic seizures, and impaired fear-conditioned memory [272-274]. Similarly to post-mortem observations in FXS patients [275-277], Frm1 knockout mice showed altered morphology of cortical and hippocampal dendritic spines, e.g. more spines are prolonged and immature, [276,278]. These observation suggest that FMRP plays a role in spine development and stabilisation [279], and a loss of FRMP function may result in impaired synaptic activity underlying mental retardation [280]. 

### Selective Group I Modulation

Bear *et al.* (2004) proposed that group I mGluRs might be involved in FXS. More precisely, FXS would involve an increased occurrence of a non-NMDA receptor-dependent form of long-term depression (LTD) induced by activation of the group I mGluRs [267,281]. This mGluR-LTD is an activity-dependent synaptic weakening, at least partly mediated by group I mGluRs [280,282]. In line with this hypothesis, it has been demonstrated that Fmr1 knockout mice display an increased occurrence of mGluR-LTD [281], while NMDA receptor-LTD has been found to be normal in the Fmr1 knockout mice in the hippocampus and cerebellum [61,281]. Furthermore, activation of group I mGluRs by DHPG has been shown to induce mGluR-LTD at excitatory synapses onto CA1 pyramidal cells [281,283,284]. It is believed that activation of group I mGluRs initiates postsynaptic protein synthesis, while FMRP suppresses the translation of the mRNA encoding proteins involved in mGluR-LTD, thus leading to functionally opposite roles of group I mGluRs and FMRP. It has therefore been hypothesized that FXS symptoms would result from an aberrant group I mGluR-mediated protein synthesis, in turn leading to mGluR-LTD involving internalization of AMPA receptors, GluR1 and GluR2, at dendritic synapses [264]. Since both LTD and long-term potentiation (LTP) are believed to contribute to learning and memory [281], an increased mGluR-LTD may interfere with the establishment and maintenance of strong synapses required for memory formation. 

Recently, heterozygotes double Fmr1/Grm5 (encoding mGluR5) knockout mice and have been generated [285]. Interestingly, these mice showed no difference from wild type mice with regard to spine density, thus suggesting that a 50% decrease of group I mGluR signalling is sufficient to rescue the increased spine density phenotype in the Fmr1 knockout. In this and other examples, it was demonstrated that heterozygote Fmr1/Grm5 knockout mice were devoid of the phenotypes, except macroorchidism, characterizing the Fmr1 knockout mice. These data suggest that decreased group I mGluR signalling may be a promising target for FXS.

In recent years, pharmacological studies using mGluR5 antagonists have been performed in animal models of FXS. It was demonstrated that the non-competitive antagonist MPEP increased inhibitory phosphorylation of glycogen synthase kinase-3 (GSK3) in Fmr1 knockout mice, but not in wild type mice [286]. Interestingly, it was reported that Fmr1 knockout mice display impaired inhibitory serine-phosphorylation of GSK3, and inhibition of GSK-3 by lithium ameliorated behavioural deficits in models of FXS. Taken together, these studies indicate that increased mGluR signalling in Fmr1 knockout mice may contribute to the deficit in inhibitory control of GSK3. In line with this hypothesis, studies in primary cortical neurons have shown that FMRP acted as a repressor of the translation of Shank1 mRNAs, which controls dendritic spine morphology, and that DHPG-mediated mGluR stimulation enhanced the translation of Shank1 [263].

Furthermore, McBride *et al.* (2005) showed that MPEP restored mushroom bodies, memory deficits and courtship behaviour activity in a Drosophila FXS model [287]. Courtship behaviour activity was recovered in larvae and adults treated with MPEP, suggesting that the observed effects did not result from the prevention of developmental defects. Even though the doses of MPEP used have been demonstrated to antagonize mammalian NMDA receptor activity, other competitive mGluR5 antagonists such as MPPG, MTPG, and LY341495, have shown similar rescue of the mutant flies, suggesting that the observed effects result from mGluR5 blockade. In a follow up study, it was shown that learning deficits, which appeared at an older age than the deficits in training and memory, were rescued by the same mGluR antagonists as used previously [288]. Interestingly, the learning deficits were rescued with treatment during development alone. However, the loss of mushroom bodies could not be restored in older flies. In a zebrafish model of FXS, MPEP has been shown to either completely or partially rescue the phenotypes, including craniofacial and neurite branching abnormalities [289]. In Fmr1 knockout mice, MPEP has been demonstrated to rescue heightened audiogenic seizures susceptibility, abnormal center-field behaviour [267], and impaired pre-pulse inhibition [290]. In addition, both MPEP and fenobam rescued the protrusion morphology observed in hippocampal neurons of Fmr1 knockout mice [267]. 

The mGluR5 antagonist, fenobam, has been demonstrated to reduce hyperactivity and anxiety in patients suffering from FXS in a small clinical trial [291]. Furthermore, 50% of the treated patients showed improvement in prepulse inhibition. However, this effect was not always well-correlated with subjective clinical improvement nor with the pharmacokinetics of fenobam, which showed great inter-individual differences. In preclinical studies, fenobam and other mGluR5 antagonists, including MTEP, have been reported to produce impairments in various cognitive tests, including water maze and passive avoidance tests [292,293], while other studies have not found any effect of fenobam on working memory or spatial learning at therapeutic relevant doses [183]. In line with a potential effect on cognitive function, MPEP has been demonstrated to suppress theta and gamma oscillations and impair LTP in the dentate gyrus, whereas enhanced LTP was observed in the CA1 region of rats [294]. Currently, no preclinical or clinical evidence supporting a role of selective group II or II modulators in the treatment of FXS has been published.

Since the Fmr1 gene has been identified as an autism-related gene and the most common cause of autism [295], modulation of mGluRs for the treatment of FXS might also be beneficial for autism, which is also supported by the observation that MPEP blocked repetitive features in a mice model of autism [296]. 

In conclusion, reduction in mGluR5 signalling may represent a promising target for treating many of the aspects of FXS. However, possible adverse effects of mGluR5 antagonism on cognitive function remain to be further adressed.

## HUNTINGTON’S DISEASE

Huntington’s Disease (HD) is an autosomal dominant neurodegenerative disorder characterized by involuntary body movements, cognitive deficits, and changes in personality [297-299]. Symptoms generally start appearing between mid thirties and middle age, and patients usually die 15 to 20 years after the symptomatic onset [297,299]. The neurodegeneration occurs preferentially in the striatum, extends at later stages to other brain regions including the deep layers of cortex, globus pallidus, thalamus, subthalamic nuclei, substantia nigra and gliosis formation appears [297]. In the striatum, the neuronal loss selectively affects GABAergic medium spiny neurons, whereas large aspiny cholinergic neurons are spared [297,299].

As for fragile X syndrome, HD is caused by a mutation in the gene encoding the protein huntingtin (Htt) [297,299]. Even though Htt is expressed throughout the nervous system, the neurodegeneration remains limited to specific brain areas even at late stages of HD. Some studies have suggested that Htt is required for cell survival, and loss of its function may therefore be involved in neurodegeneration [298]. Glutamate excitotoxicity has been proposed to play a central role in the pathogenesis of HD [300], and the involvement of the NMDA receptors in glutamate-mediated excitotoxicity has been especially investigated [301]. However, there is emerging evidence that mGluRs may also play a role in glutamate-mediated excitotoxicity [302]. More precisely, the identification of proteins interacting with both Htt and mGluR signalling indicates that Htt might indeed be involved in the regulation of mGluR signaling [303,304].

Pharmacological studies using a combined group I mGluR antagonist and group II mGluR agonist, (*S*)-4C3HPG, have shown a reduction in the lesion volume induced by intra-striatal quinolinate *in vivo* [305]. In contrast, no effect on lesion volume was observed with (+)-MCPG, a less potent group I/II mGluR antagonist. These data suggest that a reduction in mGluR signalling may have neuroprotective effects. Further characterization of the precise receptor subtypes involved is unfortunately lacking. 

R6/2 HD transgenic mice, which express part of the mutated human HD gene, have been reported to develop a progressive neurological phenotype with reduction in brain weight, formation of neuronal intranuclear inclusions (NII) and have limited survival [306]. They also display decreased expression of several neurotransmitter receptors including striatal mGluR1, mGluR2 and mGluR3. The decrease in the mGluR2 has been suggested to contribute to glutamate-mediated excitotoxicity by increasing glutamate release from corticostriatal terminals [307]. Decreased levels of glial glutamate transporters, which are necessary for the clearance of glutamate from the synaptic cleft, have also been reported in R6/2 HD mice, and may also contribute to excitotoxicity [308]. Schiefer *et al.* (2004) investigated the effect of the non-competitive mGluR5 antagonist, MPEP, and the mGluR2/3 agonist, LY379268, on the disease course in R6/2 HD transgenic mice. Chronic administration of both MPEP and LY379268 from a presymptomatic stage mildly, but significantly, increased survival time and reduced early hyperactivity, effects attributed to the inhibition of glutamate neurotransmission in the basal ganglia circuitry [309]. In the same study, MPEP was also found to attenuate the progressive loss of motor coordination, a robust measure of disease progression in R6/2 HD transgenic mice, while only a trend towards attenuation was observed with LY379268. These data suggest that decreasing glutamatergic transmission through modulation of mGluRs may provide symptomatic relief as well as slow down the progression of the disease. In line with a putative glutamatergic dysfunction in HD, the non-selective glutamate receptor antagonist, riluzole, which is used in the treatment of amyotrophic lateral sclerosis, has been demonstrated to increase survival time in R6/2 HD transgenic mice [310]. However, riluzole has since been demonstrated to lack efficacy in HD patients [311], which may be due to non-specific effects on other transmitter systems. 

In conclusion, mGluRs modulation may provide therapeutic benefits in HD. However, more studies aimed at elucidating the precise molecular mechanisms underlying the disease would be needed to further support a role of specific mGluR subtypes. 

## ALZHEIMER’S DISEASE

Alzheimer’s disease (AD) is characterized by the deposition of β-amyloid (Aβ) into senile plaques, the formation of neurofibrillary tangles, and neuronal death [312]. Glutamatergic pathways have been implicated in the pathophysiology of AD [313]. It has been suggested that Aβ triggers neurodegeneration by a complex interaction of processes, including increased levels of extracellular glutamate and intracellular calcium, leading to apoptosis and neuronal death [314]. 

A down-regulated and desensitized group I mGluR/ phosholipase C signalling has been demonstrated in the frontal cortex of AD patients, which was found to correlate with disease progression in the cerebral cortex [315]. Furthermore, a reduction of mGluR1 has been found in the frontal cortex of patients with AD, and this reduction was correlated with the progression of the disease [315]. These findings suggest that group I mGluR dysfunction may be involved in AD. Activation of group I mGluRs has been shown to accelerate processing of amyloid precursor protein by α-secretase into non-amylogenic products, leading to protection against Aβ deposit *in vitro* [316,317]. Thus, decreased levels and activity of group I mGluR in cerebral cortex may affect the deposition of Aβ in AD patients.

Pharmacological activation of Group II and III mGluR has been shown to reduce neuronal death *in vitro*, an effect attributed to reduced glutamate release [318,319]. Moreover, the group II mGluR agonist LY379268, was shown to protect neurons from hippocampus against excitotoxicity* in vivo* [320]. mGluR2 has been suggested to play a role in the pathogenesis of neuronal cell death and survival, as indicated by an upregulation of its expression in the hippocampus of AD patients, and a close association with hyperphosphorylated tau deposition [318,319,321]. Thus, mGluR2 might play a key role in the pathogenesis of AD [321]. Interestingly, agonists of mGluRs induced Tau phosphorylation, and several lines of evidence suggest that the differential expression of glutamate receptors in specific populations of neurons may account for specific neuronal vulnerability [314].

In conclusion, there is limited information about the role of mGluRs in the pathogenesis of AD, but it is likely that mGluRs may play a significant role in the pathophysiology of this disease. However, there is a lack of published reports, showing efficacy with mGluR ligands in AD models.

## PAIN 

Inflammatory and neuropathic pain is a major health problem affecting up to 5% of the population worldwide. The mechanisms underlying pain remain unclarified. However, it is thought that continuous activation of peripheral afferent fibers by noxious stimulation results in sensitization of dorsal horn neurons, which would subsequently produce aberrant activity in primary afferent fibers [322]. As a result, peripheral and central mechanisms contribute to a cycle of persistent nociception. Persistent activation of peripheral afferents may result in central changes in neurotransmitter release or receptor states, resulting in chronic nociceptive activation. Glutamate is released in the spinal dorsal horn, in which it acts *via *activation of ionotropic glutamate receptors as well as mGluRs [1,322]. Increasing evidence supports a specific role of mGluRs in nociceptive transmission, given the wide expression of these receptors along the nociceptive neuroaxis, such as the dorsal root ganglia, midbrain periaqueductal grey region, spinal cord, thalamus and amygdala [323]. For instance, Group I mGluRs are expressed both at the spinal and supra-spinal levels, including the thalamus, a brain area critically involved in the signaling of nociceptive information [324]. Moreover, Group I and II mGluRs are expressed by peripheral terminals and in the soma of dorsal root ganglia neurons [323]. Almost all mGluR subtypes are expressed in the spinal cord [10,12,16,17,323,325]. The mGluR3, 5, 7 and 8 have been reported to be present in the midbrain periaqueductal grey region [323], an important center for the processing of nociceptive information, and some reports suggest that group I and group III mGluRs are expressed in the amygdala [326,327], a region involved in emotional pain (Table **6**). Besides being expressed in neurons, mGluRs are also found in glial cells, which may play a role in neuropathic pain [328], but this topic will not be reviewed here. 

### Selective Group I Modulation

#### mGluR1

Early studies have reported that intrathecal injection of the non-selective mGluR1/5 agonist DHPG induced hyperalgesia and spontaneous pain in rats [329-332]. Other studies inactivating the mGluR1 by a selective antibody have shown that intrathecal blockade of mGluR1 reduced nociceptive behaviors in several models of inflammatory and neuropathic pain, including complete Freund's adjuvant (CFA)-induced chronic inflammatory pain, formalin-induced persistent nociception, nerve injury-induced neuropathic pain and DHPG-induced spontaneous nociceptive behaviours in rodents [333-337]. In line with an involvement of mGluR1 in pain mechanisms, up-regulation of mGluR1 was also demonstrated in spinal dorsal horn in response to persistent inflammatory hyperalgesia [338]. In addition to a role of spinal mGluR1 in nociception, a role of thalamic mGluR1 is also suggested by electrophysiological studies showing that the selective mGluR1 antagonist LY367385, was able to reduce the response of somatosensory neurons of the rat thalamus to noxious stimuli [339]. Several mGluR1 antagonists, e.g. CPCCOEt, AIDA, LY456236 and LY367385, have shown antinociceptive effects in various pain models such as thermal noxious, formalin-induced pain, CFA, mechanical allodynia [32,340-346]. In addition, systemic administration of the non-competitive mGluR1 antagonist, A841720, has been reported to reverse inflammatory and neuropathic pain in rodents, further supporting a therapeutic potential of mGluR1 antagonism in the treatment of chronic pain states [347,348]. Several other non-competitive mGluR1 antagonists, such as A841720, A794282, A794278 and A850002, have also been reported to attenuate spontaneous nociception in a pre-clinical model of postoperative pain [349]. Beside these pharmacological evidences, antisense knockdown of mGluR1 receptors has been reported to decrease spinal nociceptive neurotransmission and neuropathic hyperalgesia [334,336,337], further strengthening the antinociceptive potential of mGluR1 blockade.

#### mGluR5

A large body of literature supports the assumption that mGluR5 modulates pain and that mGluR5 antagonism may be used in the treatment of chronic pain conditions. As described for mGluR1, mGluR5 are also expressed both at the spinal and supra-spinal levels where they control nociceptive transmission. At the supra-spinal level, pharmacological blockade of mGluR1 by MPEP prevented the nociceptive response of sensory neurons in the rat thalamus [350]. At the behavioural level, the selective mGluR5 antagonist, SIB1757, fully reversed hyperalgesia in a neuropathic pain model in rats [351]. In addition, two other mGluR5 antagonists, MPEP and MTEP, have been shown to produce antinociceptive effects in a wide range of rat nociceptive assays, including CFA-induced chronic inflammatory pain, hyperalgesia induced by formalin and mechanical allodynia following spinal nerve ligation [32,34,343,346,352]. MPEP was also reported to prevent the increased nociceptive reaction induced by the cannabinoid receptor agonist WIN 55,212-2 in rats [353]. Moreover, MPEP abolished acetic acid-induced writhing activity in mice, and was shown to reduce mechanical allodynia and thermal hyperalgesia in a model of post-operative hypersensitivity [352]. Taken together, preclinical studies support the concept of mGluR5 antagonism for the treatment of chronic pain.

### Selective Group II Modulation

N-acetylaspartylglutamate (NAAG) is an endogenous peptide agonist that activates mGluR2/3, with preference for mGluR3 [354]. Systemic administration of NAAG or of NAAG peptidase inhibitors, ZJ-43 and 2-PMPA, have been proven to be effective in reducing perception of inflammatory and neuropathic pain in rat models [355,356]. These findings are consistent with the hypothesis that NAAG may play a physiologic role in moderation of pain perception, and that pharmacologically increasing the level of NAAG activation of mGluR2/3 may be promising in pain management. Furthermore, several studies have suggested that activation of mGluR2/3 have analgesic properties when applied centrally, or peripherally at the inflammatory site [357-363]. For instance, selective activation of peripheral mGluR2/3 with APDC was shown to reduce inflammation-induced thermal and mechanical hypersensitivity as well as formalin-induced hyperalgesia, and to contribute to the recovery from mechanical hypersensitivity following carrageenan-induced inflammation [361]. Three other mGlu2/3 agonists LY354740, LY379268 and LY389795 were also found to attenuate formalin-induced paw licking behavior [363,364] as well as carrageenan-induced thermal hyperalgesia, whereas mechanical allodynia was not affected [365].

### Selective Group III Modulation

The role of group III mGluRs in nociceptive processing has not been thoroughly investigated, but some evidence may indicate a therapeutic potential of certain subtypes. It was indeed reported that activation of group III mGluRs with l-AP4 attenuated allodynia in spinal nerve-ligated rats, but did not affect pain threshold in normal rats [366]. In addition, intrathecal injection of another group III mGluRs agonist, ACPT-I, inhibited the nociceptive responses to formalin as well as the mechanical hyperalgesia associated with inflammatory pain in carrageenan-treated and monoarthritic rats or neuropathic pain in mononeuropathic and vincristine-treated rats, while it did not affect pain threshold to mechanical or thermal stimuli in normal rats [367]. Similar antinociceptive effects were also observed following intrathecal injection of PHCCC, a mGluR4 positive allosteric modulator [367]. At the cellular level, activation of the group III mGluRs by L-AP4 reduced pain-related synaptic plasticity in the amygdala under normal conditions as well as in a model of arthritis pain [368]. Recently, Palazzo *et al.* (2008) investigated the role of mGluR7 and mGluR8 in the amygdala in pain related behaviours [369]. In their study, activation of mGluR7 by the selective agonist AMN082 was found to have pro-nociceptive effects under normal conditions, but not in the arthritic pain model, while activation of mGluR8 by the selective receptor agonist S-3,4-DCPG exhibited antinociceptive effects in arthritic, but not in normal rats [369]. Taken together, evidence supports a role of group III mGluRs in chronic pain. However, more studies using subtype selective compounds would be needed in order to identify which of group III mGluRs would be most promising.

In conclusion, mGluRs modulation may represent a promising strategy for the treatment of different types of pain, including inflammatory and neuropathic pain. At present, there are very few effective and well-tolerated therapies for neuropathic pain. Current medications include opioids, antidepressants and anticonvulsants. Modulation of mGluRs may offer better efficacy and more importantly better side-effect profile than current therapies, since the use of opioids in neuropathic pain remains controversial, and both antidepressants and anticonvulsants are associated with side-effects.

## EPILEPSY

Glutamate is widely implicated in the mechanisms underlying epilepsy [370,371]. Therefore, targets that potentially control glutamatergic transmission are of special interest to investigate as candidates to prevent epileptogenesis. In rodent models of epilepsy changes in glutamate receptor or glutamate transporter expression were shown to affect epileptic seizures [372]. Independent of the primary cause, synaptically released glutamate appears to play a major role in the initiation and spread of seizure activity. Antagonists of ionotropic glutamate receptors reduced seizures in several animals models of epilepsy [373-375]. However, these drugs failed early in clinical trials due to multiple side effects, including motor and cognitive impairment [376,377]. With the more recent discovery of mGluRs, there is a renewed interest in targeting glutamate receptors for the treatment of epilepsy. Several preclinical studies have suggested a role for mGluRs in epileptogenesis and neuronal injury [378,379] and support pharmacological modulation of specific subtypes as a potential therapeutic strategy for the treatment of epilepsy (Table **7**). 

### Selective Group I Modulation

Activation of group I mGluRs enhances neuronal excitability leading to a potentiation of NMDA and AMPA/KA receptor functions. Long-lasting functional enhancement of group I mGluR activity has been reported in amygdala-kindled rats, and up-regulation of mGluR5 immunoreactivity has been described in temporal lobe epilepsy and in focal cortical dysplasia patients [380]. Group I mGluR agonists, such as (*1S,3R*)-ACPD and 3,5-DHPG, were reported to induce limbic seizures and neuronal injury in rats [381], while other non-selective mGluR1/5 antagonists such as LY393053, LY339764, LY367335, LY367366, and LY339840 have demonstrated potent anticonvulsant activity in models of DHPG-induced limbic seizures [341]. In addition, the selective mGluR1 agonist (*S*)-4CPG reduced audiogenic seizures in DBA/2 mice [382], and attenuated pentylenetetrazol (PTZ)- and DMCM-induced seizures in rats [383]. Further supporting mGluR1 involvement in epilepsy, the mGluR1-preferring antagonists LY367385 and AIDA, blocked spike-wave discharge (SWD) in lethargic mice (*lh/lh*) [384], a genetic model of absence epilepsy [385], and reduced sound-induced clonic seizures in DBA/2 mice [384]. Moreover, these two compounds were also shown to reduce seizures in genetically epilepsy prone rats (GEPR) [341,385] and attenuate PTZ- [386,387] and DHPG- [341,388] induced seizures in rodents. AIDA further attenuated KA-induced seizures in immature rats [389] as well as kindling-related learning deficits [386]. A non-competitive mGluR1 antagonist, BAY36-7620, was reported to reduce sound-induced clonic seizures in DBA/2 mice and to attenuate PTZ- induced seizures in animals [3], further supporting an anticonvulsant effect of mGluR1 antagonism.

Two non-competitive mGluR5 antagonists, MPEP and SIB1893, were reported to suppress seizures induced by the selective mGluR5 agonist, CHPG [390]. These compounds also reduced sound-induced clonic seizures and DHPG-induced seizures in DBA/2 mice [390]. At the cellular level, MPEP suppressed DHPG-induced neuronal firing in rats, and decreased the incidence of SWD in lethargic mice (*lh/lh*) [390].

### Selective Group II modulation

Supporting a role of mGluR2/3 in epilepsy, altered expression and function of these receptors has been reported in pilocarpine-treated rats [391,392]. Following evidence that (*S*)-4-C3HPG exhibited anticonvulsant activity, compounds with preferential action on group II mGluRs have been tested in several models of epilepsy. The mGluR2 agonist, (*1S,3S*)-APDC, reduced sound-induced seizures in GEPR and DBA/2 mice, as well as DMCM-induced seizures in rats, and enhanced the generalised seizure threshold in kindled rats [383,393,394]. Activation of mGluR2 by (*1S,3S*)-APDC or DCG-IV was also found to enhance the generalised seizure threshold in kindled rats [393]. In contrast, DCG-IV was ineffective against KA-induced seizures [395]. Further supporting an anticonvulsant effect of group II mGluRs agonism, LY379268 and LY389795 reduced SWD activity in *lh*/*lh *mice [396]. 

### Selective Group III Modulation

The lack of optimal subtype selective compounds has hampered the investigation of the role of individual group III mGluRs in epileptogenesis. However, knockout animals have implicated an important role for mGluR7 in seizure activity [397,398], and a down-regulation of mGluR8 has been described in pilocarpine-epileptic rats [399]. In addition, the use of various mouse strains with differential susceptibility to pilocarpine-induced epilepsy has indicated that mGluR4 expression levels in the dentate gyrus of the hippocampus were inversely correlated with seizure susceptibility [400]. Pharmacological studies using activation of group III mGluR have yielded mixed results in animal models of epilepsy. Early studies using two group III mGluR agonists, L-AP4 and L-SOP, indicated a pro-convulsant effect of these compounds [401]. However, subsequent studies have described anticonvulsant effects of group III mGluR agonists. For instance, the group III mGluR agonist ACPT-1, with affinity for mGluR4, mGluR6 and mGluR8, reduced sound-induced seizures in GEPR and DBA/2 mice, as well as DHPG-induced seizures in rats. These data suggest that non-specific effects may be responsible for the convulsant action of L-SOP and L-AP4 [402]. Further supporting an anticonvulsant effect of group III mGluR agonism, (*R,S*)-PPG, an mGluR8-preferring agonist, was found to reduce sound-induced seizures in DBA/2 mice and GEPR, as well as electroshock-induced seizures in mice, with little evidence of any excitatory or pro-convulsant actions [402-404]. Moreover, the selective agonist for subtype 8 of group III mGluR, (*S*)-3,4-DCPG, was reported to reduce DL-homocysteic acid-induced seizures in immature rats and suppress generalized clonic-tonic seizures. Cortical energy metabolite changes associated with clonic-tonic seizures were also either normalized measured as a decrease of glucose and glycogen or markedly reduced measured as a an accumulation of lactate [405].

In summary, mGluRs modulation has shown antiepileptic activity in various animal models of generalized seizures, including sound-induced clonic seizures in DBA/2 mice, a model sensitive to all drugs clinically effective in primary generalised seizures. Suppression of SWD has been shown with some mGluR modulators in lethargic mice, a model predictive of clinical efficacy in absence seizures [406]. Spontaneous seizures following status epilepticus induced by KA or pilocarpine in rats are regarded as models of human temporal lobe epilepsy. In these models, the effects of mGluR modulators have not been thoroughly investigated and mixed results have been observed. In conclusion, the existing preclinical data positively support the therapeutic potential of mGluR ligands in epilepsy. However, studies using subtype specific agents in different models are still required.

## CONCLUSION AND PERSPECTIVES

Earlier attempts to target ionotropic glutamate receptors for the treatment of central nervous system disorders have failed due to severe side effects that included, among others, cognitive and motor impairments. As outlined in this review, targeting glutamatergic neurotransmission through modulation of the mGluR family of receptors holds great promise for the treatment of a number of central nervous system disorders, with the advantage of potential fewer side effects. From the collection of evidence presented here, one can extract that the therapeutic effects of drugs targeting mGluRs involve, in the majority of cases, a reduction in the excitatory drive either through antagonism of Group I mGluRs or activation of Group II and III mGluRs. One clear exception is the use of mGluR5 positive modulators for the treatment of cognitive deficits associated with schizophrenia. Obviously, the final result will depend on whether the targeted glutamatergic pathways are directly or indirectly involved in the pathological condition of interest, and the clinical efficacy may reside in an indirect potentiation of GABAergic, dopaminergic or other neurotransmitter systems. In addition, targeting mGluRs may also have therapeutic actions independent of glutamatergic signalling altogether, since non-glutamatergic terminals also express mGluRs.

In recent years, an intense effort has been concentrated in the synthesis and characterization of novel, more selective drugs acting on selective subtypes of mGluRs. However, whereas the advantages of some compounds resides in their selectivity, in some cases a mixed Group I antagonism/Group II or III agonism may be the key for their effectiveness, and novel therapeutic approaches may benefit from the combined selective targeting of multiple mGluRs. Many of the drugs discussed here have shown promising performance in preclinical studies. However, there is still limited clinical evidence supporting the putative therapeutic benefit of mGluR modulation in the treatment of psychiatric and neurological disorders, with the exception of a positive clinical trial of the mGluR2/3 agonist, LY2140023, for the treatment of schizophrenia [113]. Several clinical trials of subtype-selective mGluR modulators are ongoing for the treatment of schizophrenia, anxiety and depression among others. The outcome of these clinical studies will be met with great interest, since they will reveal whether targeting subtypes of mGluRs is a viable stragegy for the treatment of various central nervous system disorders both in terms of therapeutic benefits as well as side-effect liability. 

## Figures and Tables

**Fig. (1):**
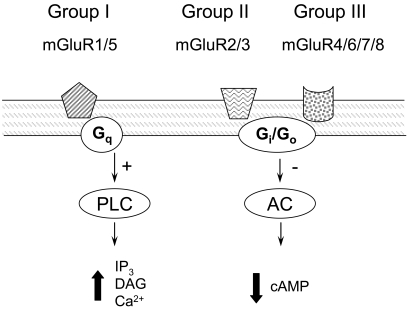
Intracellular signalling pathways associated to the different mGluR subtypes. Group I mGluRs, including mGluR1 and mGluR5, are positively coupled to phospholipase C (PLC) through activation of a G-protein of the G_q_ type; in turn, production of innositol triphosphate (IP3), release of Ca^2+^ from intracellular stores and production of diacylglycerol (DAG) activate protein kinase C. Group II and Group III mGluRs are negatively coupled to adenylate cyclase (AC) through G-protein of the G_i_/G_o_ type, leading to decreased formation of cyclic AMP (cAMP).

**Fig. (2):**
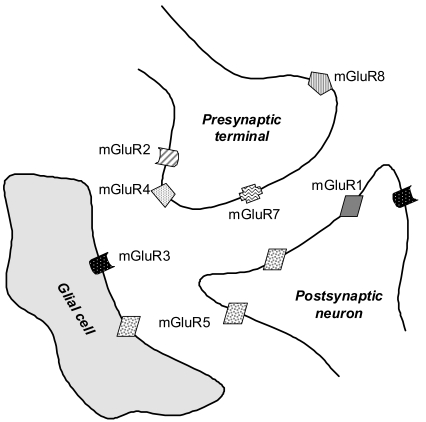
Subcellular localization of the different mGluR subtypes in the glutamatergic synapse. Group I mGluRs, including mGluR1 and mGluR5, are almost exclusively located post-synaptically, and mGluR5 are present both synaptically and extrasynaptically. Group II mGluRs are predominantly located on presynaptic terminals in the extrasynaptic space, with the exception of mGluR3 which is highly expressed on glial cells as well as postsynaptically. Group III mGluRs are also principally expressed presynaptically; interestingly, mGluR7 is located in the synaptic cleft while mGluR4 and 8 are mostly extrasynaptic. Note that mGluR3 and mGluR7 are also present on non-glutamatergic (e.g. GABAergic) terminals.

**Table 1.** Compounds with mGluR Affinity Cited in the Text with their Chemical Names, Main Target and Action

Compound Abbreviation	Chemical Name	Target	Action
A794278	9-dimethylamino-3-cycloheptyl-3H-5-thia-1,3,6-triazafluoren-4-one	Group I, mGluR1	Antagonist
A794282	9-dimethylamino-3-(4-ethylphenyl)-3H-5-thia-1,3,6-triazafluoren-4-one	Group I, mGluR1	Antagonist
A841720	9-dimethylamino-3-(N-hexamethyleneiminyl)-3H-5-thia-1,3,6-triazafluoren-4-one	Group I, mGluR1	Antagonist
A850002	9-dimethylamino-3-(4-methylphenyl)-3H-5-thia-1,3,7-triazafluoren-4-one	Group I, mGluR1	Antagonist
ACPT-I	(1S,3R,4S)-1-aminocyclopentane-1,3,4-tricarboxylic acid	Group III	Agonist
ADX47273	(S)-(4-fluorophenyl)-(3-[3-(4-fluoro-phenyl)-[1,2,4]-oxadiazol-5-yl]piperidin-1-yl) methanone	Group I, mGluR5	PAM
AIDA	1-aminoindan-1,5-dicarboxylic acid	Group I, mGluR1	Antagonist
AMN082	N,N'-dibenzhydrylethane-1,2-diamine dihydrochloride	Group III, mGluR7	Agonist
APDC	(2R,4R)-4-aminopyrrolidine-2,4-dicarboxylate	Group II	Agonist
(1S,3S)-APDC	aminopyrrolidine-2,4-dicarboxylate	Group II	Agonist
BAY367620	[(3aS,6aS)-6a-naphtalen-2-yl-methyl-5-methyliden-hexahydro-cyclopental[c]furan-1-on]	Group I, mGluR1	Antagonist
BINA	biphenyl-indanone A	Group II, mGluR2	PAM
Compound 5	1-(2-Hydroxy-3-methyl-4-{4-[4-(2H-tetrazol-5-yl)phenoxy]butoxy}phenyl)ethanone	Group II, mGluR2	PAM
Compound 8q	3-cyano-N-(1,3-diphenyl-1H-pyrazol-5-yl)benzamide	Group I, mGluR5	NAM
CDPPB	3-cyano-N-(1,3-diphenyl-1H-pyrazol-5-yl)benzamide	Group I, mGluR5	PAM
(S)-4C3HPG	4-[(1S)-1-amino-2-hydroxy-2-oxoethyl]-2-hydroxybenzoic acid	Group I; Group II	Antagonist; Agonist
CPCCOEt	7-(hydroxyimino)cyclopropa[b]chromen-1a-carboxylate ethyl ester	Group I, mGluR1	Antagonist
(S)-4CPG	(S)-4-carboxyphenylglycine	Group I	Antagonist
DCG-IV	(2S,2'R,3'R)-2-(2',3'-dicarboxycyclopropyl)glycine	Group II	Agonist
(S)-3,4-DCPG	(S)-3,4-dicarboxyphenylglycine	Group III, mGluR8	Agonist
DHPG	(S)-3,5-dihydroxyphenylglycine	Group I	Agonist
EMQMCM	3-ethyl-2-methyl-quinolin-6-yl)-(4-methoxy-cyclohexyl)-methanone methanesulfonate	Group I, mGluR1	Antagonist
Fenobam	1-(3-chlorophenyl)-3-(3-methyl-5-oxo-4H-imidazol-2-yl) urea	Group I, mGluR5	Antagonist
JNJ16259685	3,4-dihydro-2H-pyrano[2,3-b]quinolin-7-yl)-(cis-4-methoxycyclohexyl) methadone	Group I, mGluR1	Antagonist
L-AP4	L-(+)-2-amino-4-phosphonobutyric acid	Group III	Agonist
L-SOP	L-serine-O-phosphate	Group III	Agonist
LSP1-2111	Undisclosed	Group III	Agonist
LY339764	(R.S)-2-amino-2-(4-carboxycyclobutyl-3-(9-xanthen-9-yl)propanoic acid	Group I, mGluR5	Antagonist
LY339840	(S)-(+)-a-amino-4-carboxy-2-methylbenzeneacetic acid	Group I, mGluR1	Antagonist
LY341495	1S,2S)-2-[(2S)-2-amino-3-(2,6-dioxo-3H-purin-9-yl)-1-hydroxy-1-oxopropan-2-yl]cyclopropane-1-carboxylic acid	Group II	Antagonist
LY354740	(1S,2S,5R,6S)-2-aminobicyclo[3.1.0]hexane-2,6-dicarboxylic acid	Group II	Agonist
LY367335	2-amino-2-(3-cis and trans-carboxycyclobutyl)-3(9H-xanthen-9-yl)propionic acid	Group I	Antagonist
LY367366	(R,S)-2-amino-2-(4-carboxyphenyl)-3-(9H-thioxanthen-9-yl) propanoic acid	Group I, mGluR5	Antagonist
LY367385	(+)-2-methyl-4-carboxyphenylglycine	Group I, mGluR1	Antagonist
LY379268	(2R,6R)-2-amino-4-oxabicyclo[3.1.0]hexane-2,6-dicarboxylic acid	Group II	Agonist
LY389795	1R,4R,5S,6R)-4-amino-2-thiabicyclo[3.1.0]hexane-4,6-dicarboxylic acid	Group II	Agonist
LY393053	2-amino-2(S)-(3-cis-carboxycyclobutyl-3-(9-thioxanthen-9-yl)propionic acid)	Group I	Antagonist
LY404039	(-)-(1R,4S,5S,6S)-4-amino-2-sulfonylbicyclo[3.1.0]hexane-4,6-dicarboxylic acid	Group II	Agonist
LY456066	2-[4-(In-dan-2-ylamino)-5,6,7,8-tetrahydro-quinazolin-2-ylsulfanyl]-ethanol	Group I, mGluR5	Antagonist
LY456236	4-methoxy-phenyl)-(6-methoxy-quinazolin-4-yl)-amine	Group I, mGluR1	Antagonist
LY487379	2,2,2-trifluoro-N-[4-(2-methoxyphenoxy)phenyl]-N-(3-pyr idinylmethyl)ethanesulfonamide	Group II, mGluR2	PAM
MAP4	α-methyl-aminophosphonobutyrate	Group III	Antagonist
(+)-MCPG	4-[(2S)-2-amino-1-hydroxy-1-oxopropan-2-yl]benzoic acid	Group I and II	Antagonist
MGS00391	R,2R,3R,5R,6R)-2-amino-3-(3,4-dichlorobenzyloxy)-6 fluorobicyclo [3.1.0]hexane-2,6-dicarboxylic acid	Group II	Antagonist
MPEP	2-methyl-6-(2-phenylethynyl)pyridine	Group I, mGluR5	Antagonist
MPPG	2-amino-2-(4-phosphonophenyl)propanoic acid	Group II	Antagonist
MPTP	1-methyl-4-phenyl-1,2,3,6-tetrahydropyridine	Group I, mGluR5	Antagonist
MTEP	3-((2-methyl-1,3-thiazol-4-yl)ethynyl)pyridine hydrochloride	Group I, mGluR5	Antagonist
MTPG	2-amino-2-[4-(tetrazol-1-yl)phenyl]propanoic acid	Group II	Antagonist
NAAG	N-acetylaspartylglutamate	Group II,mGluR3	Agonist
PHCCC	N-phenyl-7-(hydroxyimino)cyclopropa[b]chromen-1ac-arbox amide	Group II, mGluR4	PAM
(R,S)-PPG	(RS)-4-phosphonophenylglycine	Group III	Agonist
R214127	1-(3,4-dihydro-2H-pyrano[2,3-b]quinolin-7-yl)-2-phenyl-1-ethanone	Group I, mGluR1	NAM
SIB1757	6-methyl-2-(phenylazo)-3-pyridinol	Group I, mGluR1	Antagonist
SIB1893	(E)-2-methyl-6-styrylpyridine	Group I, mGluR5	Antagonist

Abbreviations and symbols: PAM, positive allosteric modulator; NAM, negative allosteric modulator.

**Table 2.** Summary of Preclinical Studies Supporting a Role of mGluRs in Schizophrenia

Receptor Subtype	Pharmacological Tool	Effect	References

**Group I**			

mGluR1	EMQMCM (antagonist)	↔ MK-801-induced locomotor hyperactivity in rats;	Pietraszek *et al*. 2005
		↔ MK-801-induced deficits in prepulse inhibition in rats	

mGluR5	MPEP (antagonist)	↔ MK-801-induced locomotor hyperactivity in rats; ↑ MK-801-induced impairments of spatial working memory and instrumental learning in rats	Homayoun *et al*. 2004

		↓ social interaction in rats	Koros *et al*. 2007

		↓ burst firing activity in PFC neurons in awake rats;	Homayoun and Moghaddam 2006
		↑ excitatory effect of MK-801 on PFC neurons	

	Compound 8q (PAM)	↓ disruption of prepulse inhibition induced by amphetamine in rats	Lindsley *et al*. 2004

	ADX47273 (PAM)	↓ conditioned avoidance responding in rats	Liu *et al*. 2008
		↓ PCP- and amphetamine-induced locomotor hyperactivity in rats	
		↓ dopamine levels in the nucleus accumbens	
		↑ recall after a 48h delay in a novel object recognition task	

	CDPPB (PAM)	↓ set-shifting impairment induced by NMDA receptor blockade in rats	Darrah *et al*. 2008

		↓ bursting in PFC neurons in awake rats; ↑ excitatory effect of MK-801 on PFC neurons	Lecourtier *et al*. 2007

**Group II**			

mGluR2	Compound 5 (PAM)	↓ ketamine-induced hyperactivity in rats	Pinkerton *et al*. 2005

	BINA (PAM)	↓ PCP-, but not amphetamine-induced hyperactivity in mice; ↓ PCP-induced disruption in sensorimotor gating in mice	Galici *et al*. 2006

	LY487379 (PAM)	↓ neonatal PCP-induced deficits in social discrimination in rats	Harich *et al*. 2007

mGluR2/3	LY354740 (agonist)	↓ PCP-induced glutamate efflux in the nucleus accumbens and prefrontal cortex; ↓ PCPinduced locomotor hyperactivity in rats; ↔ dopamine levels in nucleus accumbens	Moghaddam and Adams 1998

		↓ PCP-induced deficits in delayed alternation task in rats; ↓ neonatal PCP-induced deficits in social discrimination in rats	Harich *et al*. 2007

		↓ performances in a delayed alternation task in rats; ↔ PCP-induced deficits in delayed alternation task in rats	Schlumberger *et al*. 2009

		↓ firing rate and ↑ burst firing of pyramidal cells in the prefrontal cortex in awake rats; reverses ↑ firing rate and ↓ burst firing induced by MK-801	Homayoun *et al*. 2005

	LY404039 (agonist)	↓ PCP-induced and amphetamine-induced locomotor hyperactivity;	Rorick-Kehn *et al*. 2007
		↓ conditioned avoidance responding in rats	

		↓ PCP-induced and amphetamine-induced locomotor hyperactivity abolished in mGluR2, but not mGluR3 knockout mice	Fell *et al*. 2008

**Group III**			

mGluR7	AMN082 (agonist)	Prevents ↓ EPSC frequency induced by L-AP4 in dopaminergic neurons	de Rover *et al*. 2008

		↔ basal or cocaine-induced increase in dopamine levels in the nucleus accumbens; ↔ basal or cocaine-induced locomotor hyperactivity in rats; ↓ cocaine-induced decrease in GABA levels in the ventral pallidum	Li *et al*. 2009

mGluR8	(S)-3,4-DCPG (agonist)	↓ amphetamine-induced locomotor hyperactivity in mice when administered i.c.v.; trend to ↓ spontaneous locomotor activity	Robbins *et al*. 2007

Abbreviations and symbols: ↔, unchanged; ↑, increased; ↓, decreased; MK-801, dizocilpine maleate; PAM, positive allosteric modulator; PFC, prefrontal cortex; PCP, phencyclidine; EPSC, excitatory postsynaptic currents; i.c.v., intracerebroventricular injection.

**Table 3.** Summary of Preclinical Studies Supporting a Role of mGluRs in Addiction

Receptor Subtype	Pharmacological Tool	Effect	References
** Group I**			
mGluR1	CPCCPOEt (antagonist)	↓ Ethanol reward, ethanol-induced CPP and ethanol consumption in mice	Lominac *et al.* 2006
	EMQMCM (antagonist)	↓ Expression of cocaine sensitization	Dravolina *et al.* 2006; Kotlinska and Bochenski 2009
		↓ Cue-induced and nicotine priming-induced reinstatement of nicotine-seeking behaviour in rats	Dravolina *et al.* 2006
		↓ Expression of sensitization to morphine	Kotlinska and Bochenski 2007
	JNJ16259685 (antagonist)	↓ Drug context-induced reinstatement of cocaine-seeking behaviour in rats when infused intra-hippocampus	Xie *et al.* 2010
mGluR5	MPEP (antagonist)	↓ Ethanol reward, ethanol-induced CPP and ethanol consumption in mice	Lominac *et al.* 2006
		↓ Cue-induced reinstatement of alcohol-seeking behaviour in alcohol-preferring rats	Schroeder *et al.* 2008
		↓ Expression of sensitization to nicotine and nicotine-induced drug-seeking behaviour in rats	Tessari *et al.* 2004
		↓ Cocaine self-administration cocaine-induced reinstatement of drug seeking in squirrel monkeys	Lee *et al.* 2005; Platt *et al.* 2008
		↓ Expression of cocaine sensitization in rats	Tessari *et al.* 2004
	MTEP (antagonist)	↔ Expression of cocaine sensitization in rats	Dravolina *et al.* 2006
		↓ Reinforcing effects of methamphetamine and cue- and drug-induced reinstatement of methamphetamine-seeking behaviour in rats	Gass *et al.* 2009
		↓ Expression of morphine sensitization and naloxone-induced symptoms of morphine withdrawal in morphine-dependent mice	Kotlinska and Bochenski 2007;Palucha *et al.* 2004a
** Group II**			
mGluR2/3	LY354740 (agonist)	↓ Naloxone-induced symptoms of morphine withdrawal in morphine-dependent mice	Klodzinska *et al.* 1999)
	LY379268 (agonist)	↓ Ethanol self-administration and cue-induced reinstatement of ethanol seeking in rats; ↓ locomotor activity at same doses	Bäckström and Hyytiä 2005
		↓ Drug-induced reinstatement of cocaine seeking in rats	Peters and Kalivas 2006
		↓ Nicotine self-administration and drug-induced reinstatement of nicotine seeking in rats	Liechti *et al.* 2007
		↓ Stress- and drug context-induced reinstatement of ethanol seeking in rats	Zhao *et al.* 2006
		↓ Cue-induced reinstatement of heroin seeking in rats after acute treatment; tolerance observed after 14 days of treatment	Bossert *et al.* 2006
** Group III**			
mGluR7	AMN082 (agonist)	↓ Rewarding effects of cocaine	Li *et al.* 2009
mGluR8	(S)- 3,4-DCPG (agonist)	↓ Ethanol self-administration and cue-induced reinstatement of ethanol seeking; ↓ Locomotor activity at same doses	Bäckström and Hyytiä 2005

Abbreviations and symbols: ↔ unchanged; ↑, increased; ↓, decreased; CPP, conditioned place preference.

**Table 4.** Summary of Preclinical Studies Supporting a Role of mGluRs in Major Depression Disorder and Anxiety

Receptor Subtype	Pharmacological Tool	Effect	References
** Group I**			
mGluR1	JNJ16259685 (antagonist)	↓ Anxiety-like behaviour in the rat lick suppression test	Steckler *et al.* 2005
	AIDA (antagonist)	↓ Anxiogenic behaviour in the rat light-dark test and open-field test	Mikulecka and Mares 2009
mGluR5	MPEP (antagonist)	↓ Anxiogenic behaviour in elevated-plus maze, social exploration, fear-potentiated startle, Vogel-conflict and light-dark box test	Ballard *et al.* 2005; Spooren *et al.* 2000; Spooren and Gasparini 2004; Tatarczynska *et al.* 2001; Mikulecka and Mares 2009; Spanka *et al.* 2010
	MTEP (antagonist)	↓ Anxiogenic behaviour contextual fear conditioning	Gravius *et al.* 2008
	MPEP & MTEP (antagonists)	↓ Immobility time in the tail-suspension test and forced-swim test	Tatarczynska *et al.* 2001; Palucha *et al.* 2005; Belozertseva *et al.* 2007; Li * et al.* 2006
		Antidepressant-like effects in the olfactory bulbectomy model	Palucha *et al.* 2005;Pilc *et al.* 2002
** Group II**			
mGluR2/3	LY341495 (antagonist)	↑ Firing rate of serotonergic dorsal raphe neurones	Kawashima *et al.* 2005
	MGS0039 (antagonist)	↑ Firing rate of serotonergic dorsal raphe neurones	Kawashima *et al.* 2005
		↑ Extracellular levels of serotonin in the rat prefrontal cortex	Karasawa *et al.* 2005
	LY341495 & MGS0039 (antagonists)	↓ Immobility and in the tail-suspension test and forced-swim test	Witkin *et al.* 2007; Chaki *et al.* 2004
	LY341495 (antagonist)	Effective in the marble burying test ↔ Anxiogenic behaviour in elevated plus maze and stress-induced hyperthermia tests	Bespalov *et al.* 2008
	MGS0039 (antagonist)	↓ Anxiogenic behaviour in the conditioned fear model	Yoshimizu *et al.* 2006
** Group III**			
	ACPT-I (agonist)	↓ Immobility time in the forced-swim test	Tatarczynska *et al.* 2002; Palucha *et al.* 2004c; Klak *et al.* 2007
		↓ Anxiogenic behaviour in the stress-induced hyperthermia, elevated plus-maze tests and in the Vogel test	Stachowicz *et al.* 2009
mGluR4	PHCCC (PAM)	↓ Immobility time in the forced-swim test	Klak *et al.* 2007
mGluR7	AMN082 (agonist)	↓ Immobility time in the forced swim test and tail suspension test	Palucha *et al.* 2007
mGluR8	RSPPG (agonist)	↓ Immobility time in the forced-swim test	Palucha *et al.* 2004b

Abbreviations and symbols: ↔, unchanged; ↑, increased; ↓, decreased; PAM, positive allosteric modulator.

**Table 5.** Summary of Preclinical Studies Supporting a Role of mGluRs in Parkinson’s Disease

Receptor Subtype	Pharmacological Tool	Effect	References
** Group I**			
	DHPG (agonist)	↓ Amphetamine-induced rotations in 6-OHDA rats	Agari *et al.* 2008
mGluR1	EMQMCM (antagonist)	↓ Haloperidol-induced catalepsy; ↔ haloperidol-induced locomotor Hypoactivity in rats; ↔ L-DOPA-induced dyskinesias	Dekundy *et al.* 2006
	AIDA (antagonist)	↔ L-DOPA-induced dyskinesias	Dekundy *et al.* 2006
	LY367385 (antagonist)	Neuroprotection and rescue against 6-OHDA toxicity in rats	Vernon *et al.* 2005; 2007
mGluR5	MPEP (antagonist)	↓ Reaction time in partial bilaterally 6-OHDA-lesioned rats after chronic treatment	Breysse *et al.*, 2002; 2003
		Neuroprotection and rescue against 6-OHDA toxicity in rats	Vernon *et al.* 2005; 2007
		↓ Motor asymmetry in unilateral 6-OHDA-lesioned rats after STN administration	Phillips *et al.* 2006
	MTEP (antagonist)	↓ Priming and expression of L-DOPA-induced dyskinesia in rats	Rylander *et al.* 2009;Dekundy *et al.* 2006
		↓ Haloperidol induced catalepsy; ; ↔ haloperidol-induced locomotor hypoactivity in rats	Dekundy *et al.* 2006
	SIB-1893 (antagonist)	↓ L-DOPA-induced dyskinesias in MPTP primates; ↔ symptomatic effect of L-DOPA	Hill *et al.* 2001
** Group II**			
mGluR2/3	DCG-IV (agonist)	↓ Reserpine-induced akinesia in rats	Dawson *et al.* 2000
	LY354740 (agonist)	↓ Haloperidol-induced catalepsy in rats	Bradley *et al.* 2000
	LY379268 (agonist)	↔ Reserpine-induced akinesia in rats; ↔ rotations in 6-OHDA rats ↓ Nigrostriatal degeneration after 6-OHDA in rats	Murray *et al.* 2002
		↓ Nigrostriatal degeneration after MPTP in mice	Battaglia *et al.* 2003
** Group III**			
	L-AP4 (agonist)	↓ Haloperidol-induced catalepsy; ↓ reserpine-induced akinesia in rats; ↓ forelimb asymmetry in 6-OHDA-lesioned rats	Valenti *et al.* 2003
		↓ Reaction time in partial bilaterally 6-OHDA-lesioned rats	Lopez *et al.* 2007
		Neuroprotection and rescue following 6-OHDA lesion in rats	Vernon *et al.* 2005; 2007
		Neuroprotection against rotenone toxicity	Jiang *et al.* 2006
mGluR4	PHCCC	↓ Reserpine-induced akinesia in rats	Marino *et al.* 2003
	LSP1-2111 (agonist)	↓ Haloperidol induced catalepsy in rats; ↓ reaction time in partial bilaterally 6-OHDA-lesioned rats; ↓ glutamate transmission at the striatopallidal synapse	Beurrier *et al.* 2009
mGluR7	AMN082 (agonist)	↓ Haloperidol induced catalepsy; ↓ apomorphine-induced rotations in unilateral 6-OHDA rats; ↓ reaction time deficits in bilateral 6-OHDA rats	Greco *et al.* 2009

Abbreviations and symbols: ↑, increased; ↓, decreased ; ↔, unchanged; 6-OHDA, 6- hydroxydopamine; ACh, Acetylcholine; MPTP, L-3,4-dihydroxyphenylalanine; PAM, positive allosteric modulator; STN, subthalamic nucleus.

**Table 6.** Summary of Preclinical Studies Supporting a Role of mGluRs in Pain

Receptor Subtype	Pharmacological Tool	Effect	References

** Group I**			

mGluR1	A841720 (antagonist)	↓ CFA-induced pain in rats	El-Kouhen *et al.* 2006;Zheng *et al.* 2005
		↓ Mechanical allodynia in rats	

	A841720	↓ Post-operative pain in rats	Zhu *et al.* 2008
	A794282		
	A794278		
	A850002 (antagonists)		

	CPCCOEt (antagonist)	↓ Formalin- induced pain in mice	Bhave *et al.* 2001;Han and Neugebauer 2005
		↓ Thermal hyperalgesia in mice	
		↓ Noxious stimulation- induced pain in rats	

	AIDA	↓ Formalin- induced pain in mice	Lee *et al.* 2007;Varty *et al.* 2005;Zhang *et al.* 2002
	LY456236	↓ Thermal hyperalgesia in mice and rats	
	LY36738 (antagonists)	↓ Mechanical allodynia in rats	

mGluR5	SIB1757 (antagonist)	↓ Thermal hyperalgesia in rats	Dogrul *et al.* 2000

	MPEP & MTEP (antagonists)	↓ Formalin- induced pain in mice	Bhave *et al.* 2001;Varty*et al.* 2005;Zhu *et al.* 2004;Lee *et al.* 2007;Walker*et al.* 2001
		↓ Post-operative pain in rats	
		↓Thermal hyperalgesia in mice	
		↓ Noxious stimulation- induced pain in rats	
		↓ Carrageenan- induced pain in mice	
		↓ CFA- induced pain in rats	
		↓ Touch-evoked allodynia in mice	
		↓ Mechanical allodynia in mice	

** Group II**			

	LY354740	↓ Carrageenan- induced pain in rats	Simmons *et al.* 2002;Jones *et al.* 2005
	LY379268	↓ CFA-induced pain in rats	
mGluR2/3	LY389795 (agonists)	↓ Formalin-induced pain in rats	
		↓ Mechanical allodynia in rats	
		↓ Thermal hyperalgesia in rats	

	APDC (agonist)	↓ Thermal-induced pain in rats	Neugebauer and Carlton 2002;Yang and Gereau 2003
		↓ Formalin-induced pain in rats	
		↓ Carrageenan- induced pain in mice	
		↓ CFA- induced pain in rats	
		↓ Thermal hyperalgesia in rats	

** Group III**			

mGluR4/7/8	l-AP4 (agonist)	↓ Mechanical allodynia in rats	Chen and Pan 2005

	ACPT-I (agonist)	↓ Carrageenan-induced pain in rats	Goudet *et al.* 2008
		↓ Vincristine-induced pain in rats	

mGluR4	PHCCC (PAM)	↓ Carrageenan-induced pain in rats	Goudet *et al.* 2008
		↓ Vincristine-induced pain in rats	

mGluR7	AMN082 (agonist)	↓ Carrageenan-induced pain in rats	Palazzo *et al.* 2008

	(*S*)-3,4-DCPG (agonist)	↓ Carrageenan-induced pain in rats	Palazzo *et al.* 2008
		↓ Vincristine-induced pain in rats	

Abbreviations and symbols: ↓, decreased; CFA, complete Freund's adjuvant; NAM, negative allosteric modulator; PAM, positive allosteric modulator.

**Table 7.** Summary of Preclinical Studies Supporting a Role of mGluRs in Epilepsy

Receptor Subtype	Pharmacological Tool	Effect	References

**Group I**			

	LY393053	↓ Seizures induced by DHPG in mice	Kingston *et al*. 2002
	LY339764		
	LY367335		
	LY367366		
	LY339840 (antagonists)		

mGluR1	(S)-4CPG (antagonist)	↓ Seizures in DBA/2 mice	Dalby and Thomsen 1996; Thomsen *et al*. 1994
		↓ Seizures induced by PTZ and DMCM in mice	

	LY367385 (antagonist)	↓ Seizures in DBA/2 mice	Burgess *et al*. 1997; Chapman *et al*. 1999; Kingston *et al*. 2002; Nagaraja *et al*. 2004; Renaud *et al*. 2002; Smolders *et al*. 2004; Thomsen and Dalby 1998
		↓ SWD in *lh/lh* mouse	
		↓ Seizures in GEPR	
		↓ Seizures induced by DHPG	
		↓ Seizures induced by kindling	
		↔ KA-induced and pilocarpine-induced seizures in rats	

	AIDA (antagonist)	↓ Seizures in DBA/2 mice	Burgess *et al*. 1997; Chapman *et al*. 1999; Kingston *et al*. 2002; Nagaraja *et al*. 2004; Renaud *et al*. 2002; Smolders *et al*. 2004; Thomsen and Dalby 1998
		↓ SWD in lh/lh mouse	
		↓ Seizures in GEPR	
		↓ Seizures induced by kindling and KA in rats	
		↔ Pilocarpine-induced seizures in rats	

	BAY367620 (antagonist)	↓ Seizures in DBA/2 mice	De Vry *et al*. 2001
		↓ Seizures in DBA/2 mice	

mGluR5	MPEP (antagonist)	↓ Seizures in DBA/2 mice	Chapman *et al*. 2000
		↓ Seizures induced by DHPG and CHPG in mice	
		↓ SWD in *lh/lh* mouse	
		↔ KA-induced and pilocarpine-induced seizures in rats	

	SIB1893 (antagonist)	↓ Seizures induced by DHPG and CHPG in mice	Chapman *et al*. 2000
		↓ Seizures in DBA/2 mice	
		↓ SWD in *lh/lh* mouse	
		↔ Kindling-induced and pilocarpine-induced seizures in rats	

**Group II**			

mGluR2/3	(1S,3S)-APDC (agonist)	↓ Seizures in DBA/2 mice	Attwell *et al*. 1998; Dalby and Thomsen 1996
		↓ Seizures induced by DMCM	
		↔ PTZ-induced seizures in mice	
		↔ ES in mice	

mGluR2/3	DCG-IV (agonist)	↓ Seizures induced by kindling in rats	Attwell *et al*. 1998; Miyamoto *et al*. 1997
		↔ KA-induced seizures in rats	

**Group III**			

mGluR4/7/8	ACPT-1 (agonist)	↓ Seizures in DBA/2 mice	Chapman *et al*. 2001
		↓ Seizures induced by DHPG in mice	
		↓ GEPR	

mGluR4/7/8	(R,S)-PPG (agonist)	↓ Seizures in DBA/2 mice	Chapman *et al*. 1999; Gasparini *et al*. 1999
		↓ ES in mice	
		↓ Seizures in GEPR	

mGluR8	(S)-3,4-DCPG (agonist)	↓ Seizures induced by DL-HCA in rats	Folbergrova *et al*. 2008

Abbreviations and symbols: ↔ unchanged; ↓ decreased; DHPG, 3,5-dihydroxyphenylglycine; PTZ, pentylenetetrazol; DMCM, Methyl-6,7-dimethoxy-4-ethyl-beta-carboline-2-carboxylate; GEPR, Genetically epilepsy prone rats; KA, kainic acid (KA); SWD, spike-wave discharge; C3HPG, (S)-4-carboxy-3-hydroxyphenylglycine; ES, Electroshock seizure; DL-HCA, DL-homocysteic acid.

## References

[1] Conn P J, Pin J P (1997). Pharmacology and functions of metabotropic glutamate receptors. Annu. Rev. Pharmacol. Toxicol.

[2] Pin J P, Duvoisin R (1995). The metabotropic glutamate receptors: structure and functions. Neuropharmacology.

[3] De Vry J, Horvath E, Schreiber R (2001). Neuroprotective and behavioral effects of the selective metabotropic glutamate mGlu(1) receptor antagonist BAY 36-7620. Eur. J. Pharmacol.

[4] Masu M, Tanabe Y, Tsuchida K, Shigemoto R, Nakanishi  
S (1991). Sequence and expression of a metabotropic glutamate receptor. Nature.

[5] Abe T, Sugihara H, Nawa H, Shigemoto R, Mizuno N, Nakanishi S (1992). Molecular characterization of a novel metabotropic glutamate receptor mGluR5 coupled to inositol phosphate/Ca^2+^ 
signal transduction. J. Biol. Chem.

[6] Kew J N, Kemp J A (2005). Ionotropic and metabotropic glutamate receptor structure and pharmacology. Psychopharmacology (Berl).

[7] Martin L J, Blackstone C D, Huganir R L, Price D L (1992). Cellular localization of a metabotropic glutamate receptor in rat brain. Neuron.

[8] Fotuhi M, Sharp A H, Glatt C E, Hwang P M, von K M, Snyder S H, Dawson T M (1993). Differential localization of phosphoinositide-linked metabotropic glutamate receptor (mGluR1) and the inositol 1,4,5-trisphosphate receptor in rat brain. J. Neurosci.

[9] Lujan R, Nusser Z, Roberts J D, Shigemoto R, Somogyi P (1996). Perisynaptic location of metabotropic glutamate receptors mGluR1 and mGluR5 on dendrites and dendritic spines in the rat hippocampus. Eur. J. Neurosci.

[10] Shigemoto R, Nomura S, Ohishi H, Sugihara H, Nakanishi S, Mizuno N (1993). Immunohistochemical localization of a metabotropic glutamate receptor, mGluR5, in the rat brain. Neurosci. Lett.

[11] Rodrigues R J, Alfaro T M, Rebola N, Oliveira C R, Cunha R A (2005). Co-localization and functional interaction between adenosine A(2A) and metabotropic group 5 receptors in glutamatergic nerve terminals of the rat striatum. J. Neurochem.

[12] Romano C, Sesma M A, McDonald C T, O'Malley K, Van den Pol A N, Olney J W (1995). Distribution of metabotropic glutamate receptor mGluR5 immunoreactivity in rat brain. J. Comp. Neurol.

[13] Lujan R, Roberts J D, Shigemoto R, Ohishi H, Somogyi P (1997). Differential plasma membrane distribution of metabotropic glutamate receptors mGluR1 alpha, mGluR2 and mGluR5, relative to neurotransmitter release sites. J. Chem. Neuroanat.

[14] Ferraguti F, Shigemoto R (2006). Metabotropic glutamate receptors. Cell Tissue Res.

[15] Petralia R S, Wang Y X, Niedzielski A S, Wenthold R J (1996). The metabotropic glutamate receptors, mGluR2 and mGluR3, 
show unique postsynaptic, presynaptic and glial localizations. Neuroscience.

[16] Shigemoto R, Kinoshita A, Wada E, Nomura S, Ohishi H, Takada M, Flor P J, Neki A, Abe T, Nakanishi S, Mizuno N (1997). Differential Presynaptic Localization of Metabotropic Glutamate Receptor Subtypes in the Rat Hippocampus. J. Neurosci.

[17] Tamaru Y, Nomura S, Mizuno N, Shigemoto R (2001). Distribution of metabotropic glutamate receptor mGluR3 in the mouse CNS: differential location relative to pre- and postsynaptic sites. Neuroscience.

[18] Hayashi Y, Momiyama A, Takahashi T, Ohishi H, Ogawa-Meguro R, Shigemoto R, Mizuno N, Nakanishi S (1993). Role of a metabotropic glutamate receptor in synaptic modulation in the accessory olfactory bulb. Nature.

[19] Neki A, Ohishi H, Kaneko T, Shigemoto R, Nakanishi S, Mizuno N (1996). Pre- and postsynaptic localization of a metabotropic glutamate receptor, mGluR2, in the rat brain: an immunohistochemical study with a monoclonal antibody. Neurosci. Lett.

[20] Neki A, Ohishi H, Kaneko T, Shigemoto R, Nakanishi S, Mizuno N (1996). Metabotropic glutamate receptors mGluR2 and mGluR5 are expressed in two non-overlapping populations of Golgi cells in the rat cerebellum. Neuroscience.

[21] Ohishi H, Akazawa C, Shigemoto R, Nakanishi S, Mizuno N (1995). Distributions of the mRNAs for L-2-amino-4-phosphonobutyrate-sensitive metabotropic glutamate receptors, mGluR4 and mGluR7, in the rat brain. J. Comp. Neurol.

[22] Ohishi H, Ogawa-Meguro R, Shigemoto R, Kaneko T, Nakanishi S, Mizuno N (1994). Immunohistochemical localization of metabotropic glutamate receptors, mGluR2 and mGluR3, in rat cerebellar cortex. Neuron.

[23] Nakajima Y, Iwakabe H, Akazawa C, Nawa H, Shigemoto R, Mizuno N, Nakanishi S (1993). Molecular characterization of a novel retinal metabotropic glutamate receptor mGluR6 with a high agonist selectivity for L-2-amino-4-phosphonobutyrate. J. Biol. Chem.

[24] Glaum S R, Miller R J (1993). Metabotropic glutamate receptors depress afferent excitatory transmission in the rat nucleus tractus solitarii. J. Neurophysiol.

[25] Dalezios Y, Lujan R, Shigemoto R, Roberts J D, Somogyi P (2002). Enrichment of mGluR7a in the presynaptic active zones of GABAergic and non-GABAergic terminals on interneurons in the rat somatosensory cortex. Cereb. Cortex.

[26] Somogyi P, Dalezios Y, Lujan R, Roberts J D, Watanabe M, Shigemoto R (2003). High level of mGluR7 in the presynaptic active zones of select populations of GABAergic terminals innervating 
interneurons in the rat hippocampus. Eur. J. Neurosci.

[27] Azkue J J, Murga M, Fernandez-Capetillo O, Mateos J M, Elezgarai I, Benitez R, Osorio A, Diez J, Puente N, Bilbao A, Bidaurrazaga A, Kuhn R, Grandes P (2001). Immunoreactivity for the group III metabotropic glutamate receptor subtype mGluR4a 
in the superficial laminae of the rat spinal dorsal horn. J. Comp. Neurol.

[28] Jia H, Rustioni A, Valtschanoff J G (1999). Metabotropic glutamate receptors in superficial laminae of the rat dorsal horn. J. Comp. Neurol.

[29] Kinzie J M, Saugstad J A, Westbrook G L, Segerson T P (1995). Distribution of metabotropic glutamate receptor 7 messenger RNA in the developing and adult rat brain. Neuroscience.

[30] Li H, Ohishi H, Kinoshita A, Shigemoto R, Nomura S, Mizuno N (1997). Localization of a metabotropic glutamate receptor, mGluR7 in axon terminals of presumed nociceptive, primary 
afferent fibers in the superficial layers of the spinal dorsal horn: an electron microscope study in the rat. Neurosci. Lett.

[31] Ohishi H, Nomura S, Ding Y Q, Shigemoto R, Wada E, Kinoshita A, Li J L, Neki A, Nakanishi S, Mizuno N (1995). Presynaptic localization of a metabotropic glutamate receptor, mGluR7, in the primary afferent neurons: an immunohistochemical study in the rat. Neurosci. Lett.

[32] Bhave G, Karim F, Carlton S M, Gereau R W (2001). Peripheral group I metabotropic glutamate receptors modulate nociception in mice. Nat. Neurosci.

[33] Carlton S M, Hargett G L (2007). Colocalization of metabotropic 
glutamate receptors in rat dorsal root ganglion cells. J. Comp. 
Neurol.

[34] Walker K, Reeve A, Bowes M, Winter J, Wotherspoon G, Davis A, Schmid P, Gasparini F, Kuhn R, Urban L (2001). mGlu5 receptors and nociceptive function II. mGlu5 receptors functionally expressed on peripheral sensory neurones mediate inflammatory hyperalgesia. Neuropharmacology.

[35] Taylor D L, Jones F, Kubota E S, Pocock J M (2005). Stimulation of microglial metabotropic glutamate receptor mGlu2 triggers 
tumor necrosis factor alpha-induced neurotoxicity in concert 
with microglial-derived Fas ligand. J. Neurosci.

[36] Taylor D L, Diemel L T, Cuzner M L, Pocock J M (2002). Activation of group II metabotropic glutamate receptors underlies microglial reactivity and neurotoxicity following stimulation with chromogranin A, a peptide up-regulated in Alzheimer's disease. J. Neurochem.

[37] Deng W, Wang H, Rosenberg P A, Volpe J J, Jensen F E (2004). Role of metabotropic glutamate receptors in oligodendrocyte 
excitotoxicity and oxidative stress. Proc. Natl. Acad. Sci. USA.

[38] Muyderman H, Angehagen M, Sandberg M, Bjorklund U, Olsson T, Hansson E, Nilsson M (2001). Alpha 1-adrenergic modulation of metabotropic glutamate receptor-induced calcium oscillations and glutamate release in astrocytes. J. Biol. Chem.

[39] Aronica E, Gorter J A, Ijlst-Keizers H, Rozemuller A J, Yankaya B, Leenstra S, Troost D (2003). Expression and functional role of mGluR3 and mGluR5 in human astrocytes and glioma cells: opposite regulation of glutamate transporter proteins. Eur. J. Neurosci.

[40] Aronica E, Gorter J A, Rozemuller A J, Yankaya B, Troost D (2005). Activation of metabotropic glutamate receptor 3 enhances interleukin (IL)-1beta-stimulated release of IL-6 in cultured human astrocytes. Neuroscience.

[41] Glaum S R, Miller R J (1992). Metabotropic glutamate receptors mediate excitatory transmission in the nucleus of the solitary tract. J. Neurosci.

[42] Anwyl R (1999). Metabotropic glutamate receptors: electrophysiological properties and role in plasticity. Brain Res. Brain Res. Rev.

[43] Pinheiro P S, Mulle C (2008). Presynaptic glutamate receptors: physiological functions and mechanisms of action. Nat. Rev. Neurosci.

[44] Barrie A P, Nicholls D G, Sanchez-Prieto J, Sihra T S (1991). An ion channel locus for the protein kinase C potentiation of transmitter glutamate release from guinea pig cerebrocortical synaptosomes. J. Neurochem.

[45] Coffey E T, Herrero I, Sihra T S, Sanchez-Prieto J, Nicholls D G (1994). Glutamate exocytosis and MARCKS phosphorylation are enhanced by a metabotropic glutamate receptor coupled to a protein kinase C synergistically activated by diacylglycerol and arachidonic acid. J. Neurochem.

[46] Wang S J, Sihra T S (2004). Noncompetitive metabotropic glutamate5 receptor antagonist (E)-2-methyl-6-styryl-pyridine (SIB1893) depresses glutamate release through inhibition of voltage-dependent Ca^2+^ entry in rat cerebrocortical nerve terminals (synaptosomes). J. Pharmacol. Exp. Ther.

[47] Sanchez-Prieto J, Paternain A V, Lerma J (2004). Dual signaling by mGluR5a results in bi-directional modulation of N-type Ca^2+^ channels. FEBS Lett.

[48] Kreitzer A C, Regehr W G (2001). Retrograde inhibition of presynaptic calcium influx by endogenous cannabinoids at exci-tatory synapses onto Purkinje cells. Neuron.

[49] Pelkey K A, Topolnik L, Lacaille J C, McBain C J (2006). Compartmentalized Ca(2+) channel regulation at divergent mossy-fiber release sites underlies target cell-dependent plasticity. Neuron.

[50] Robbe D, Alonso G, Chaumont S, Bockaert J, Manzoni O J (2002). Role of p/q-Ca^2+^ channels in metabotropic glutamate receptor 2/3-dependent presynaptic long-term depression at nucleus accumbens synapses. J. Neurosci.

[51] O'Connor V, El F O, Bofill-Cardona E, Nanoff C, Freissmuth M, Karschin A, Airas J M, Betz H, Boehm S (1999). Calmodulin dependence of presynaptic metabotropic glutamate receptor signaling. Science.

[52] Pelkey K A, Lavezzari G, Racca C, Roche K W, McBain C J (2005). mGluR7 is a metaplastic switch controlling bidirectional plasticity of feedforward inhibition. Neuron.

[53] Perroy J, Prezeau L, De W M, Shigemoto R, Bockaert J, Fagni L (2000). Selective blockade of P/Q-type calcium channels by the metabotropic glutamate receptor type 7 involves a phospholipase C pathway in neurons. J. Neurosci.

[54] Blackmer T, Larsen E C, Takahashi M, Martin T F, Alford S, Hamm H E (2001). G protein betagamma subunit-mediated presynaptic inhibition: regulation of exocytotic fusion downstream of Ca^2+^ entry. Science.

[55] Blackmer T, Larsen E C, Bartleson C, Kowalchyk J A, Yoon E J, Preininger A M, Alford S, Hamm H E, Martin T F (2005). G protein betagamma directly regulates SNARE protein fusion machinery for secretory granule exocytosis. Nat. Neurosci.

[56] Gerachshenko T, Blackmer T, Yoon E J, Bartleson C,  
Hamm H E, Alford S (2005). Gbetagamma acts at the C terminus of SNAP-25 to mediate presynaptic inhibition. Nat. Neurosci.

[57] Delaney A J, Crane J W, Sah P (2007). Noradrenaline modulates transmission at a central synapse by a presynaptic mechanism. Neuron.

[58] Bashir Z I, Jane D E, Sunter D C, Watkins J C, Collingridge G L (1993). Metabotropic glutamate receptors contribute to the induction of long-term depression in the CA1 region of the hippocampus. Eur. J. Pharmacol.

[59] Bolshakov V Y, Siegelbaum S A (1994). Postsynaptic induction and presynaptic expression of hippocampal long-term depression. Science.

[60] Fitzjohn S M, Kingston A E, Lodge D, Collingridge G L (1999). DHPG-induced LTD in area CA1 of juvenile rat hippocampus, characterisation and sensitivity to novel mGlu receptor antagonists. Neuropharmacology.

[61] Kemp N, Bashir Z I (1999). Induction of LTD in the adult hippocampus by the synaptic activation of AMPA/kainate and metabotropic glutamate receptors. Neuropharmacology.

[62] O'Mara S M, Rowan M J, Anwyl R (1995). Metabotropic glutamate receptor-induced homosynaptic long-term depression and depotentiation in the dentate gyrus of the rat hippocampus *in vitro*. Neuropharmacology.

[63] Palmer M J, Irving A J, Seabrook G R, Jane D E, Collingridge G L (1997). The group I mGlu receptor agonist DHPG induces a novel form of LTD in the CA1 region of the hippocampus. Neuropharmacology.

[64] Wang W, Zhang Z, Shang J, Jiang Z Z, Wang S, Liu Y, Zhang L Y (2008). Activation of group I metabotropic glutamate receptors induces long-term depression in the hippocampal CA1 region of adult rats *in vitro*. Neurosci. Res.

[65] Gubellini P, Saulle E, Centonze D, Bonsi P, Pisani A, Bernardi G, Conquet F, Calabresi P (2001). Selective involvement of mGlu1 receptors in corticostriatal LTD. Neuropharmacology.

[66] Malenka R C, Bear M F (2004). LTP and LTD: an embarrassment of riches. Neuron.

[67] Kobayashi K, Manabe T, Takahashi T (1996). Presynaptic long-term depression at the hippocampal mossy fiber-CA3 synapse. Science.

[68] Yokoi M, Kobayashi K, Manabe T, Takahashi T, Sakaguchi I, Katsuura G, Shigemoto R, Ohishi H, Nomura S, Nakamura K, Nakao K, Katsuki M, Nakanishi S (1996). Impairment of hippocampal mossy fiber LTD in mice lacking mGluR2. Science.

[69] Chen Y L, Huang C C, Hsu K S (2001). Time-dependent reversal of long-term potentiation by low-frequency stimulation at the hippocampal mossy fiber-CA3 synapses. J. Neurosci.

[70] Hong I, Song B, Lee S, Kim J, Kim J, Choi S (2009). Extinction of cued fear memory involves a distinct form of depotentiation at cortical input synapses onto the lateral amygdala. Eur. J. Neurosci.

[71] Laezza F, Doherty J J, Dingledine R (1999). Long-term depression in hippocampal interneurons: joint requirement for pre- and postsynaptic events. Science.

[72] Gladding C M, Fitzjohn S M, Molnar E (2009). Metabotropic glutamate receptor-mediated long-term depression: molecular mechanisms. Pharmacol. Rev.

[73] Anwyl R (2009). Metabotropic glutamate receptor-dependent long-term potentiation. Neuropharmacology.

[74] Bashir Z I, Bortolotto Z A, Davies C H, Berretta N, Irving A J, Seal A J, Henley J M, Jane D E, Watkins J C, Collingridge G L (1993). Induction of LTP in the hippocampus needs synaptic activation of glutamate metabotropic receptors. Nature.

[75] Gubellini P, Saulle E, Centonze D, Costa C, Tropepi D, Bernardi G, Conquet F, Calabresi P (2003). Corticostriatal LTP requires combined mGluR1 and mGluR5 activation. Neuropharmacology.

[76] Rebola N, Lujan R, Cunha R A, Mulle C (2008). Adenosine A2A receptors are essential for long-term potentiation of NMDA-EPSCs at hippocampal mossy fiber synapses. Neuron.

[77] Kwon H B, Castillo P E (2008). Long-term potentiation selectively expressed by NMDA receptors at hippocampal mossy fiber 
synapses. Neuron.

[78] Bortolotto Z A, Collett V J, Conquet F, Jia Z, van der Putten H, Collingridge G L (2005). The regulation of hippocampal LTP by the molecular switch, a form of metaplasticity, requires mGlu5 receptors. Neuropharmacology.

[79] Lu Y M, Jia Z, Janus C, Henderson J T, Gerlai R,  
Wojtowicz J M, Roder J C (1997). Mice lacking metabotropic glutamate receptor 5 show impaired learning and reduced CA1 long-term potentiation (LTP) but normal CA3 LTP. J. Neurosci.

[80] Naie K, Manahan-Vaughan D (2004). Regulation by metabotropic 
glutamate receptor 5 of LTP in the dentate gyrus of freely moving rats: relevance for learning and memory formation. Cereb. Cortex.

[81] Conquet F, Bashir Z I, Davies C H, Daniel H, Ferraguti F, Bordi F, Franz-Bacon K, Reggiani A, Matarese V, Conde F (1994). Motor deficit and impairment of synaptic plasticity in mice lacking mGluR1. Nature.

[82] Little Z, Grover L M, Teyler T J (1995). Metabotropic glutamate receptor antagonist, (R,S)-alpha-methyl-4-carboxyphenyglycine, blocks two distinct forms of long-term potentiation in area CA1 of rat hippocampus. Neurosci. Lett.

[83] Wang Y, Rowan M J, Anwyl R (1997). LTP induction dependent on activation of Ni2+-sensitive voltage-gated calcium channels, but not NMDA receptors in the rat dentate gyrus *in vitro*. J. Neurophysiol.

[84] Lante F, de Jesus Ferreira M C, Guiramand J, Recasens M, Vignes M (2006). Low-frequency stimulation induces a new form of LTP metabotropic glutamate (mGlu5) receptor- and PKA-dependent, in the CA1 area of the rat hippocampus. Hippocampus.

[85] Liang Y C, Huang C C, Hsu K S (2005). Characterization of long-term potentiation of primary afferent transmission at trigeminal synapses of juvenile rats: essential role of subtype 5 metabotropic glutamate receptors. Pain.

[86] Piccinin S, Thuault S J, Doherty A J, Brown J T, Randall A D, Davies C H, Bortolotto Z A, Collingridge G L (2008). The induction of long-term plasticity of non-synaptic, synchronized ac-tivity by the activation of group I mGluRs. Neuropharmacology.

[87] Grover L M, Yan C (1999). Evidence for involvement of group II/III metabotropic glutamate receptors in NMDA receptor-independent long-term potentiation in area CA1 of rat hippocampus. J. Neurophysiol.

[88] Rush A M, Wu J, Rowan M J, Anwyl R (2002). Group I 
metabotropic glutamate receptor (mGluR)-dependent long-term 
depression mediated via p38 mitogen-activated protein kinase is inhibited by previous high-frequency stimulation and activation of mGluRs and protein kinase C in the rat dentate gyrus *in vitro*. J. Neurosci.

[89] Mueser K T, McGurk S R (2004). Schizophrenia. Lancet.

[90] Gray J A, Roth B L (2007). The pipeline and future of drug development in schizophrenia. Mol. Psychiatry.

[91] Seeman P (1987). Dopamine receptors and the dopamine hypothesis of schizophrenia. Synapse.

[92] Reynolds G P (2004). Receptor mechanisms in the treatment of schizophrenia. J. Psychopharmacol.

[93] Lewis D A, Moghaddam B (2006). Cognitive dysfunction in schizophrenia: convergence of gamma-aminobutyric acid and glutamate alterations. Arch. Neurol.

[94] Gaspar P A, Bustamante M L, Silva H, Aboitiz F (2009). Molecular mechanisms underlying glutamatergic dysfunction in schizophrenia: therapeutic implications. J. Neurochem.

[95] Krystal J H, Karper L P, Seibyl J P, Freeman G K, Delaney R, Bremner J D, Heninger G R, Bowers M B, Charney D S (1994). Subanesthetic effects of the noncompetitive NMDA antagonist, ketamine, in humans. Psychotomimetic perceptual cognitive and neuroendocrine responses. Arch. Gen. Psychiatry.

[96] Lahti A C, Weiler M A, Tamara Michaelidis B A, Parwani A, Tamminga C A (2001). Effects of ketamine in normal and schizophrenic volunteers. Neuropsychopharmacology.

[97] Gupta D S, McCullumsmith R E, Beneyto M, Haroutunian V, Davis K L, Meador-Woodruff J H (2005). Metabotropic glutamate receptor protein expression in the prefrontal cortex and striatum in schizophrenia. Synapse.

[98] Brody S A, Conquet F, Geyer M A (2003). Disruption of prepulse inhibition in mice lacking mGluR1. Eur. J. Neurosci.

[99] Pietraszek M, Gravius A, Schafer D, Weil T, Trifanova D, Danysz W (2005). mGluR5, but not mGluR1 antagonist modifies MK-801-induced locomotor activity and deficit of prepulse inhibition. Neuropharmacology.

[100] Attucci S, Carla V, Mannaioni G, Moroni F (2001). Activation of type 5 metabotropic glutamate receptors enhances NMDA responses in mice cortical wedges. Br. J. Pharmacol.

[101] Gray L, van den B M, Scarr E, Dean B, Hannan A J (2009). Clozapine reverses schizophrenia-related behaviours in the metabotropic glutamate receptor 5 knockout mouse: association with N-methyl-D-aspartic acid receptor up-regulation. Int. J. Neuropsychopharmacol.

[102] Homayoun H, Stefani M R, Adams B W, Tamagan G D, Moghaddam B (2004). Functional Interaction Between NMDA and mGlu5 Receptors: Effects on Working Memory, Instrumental Learning, Motor Behaviors, and Dopamine Release. Neuropsychopharmacology.

[103] Lindsley C W, Wisnoski D D, Leister W H, O'brien J A, Lemaire W, Williams D L, Burno M, Sur C, Kinney G G, Pettibone D J, Tiller P R, Smith S, Duggan M E, Hartman G D, Conn P J, Huff J R (2004). Discovery of positive allosteric modulators for the metabotropic glutamate receptor sub-type 5 from a series of N-(1,3-diphenyl-1H- pyrazol-5-yl)benzamides that potentiate receptor function *in vivo*. J. Med. Chem.

[104] Liu F, Grauer S, Kelley C, Navarra R, Graf R, Zhang G, Atkinson P J, Popiolek M, Wantuch C, Khawaja X, Smith D, Olsen M, Kouranova E, Lai M, Pruthi F, Pulicicchio C, Day M, Gilbert A, Pausch M H, Brandon N J, Beyer C E, Comery T A, Logue S, Rosenzweig-Lipson S, Marquis K L (2008). ADX47273 [S-(4-fluoro-phenyl)-{3-[3-(4-fluoro-phenyl)-[1,2,4]-oxadiazol-5-yl]-piper idin-1-yl}-methanone]: a novel metabotropic glutamate receptor 5-selective positive allosteric modulator 
with preclinical antipsychotic-like and procognitive activities. J. Pharmacol. Exp. Ther.

[105] Diaz-Cabiale Z, Vivo M, Del A A, O'Connor W T, Harte M K, Muller C E, Martinez E, Popoli P, Fuxe K, Ferre S (2002). Metabotropic glutamate mGlu5 receptor-mediated modulation of the ventral striopallidal GABA pathway in rats. Interactions with adenosine A(2A) and dopamine D(2) receptors. Neurosci. Lett.

[106] Darrah J M, Stefani M R, Moghaddam B (2008). Interaction of N-methyl-D-aspartate and group 5 metabotropic glutamate receptors on behavioral flexibility using a novel operant set-shift paradigm. Behav. Pharmacol.

[107] Homayoun H, Moghaddam B (2006). Bursting of prefrontal cortex neurons in awake rats is regulated by metabotropic glutamate 5 (mGlu5) receptors: rate-dependent influence and interaction with NMDA receptors. Cereb. Cortex.

[108] Lecourtier L, Homayoun H, Tamagnan G, Moghaddam B (2007). Positive allosteric modulation of metabotropic glutamate 5 (mGlu5) receptors reverses N-Methyl-D-aspartate antagonist-induced alteration of neuronal firing in prefrontal cortex. Biol. Psychiatry.

[109] Koros E, Rosenbrock H, Birk G, Weiss C, Sams-Dodd F (2007). The selective mGlu5 receptor antagonist MTEP, similar to NMDA receptor antagonists, induces social isolation in rats. Neuropsychopharmacology.

[110] Devon R S, Anderson S, Teague P W, Muir W J, Murray V, Pelosi A J, Blackwood D H, Porteous D J (2001). The genomic organisation of the metabotropic glutamate receptor subtype 5 gene, and its association with schizophrenia. Mol. Psychiatry.

[111] Ohnuma T, Augood S J, Arai H, McKenna P J, Emson P C (1998). Expression of the human excitatory amino acid transporter 2 and metabotropic glutamate receptors 3 and 5 in the prefrontal cortex from normal individuals and patients with schizophrenia. Brain. Res. Mol. Brain. Res.

[112] Battaglia G, Monn J A, Schoepp D D (1997). *In vivo* inhibition of veratridine-evoked release of striatal excitatory amino acids by the group II metabotropic glutamate receptor agonist LY354740 in rats. Neurosci. Lett.

[113] Patil S T, Zhang L, Martenyi F, Lowe S L, Jackson K A, Andreev B V, Avedisova A S, Bardenstein L M, Gurovich I Y, Morozova M A, Mosolov S N, Neznanov N G, Reznik A M, Smulevich A B, Tochilov V A, Johnson B G, Monn J A, Schoepp D D (2007). Activation of mGlu2/3 receptors as a new approach to treat schizophrenia: a randomized Phase 2 clinical trial. Nat. Med.

[114] Moghaddam B, Adams B W (1998). Reversal of phencyclidine effects by a group II metabotropic glutamate receptor agonist in rats. Science.

[115] Rorick-Kehn L M, Johnson B G, Knitowski K M, Salhoff C R, Witkin J M, Perry K W, Griffey K I, Tizzano J P, Monn J A, McKinzie D L, Schoepp D D (2007). *In vivo* pharmacological characterization of the structurally novel, potent, selective mGlu2/3 receptor agonist LY404039 in animal models of psychiatric disorders. Psychopharmacology (Berl.,).

[116] Pinkerton A B, Cube R V, Hutchinson J H, James J K, Gardner M F, Rowe B A, Schaffhauser H, Rodriguez D E, Campbell U C, Daggett L P, Vernier J M (2005). Allosteric potentiators
of the metabotropic glutamate receptor 2 (mGlu2). Part 3:
Identification and biological activity of indanone containing mGlu2
receptor potentiators. Bioorg. Med. Chem. Lett.

[117] Govek S P, Bonnefous C, Hutchinson J H, Kamenecka T, McQuiston J, Pracitto R, Zhao L X, Gardner M F, James J K, Daggett L P, Rowe B A, Schaffhauser H, Bristow L J, Campbell U C, Rodriguez D E, Vernier J M (2005). Benzazoles as allosteric potentiators of metabotropic glutamate receptor 2 (mGluR2): efficacy in an animal model for schizophrenia. Bioorg. Med. Chem. Lett.

[118] Galici R, Jones C K, Hemstapat K, Nong Y, Echemendia N G, Williams L C, de P T, Conn P J (2006). Biphenyl-indanone A, a positive allosteric modulator of the metabotropic glutamate receptor subtype 2, has antipsychotic- and anxiolytic-like effects in mice. J. Pharmacol. Exp. Ther.

[119] Fell M J, Svensson K A, Johnson B G, Schoepp D D (2008). Evidence for the role of metabotropic glutamate (mGlu)2 not
mGlu3 receptors in the preclinical antipsychotic pharmacology
of the mGlu2/3 receptor agonist (-)-(1R,4S,5S,6S)-4-amino-2-
sulfonylbicyclo[3.1.0]hexane-4,6-dicarboxylic acid (LY404039). J. Pharmacol. Exp. Ther.

[120] Woolley M L, Pemberton D J, Bate S, Corti C, Jones D N (2008). The mGlu2 but not the mGlu3 receptor mediates the actions of 
the mGluR2/3 agonist, LY379268, in mouse models predictive of antipsychotic activity. Psychopharmacology (Berl.,).

[121] Harich S, Gross G, Bespalov A (2007). Stimulation of the metabotropic glutamate 2/3 receptor attenuates social novelty discrimination deficits induced by neonatal phencyclidine treatment. Psychopharmacology (Berl.,).

[122] Schlumberger C, Schafer D, Barberi C, More L, Nagel J, Pietraszek M, Schmidt W J, Danysz W (2009). Effects of a metabotropic glutamate receptor group II agonist LY354740 in animal models of positive schizophrenia symptoms and cognition. Behav. Pharmacol.

[123] Homayoun H, Jackson M E, Moghaddam B (2005). Activation of metabotropic glutamate 2/3 receptors reverses the effects of NMDA receptor hypofunction on prefrontal cortex unit activity in awake rats. J. Neurophysiol.

[124] Krystal J H, bi-Saab W, Perry E, D'Souza D C, Liu N, Gueorguieva R, McDougall L, Hunsberger T, Belger A, Levine L, Breier A (2005). Preliminary evidence of attenuation of the disruptive effects of the NMDA glutamate receptor antagonist, ketamine on working memory by pretreatment with the group II metabotropic glutamate receptor agonist LY354740 in healthy human subjects. Psychopharmacology (Berl).

[125] Ghose S, Crook J M, Bartus C L, Sherman T G, Herman M M, Hyde T M, Kleinman J E, Akil M (2008). Metabotropic glutamate receptor 2 and 3 gene expression in the human prefrontal cortex and mesencephalon in schizophrenia. Int. J. Neurosci.

[126] Ohtsuki T, Koga M, Ishiguro H, Horiuchi Y, Arai M,  
Niizato K, Itokawa M, Inada T, Iwata N, Iritani S, Ozaki N, Kunugi H, Ujike H, Watanabe Y, Someya T, Arinami T (2008). A polymorphism of the metabotropic glutamate receptor mGluR7 (GRM7) gene is associated with schizophrenia. Schizophr. Res.

[127] Shibata H, Tani A, Chikuhara T, Kikuta R, Sakai M,  
Ninomiya H, Tashiro N, Iwata N, Ozaki N, Fukumaki Y (2009). Association study of polymorphisms in the group III metabotropic glutamate receptor genes, GRM4 and GRM7, with schizophrenia. Psychiatry Res.

[128] Dev K K, Henley J M (2006). The schizophrenic faces of PICK1. Trends Pharmacol. Sci.

[129] de Rover M, Meye F J, Ramakers G M (2008). Presynaptic 
metabotropic glutamate receptors regulate glutamatergic input to dopamine neurons in the ventral tegmental area. Neuroscience.

[130] Li X, Gardner E L, Xi Z X (2008). The metabotropic glutamate 
receptor 7 (mGluR7) allosteric agonist AMN082 modulates nucleus accumbens GABA and glutamate, but not dopamine, in rats. Neuropharmacology.

[131] Li X, Li J, Peng X Q, Spiller K, Gardner E L, Xi Z X (2009). Metabotropic glutamate receptor 7 modulates the rewarding effects of cocaine in rats: involvement of a ventral pallidal GABAergic mechanism. Neuropsychopharmacology.

[132] Callaerts-Vegh Z, Beckers T, Ball S M, Baeyens F, Callaerts P F, Cryan J F, Molnar E, D'Hooge R (2006). Concomitant deficits in working memory and fear extinction are functionally dissociated from reduced anxiety in metabotropic glutamate receptor 7-deficient mice. J. Neurosci.

[133] Hölscher C, Schmid S, Pilz P K, Sansig G, van der Putten H, Plappert C F (2004). Lack of the metabotropic glutamate receptor subtype 7 selectively impairs short-term working memory but not long-term memory. Behav. Brain Res.

[134] Hölscher C, Schmid S, Pilz P K, Sansig G, van der Putten H, Plappert C F (2005). Lack of the metabotropic glutamate receptor subtype 7 selectively modulates Theta rhythm and working 
memory. Learn. Mem.

[135] Bushell T J, Sansig G, Collett V J, van der P H,  
Collingridge G L (2002). Altered short-term synaptic plasticity in mice lacking the metabotropic glutamate receptor mGlu7. Sci. World J.

[136] Takaki H, Kikuta R, Shibata H, Ninomiya H, Tashiro N, Fukumaki Y (2004). Positive associations of polymorphisms in the metabotropic glutamate receptor type 8 gene (GRM8) with schizophrenia. Am. J. Med. Genet B. Neuropsychiatr. Genet.

[137] Robbins M J, Starr K R, Honey A, Soffin E M, Rourke C, Jones G A, Kelly F M, Strum J, Melarange R A, Harris A J, Rocheville M, Rupniak T, Murdock P R, Jones D N, Kew J N, Maycox P R (2007). Evaluation of the mGlu8 receptor as a putative therapeutic target in schizophrenia. Brain Res.

[138] Baker D A, Shen H, Kalivas P W (2002). Cystine/glutamate exchange serves as the source for extracellular glutamate: modifications 
by repeated cocaine administration. Amino Acids.

[139] Moran M M, McFarland K, Melendez R I, Kalivas P W, Seamans J K (2005). Cystine/glutamate exchange regulates metabotropic glutamate receptor presynaptic inhibition of excitatory transmission and vulnerability to cocaine seeking. J. Neurosci.

[140] Milton A L, Lee J L, Butler V J, Gardner R, Everitt B J (2008). Intra-amygdala and systemic antagonism of NMDA receptors prevents the reconsolidation of drug-associated memory and impairs subsequently both novel and previously acquired drug-seeking behaviors. J. Neurosci.

[141] Zweifel L S, Argilli E, Bonci A, Palmiter R D (2008). Role of NMDA receptors in dopamine neurons for plasticity and addictive behaviors. Neuron.

[142] Lominac K D, Kapasova Z, Hannun R A, Patterson C,  
Middaugh L D, Szumlinski K K (2006). Behavioral and neurochemical interactions between Group 1 mGluR antagonists and ethanol: 
potential insight into their anti-addictive properties. Drug Alcohol Depend.

[143] Dravolina O A, Danysz W, Bespalov A Y (2006). Effects of group I metabotropic glutamate receptor antagonists on the behavioral sensitization to motor effects of cocaine in rats. Psychopharmacology (Berl.,).

[144] Kotlinska J, Bochenski M (2009). Pretreatment with Group I 
metabotropic glutamate receptors antagonists attenuates lethality induced by acute cocaine overdose and expression of sensitization to hyperlocomotor effect of cocaine in mice. Neurotox. Res.

[145] Xie X, Ramirez D R, Lasseter H C, Fuchs R A (2010). Effects of
mGluR1 antagonism in the dorsal hippocampus on drug context-induced
reinstatement of cocaine-seeking behavior in rats. Psychopharmacology
(Berl.,).

[146] Dravolina O A, Zakharova E S, Shekunova E V, Zvartau E E, Danysz W, Bespalov A Y (2007). mGlu1 receptor blockade attenuates cue- and nicotine-induced reinstatement of extinguished 
nicotine self-administration behavior in rats. Neuropharmacology.

[147] Kotlinska J, Bochenski M (2007). Comparison of the effects of mGluR1 and mGluR5 antagonists on the expression of behavioral sensitization to the locomotor effect of morphine and the morphine withdrawal jumping in mice. Eur. J. Pharmacol.

[148] Blednov Y A, Adron H R (2008). Metabotropic glutamate receptor 5 (mGluR5) regulation of ethanol sedation, dependence and consumption: relationship to acamprosate actions. Int. J. Neuropsychopharmacol.

[149] Schroeder J P, Spanos M, Stevenson J R, Besheer J, Salling M, Hodge C W (2008). Cue-induced reinstatement of alcohol-seeking behavior is associated with increased ERK1/2 phosphorylation in specific limbic brain regions: blockade by the mGluR5 antagonist MPEP. Neuropharmacology.

[150] Tessari M, Pilla M, Andreoli M, Hutcheson D M, Heidbreder C A (2004). Antagonism at metabotropic glutamate 5 receptors inhibits nicotine- and cocaine-taking behaviours and prevents nicotine-triggered relapse to nicotine-seeking. Eur. J. Pharmacol.

[151] Lee B, Platt D M, Rowlett J K, Adewale A S, Spealman R D (2005). Attenuation of behavioral effects of cocaine by the Metabotropic Glutamate Receptor 5 Antagonist 2-Methyl-6-(phenylethynyl)-pyridine in squirrel monkeys: comparison with dizocilpine. J. Pharmacol. Exp. Ther.

[152] Platt D M, Rowlett J K, Spealman R D (2008). Attenuation of cocaine self-administration in squirrel monkeys following repeated administration of the mGluR5 antagonist MPEP: comparison with dizocilpine. Psychopharmacology (Berl).

[153] Lea P M, Faden A I (2006). Metabotropic glutamate receptor subtype 5 antagonists MPEP and MTEP. CNS Drug Rev.

[154] Pisani A, Bonsi P, Centonze D, Bernardi G, Calabresi P (2001). Functional coexpression of excitatory mGluR1 and mGluR5 on striatal cholinergic interneurons. Neuropharmacology.

[155] Gass J T, Osborne M P, Watson N L, Brown J L, Olive M F (2009). mGluR5 antagonism attenuates methamphetamine reinforcement and prevents reinstatement of methamphetamine-seeking behavior in rats. Neuropsychopharmacology;.

[156] Palucha A, Tatarczynska E, Branski P, Szewczyk B, Wieron-ska J M, Klak K, Chojnacka-Wojcik E, Nowak G, Pilc A (2004). Group III mGlu receptor agonists produce anxiolytic- and antidepressant-
like effects after central administration in rats. Neuropharmacology.

[157] Klodzinska A, Chojnacka-Wojcik E, Palucha A, Branski P, Popik P, Pilc A (1999). Potential anti-anxiety, anti-addictive effects of LY 354740 a selective group II glutamate metabotropic receptors agonist in animal models. Neuropharmacology.

[158] Bäckström P, Hyytiä P (2005). Suppression of alcohol self-administration and cue-induced reinstatement of alcohol seeking by the mGlu2/3 receptor agonist LY379268 and the mGlu8 receptor agonist (S)-3,4-DCPG. Eur. J. Pharmacol.

[159] Peters J, Kalivas P W (2006). The group II metabotropic glutamate receptor agonist, LY379268, inhibits both cocaine- and food-seeking behavior in rats. Psychopharmacology (Berl).

[160] Baptista M A, Martin-Fardon R, Weiss F (2004). Preferential effects of the metabotropic glutamate 2/3 receptor agonist LY379268 on conditioned reinstatement versus primary reinforcement: comparison between cocaine and a potent conventional reinforcer. J. Neurosci.

[161] Bossert J M, Gray S M, Lu L, Shaham Y (2006). Activation of group II metabotropic glutamate receptors in the nucleus accumbens shell attenuates context-induced relapse to heroin seeking. Neuropsychopharmacology.

[162] Liechti M E, Lhuillier L, Kaupmann K, Markou A (2007). Metabotropic glutamate 2/3 receptors in the ventral tegmental area and the nucleus accumbens shell are involved in behaviors relating to nicotine dependence. J. Neurosci.

[163] Zhao Y, Dayas C V, Aujla H, Baptista M A, Martin-Fardon R, Weiss F (2006). Activation of group II metabotropic glutamate receptors attenuates both stress and cue-induced ethanol-seeking and modulates c-fos expression in the hippocampus and amygdala. J. Neurosci.

[164] Bossert J M, Liu S Y, Lu L, Shaham Y (2004). A role of ventral tegmental area glutamate in contextual cue-induced relapse to heroin seeking. J. Neurosci.

[165] Vadasz C, Saito M, Gyetvai B M, Oros M, Szakall I, Kovacs K M, Prasad V V, Toth R (2007). Glutamate receptor metabotropic 7 is cis-regulated in the mouse brain and modulates alcohol drinking. Genomics.

[166] Nielsen D A, Ji F, Yuferov V, Ho A, Chen A, Levran O, Ott J, Kreek M J (2008). Genotype patterns that contribute to increased risk for or protection from developing heroin addiction. Mol. Psychiatry.

[167] Westenberg H G, Sandner C (2006). Tolerability and safety of fluvoxamine and other antidepressants. Int. J. Clin. Pract.

[168] Cassano P, Fava M (2004). Tolerability issues during long-term treatment with antidepressants. Ann. Clin. Psychiatry.

[169] Baune B T, Adrian I, Jacobi F (2007). Medical disorders affect health outcome and general functioning depending on comorbid major depression in the general population. J. Psychosom. Res.

[170] Kessler R C, Akiskal H S, Ames M, Birnbaum H, Greenberg P, Hirschfeld R M, Jin R, Merikangas K R, Simon G E, Wang P S (2006). Prevalence and effects of mood disorders on work
performance in a nationally representative sample of U.S. workers. Am. J. Psychiatry.

[171] Katz M M, Maas J W, Frazer A, Koslow S H, Bowden C L, Berman N, Swann A C, Stokes P E (1994). Drug-induced actions on brain neurotransmitter systems and changes in the behaviors and emotions of depressed patients. Neuropsychopharmacology.

[172] Bowden C L, Koslow S H, Hanin I, Maas J W, Davis J M, Robins E (1985). Effects of amitriptyline and imipramine on brain amine neurotransmitter metabolites in cerebrospinal fluid. Clin. Pharmacol. Ther.

[173] Akimova E, Lanzenberger R, Kasper S (2009). The serotonin-1A receptor in anxiety disorders. Biol. Psychiatry.

[174] Machado-Vieira R, Salvadore G, Luckenbaugh D A, Manji H K, Zarate  C A (2008). Rapid onset of antidepressant action: a new paradigm in the research and treatment of major depressive disorder. J. Clin. Psychiatry.

[175] Henkel V, Seemuller F, Obermeier M, Adli M, Bauer M, Mundt C, Brieger P, Laux G, Bender W, Heuser I, Zeiler J, Gaebel W, Mayr A, Moller H J, Riedel M (2009). Does early improvement triggered by antidepressants predict response/remission? - Analysis of data from a naturalistic study on a large sample of inpatients with major depression. J. Affect. Disord.

[176] Millan M J (2009). Dual- and Triple-Acting agents for treating core and Co-morbid symptoms of major depression: novel concepts, new drugs. Neurotherapeutics.

[177] aan het Rot M, Collins K A, Murrough J W, Perez A M, Reich D L, Charney D S, Mathew S J (2010). Safety and efficacy of repeated-dose intravenous ketamine for treatment-resistant depression. Biol. Psychiatry.

[178] Pilc A, Branski P, Palucha A, Tokarski K, Bijak M (1998). Antidepressant treatment influences group I of glutamate metabotropic receptors in slices from hippocampal CA1 region. Eur. J. Pharmacol.

[179] Bajkowska M, Branski P, Smialowska M, Pilc A (1999). Effect of chronic antidepressant or electroconvulsive shock treatment on mGLuR1a immunoreactivity expression in the rat hippocampus. Pol. J. Pharmacol.

[180] Smialowska M, Szewczyk B, Branski P, Wieronska J M, Palucha A, Bajkowska M, Pilc A (2002). Effect of chronic imipramine or electroconvulsive shock on the expression of mGluR1a and mGluR5a immunoreactivity in rat brain hippocampus. Neuropharmacology.

[181] Steckler T, Lavreysen H, Oliveira A M, Aerts N, Van C H, Prickaerts J, Megens A, Lesage A S (2005). Effects of mGlu1 receptor blockade on anxiety-related behaviour in the rat lick suppression test. Psychopharmacology. (Berl.,).

[182] Mikulecka A, Mares P (2009). Effects of mGluR5 and mGluR1 antagonists on anxiety-like behavior and learning in developing rats. Behav. Brain Res.

[183] Ballard T M, Woolley M L, Prinssen E, Huwyler J, Porter R, Spooren W (2005). The effect of the mGlu5 receptor antagonist MPEP in rodent tests of anxiety and cognition: a comparison. Psychopharmacology (Berl).

[184] Spooren W P, Vassout A, Neijt H C, Kuhn R, Gasparini F, Roux S, Porsolt R D, Gentsch C (2000). Anxiolytic-like effects of the prototypical metabotropic glutamate receptor 5 antagonist 2-methyl-6-(phenylethynyl)pyridine in rodents. J. Pharmacol. Exp. Ther.

[185] Spooren W, Gasparini F (2004). mGlu5 receptor antagonists: a novel class of anxiolytics?. Drug News Perspect.

[186] Tatarczynska E, Klodzinska A, Chojnacka-Wojcik E, Palucha A, Gasparini F, Kuhn R, Pilc A (2001). Potential anxiolytic- and antidepressant-like effects of MPEP a potent, selective and systemically active mGlu5 receptor antagonist. Br. J. Pharmacol.

[187] Spanka C, Glatthar R, Desrayaud S, Fendt M, Orain D, Troxler T, Vranesic I (2010). Piperidyl amides as novel, potent 
and orally active mGlu5 receptor antagonists with anxiolytic-like activity. Bioorg. Med. Chem. Lett.

[188] Gravius A, Dekundy A, Nagel J, More L, Pietraszek M, Danysz W (2008). Investigation on tolerance development to subchronic blockade of mGluR5 in models of learning anxiety and levodopa-induced dyskinesia in rats. J. Neural. Transm.

[189] George S A, Hutson P H, Stephens D N (2009). Differential effects of
MPEP and diazepam in tests of conditioned emotional response
and Pavlovian-to-instrumental transfer suggests 'anxiolytic' effects
are mediated by different mechanisms. Psychopharmacology (Berl.,).

[190] Wieronska J M, Branski P, Szewczyk B, Palucha A, Papp M, Gruca P, Moryl E, Pilc A (2001). Changes in the expression of metabotropic glutamate receptor 5 (mGluR5) in the rat hippocampus in an animal model of depression. Pol. J. Pharmacol.

[191] Palucha A, Branski P, Szewczyk B, Wieronska J M, Klak K, Pilc A (2005). Potential antidepressant-like effect of MTEP, a potent and highly selective mGluR5 antagonist. Pharmacol. Biochem. Behav.

[192] Belozertseva I V, Kos T, Popik P, Danysz W, Bespalov A Y (2007). Antidepressant-like effects of mGluR1 and mGluR5 antagonists in the rat forced swim and the mouse tail suspension tests. Eur. Neuropsychopharmacol.

[193] Li X, Need A B, Baez M, Witkin J M (2006). Metabotropic glutamate 5 receptor antagonism is associated with antidepressant-like effects in mice. J. Pharmacol. Exp. Ther.

[194] Pilc A, Klodzinska A, Branski P, Nowak G, Palucha A, Szewczyk B, Tatarczynska E, Chojnacka-Wojcik E, Wieronska J M (2002). Multiple MPEP administrations evoke anxiolytic- and antidepressant-like effects in rats. Neuropharmacology.

[195] Feyissa A M, Woolverton W L, Miguel-Hidalgo J J, Wang Z, Kyle P B, Hasler G, Stockmeier C A, Iyo A H, Karolewicz B (2010). Elevated level of metabotropic glutamate receptor 2/3 in the prefrontal cortex in major depression. Prog. Neuropsychopharmacol. Biol. Psychiatry.

[196] Tsunoka T, Kishi T, Ikeda M, Kitajima T, Yamanouchi Y, Kinoshita Y, Kawashima K, Okochi T, Okumura T, Inada T, Ozaki N, Iwata N (2009). Association analysis of Group II metabotropic glutamate receptor genes (GRM2 and GRM3) with mood disorders and fluvoxamine response in a Japanese population. Prog. Neuropsychopharmacol. Biol. Psychiatry.

[197] Egan M F, Straub R E, Goldberg T E, Yakub I, Callicott J H, Hariri A R, Mattay V S, Bertolino A, Hyde T M, Shannon-Weickert C, Akil M, Crook J, Vakkalanka R K, Balkissoon R, Gibbs R A, Kleinman J E, Weinberger D R (2004). Variation in GRM3 affects cognition, prefrontal glutamate, and risk for schizophrenia. Proc. Natl. Acad Sci. USA.

[198] Wieronska J M, Legutko B, Dudys D, Pilc A (2008). Olfactory bulbectomy and amitriptyline treatment influences mGlu receptors expression in the mouse brain hippocampus. Pharmacol. Rep.

[199] Matrisciano F, Caruso A, Orlando R, Marchiafava M, Bruno V, Battaglia G, Gruber S H, Melchiorri D, Tatarelli R, Girardi P, Mathe A A, Nicoletti F (2008). Defective group-II metaboropic glutamate receptors in the hippocampus of spontaneously depressed rats. Neuropharmacology.

[200] Matrisciano F, Storto M, Ngomba R T, Cappuccio I, Caricasole A, Scaccianoce S, Riozzi B, Melchiorri D, Nicoletti F (2002). Imipramine treatment up-regulates the expression and function of mGlu2/3 metabotropic glutamate receptors in the rat hippocampus. Neuropharmacology.

[201] Kawashima N, Karasawa J, Shimazaki T, Chaki S, Okuyama S, Yasuhara A, Nakazato A (2005). Neuropharmacological profiles of antagonists of group II metabotropic glutamate receptors. Neurosci. Lett.

[202] Karasawa J, Shimazaki T, Kawashima N, Chaki S (2005). AMPA receptor stimulation mediates the antidepressant-like effect of a group II metabotropic glutamate receptor antagonist. Brain Res.

[203] Yoshimizu T, Chaki S (2004). Increased cell proliferation in the adult mouse hippocampus following chronic administration of group II metabotropic glutamate receptor antagonist, MGS0039. Biochem Biophys. Res. Commun.

[204] Tanti A, Belzung C (2010). Open questions in current models of antidepressant action. Br. J. Pharmacol.

[205] Witkin J M, Marek G J, Johnson B G, Schoepp D D (2007). Metabotropic glutamate receptors in the control of mood disorders. CNS Neurol. Disord. Drug Targets.

[206] Chaki S, Yoshikawa R, Hirota S, Shimazaki T, Maeda M, Kawashima N, Yoshimizu T, Yasuhara A, Sakagami K, Okuyama S, Nakanishi S, Nakazato A (2004). MGS0039: a potent and selective group II metabotropic glutamate receptor antagonist with antidepressant-like activity. Neuropharmacology.

[207] Cryan J F, Valentino R J, Lucki I (2005). Assessing substrates underlying the behavioral effects of antidepressants using the modified rat forced swimming test. Neurosci. Biobehav. Rev.

[208] Morishima Y, Miyakawa T, Furuyashiki T, Tanaka Y, Mizuma H, Nakanishi S (2005). Enhanced cocaine responsiveness and impaired motor coordination in metabotropic glutamate receptor subtype 2 knockout mice. Proc. Natl. Acad Sci. USA.

[209] Matrisciano F, Scaccianoce S, Del B P, Panaccione I, Canudas A M, Battaglia G, Riozzi B, Ngomba R T, Molinaro G, Tatarelli R, Melchiorri D, Nicoletti F (2005). Metabotropic glutamate receptors and neuroadaptation to antidepressants: imipramine-induced down-regulation of beta-adrenergic receptors in mice treated with metabotropic glutamate 2/3 receptor ligands. J. Neurochem.

[210] Bespalov A Y, van Gaalen M M, Sukhotina I A, Wicke K, Mezler M, Schoemaker H, Gross G (2008). Behavioral characterization of the mGlu group II/III receptor antagonist LY-341495 in animal models of anxiety and depression. Eur. J. Pharmacol.

[211] Yoshimizu T, Shimazaki T, Ito A, Chaki S (2006). An mGluR2/3
antagonist, MGS0039, exerts antidepressant and anxiolytic effects
in behavioral models in rats. Psychopharmacology (Berl.,).

[212] Wieronska J M, Klak K, Palucha A, Branski P, Pilc A (2007). Citalopram influences mGlu7, but not mGlu4 receptors' expression in the rat brain hippocampus and cortex. Brain Res.

[213] Tatarczynska E, Palucha A, Szewczyk B, Chojnacka-Wojcik E, Wieronska J, Pilc A (2002). Anxiolytic- and antidepressant-like effects of group III metabotropic glutamate agonist (1S,3R,4S)-1-aminocyclopentane-1,3,4-tricarboxylic acid (ACPT-I) in rats. Pol. J. Pharmacol.

[214] Klak K, Palucha A, Branski P, Sowa M, Pilc A (2007). Combined administration of PHCCC, a positive allosteric modulator of mGlu4 receptors and ACPT-I, mGlu III receptor agonist evokes antidepressant-like effects in rats. Amino Acids.

[215] Cryan J F, Kelly P H, Neijt H C, Sansig G, Flor P J, van der P H (2003). Antidepressant and anxiolytic-like effects in mice lacking the group III metabotropic glutamate receptor mGluR7. Eur. J. Neurosci.

[216] Palucha A, Klak K, Branski P, van der P H, Flor P J,  
Pilc A (2007). Activation of the mGlu7 receptor elicits antidepressant-
like effects in mice. Psychopharmacology (Berl).

[217] Stachowicz K, Klodzinska A, Palucha-Poniewiera A, Schann S, Neuville P, Pilc A (2009). The group III mGlu receptor agonist ACPT-I exerts anxiolytic-like but not antidepressant-like effects mediated by the serotonergic and GABA-ergic systems. Neuropharmacology.

[218] Fendt M, Burki H, Imobersteg S, van der P H, McAllister K, Leslie J C, Shaw D, Holscher C (2010). The effect of mGlu(8) 
deficiency in animal models of psychiatric diseases. Genes Brain Behav.

[219] Grillon C (2002). Startle reactivity and anxiety disorders: aversive conditioning context and neurobiology. Biol. Psychiatry.

[220] de Lau L M, Breteler M M (2006). Epidemiology of Parkinson's disease. Lancet Neurol.

[221] Muangpaisan W, Hori H, Brayne C (2009). Systematic review of the prevalence and incidence of parkinson&rsquos disease in asia. J. Epidemiol.

[222] Albin R L, Young A B, Penney J B (1995). The functional anatomy of disorders of the basal ganglia. Trends Neurosci.

[223] Wichmann T, Delong M R (2007). Anatomy and physiology of the basal ganglia: relevance to Parkinson's disease and related disorders. Handb. Clin. Neurol.

[224] Bergman H, Wichmann T, Karmon B, Delong M R (1994). The
primate subthalamic nucleus. II. Neuronal activity in the MPTP
model of parkinsonism. J. Neurophysiol.

[225] Limousin P, Pollak P, Benazzouz A, Hoffmann D, Le Bas J F, Broussolle E, Perret J E, Benabid A L (1995). Effect of parkinsonian signs and symptoms of bilateral subthalamic nucleus stimulation. Lancet.

[226] Breit S, Bouali-Benazzouz R, Popa R C, Gasser T, Benabid A L, Benazzouz A (2007). Effects of 6-hydroxydopamine-induced severe or partial lesion of the nigrostriatal pathway on the neuronal activity of pallido-subthalamic network in the rat. Exp. Neurol.

[227] Dauer W, Przedborski S (2003). Parkinson's disease: mechanisms and models. Neuron.

[228] Rouse S T, Marino M J, Bradley S R, Awad H, Wittmann M, Conn P J (2000). Distribution and roles of metabotropic glutamate receptors in the basal ganglia motor circuit: implications for treatment of Parkinson's disease and related disorders. Pharmacol. Ther.

[229] Pisani A, Calabresi P, Centonze D, Bernardi G (1997). Enhancement of NMDA responses by group I metabotropic glutamate receptor activation in striatal neurones. Br. J. Pharmacol.

[230] Mao L, Wang J Q (2001). Selective activation of group I metabotropic glutamate receptors upregulates preprodynorphin, substance P, 
and preproenkephalin mRNA expression in rat dorsal striatum. Synapse.

[231] Ossowska K, Wardas J, Pietraszek M, Konieczny J, Wolfarth S (2003). The striopallidal pathway is involved in antiparkinsonian-like effects of the blockade of group I metabotropic glutamate receptors in rats. Neurosci. Lett.

[232] Agari T, Yasuhara T, Matsui T, Kuramoto S, Kondo A, Miyoshi Y, Shingo T, Borlongan C V, Date I (2008). Intrapallidal metabotropic glutamate receptor activation in a rat model of Parkinson's disease: Behavioral and histological analyses. Brain Research.

[233] Dekundy A, Pietraszek M, Schaefer D, Cenci M A, Danysz W (2006). Effects of group I metabotropic glutamate receptors blockade in experimental models of Parkinson's disease. Brain Res. Bull.

[234] Breysse N, Baunez C, Spooren W, Gasparini F, Amalric M (2002). Chronic but not acute treatment with a metabotropic glutamate 5 receptor antagonist reverses the akinetic deficits in a rat model of parkinsonism. J. Neurosci.

[235] Breysse N, Amalric M, Salin P (2003). Metabotropic Glutamate 5 Receptor Blockade Alleviates Akinesia by Normalizing Activity 
of Selective Basal-Ganglia Structures in Parkinsonian Rats. J. Neurosci.

[236] Pisani A, Gubellini P, Bonsi P, Conquet F, Picconi B,  
Centonze D, Bernardi G, Calabresi P (2001). Metabotropic glutamate receptor 5 mediates the potentiation of N-methyl--aspartate 
responses in medium spiny striatal neurons. Neuroscience.

[237] Phillips J M, Lam H A, Ackerson L C, Maidment N T (2006). Blockade of mGluR glutamate receptors in the subthalamic nucleus ameliorates motor asymmetry in an animal model of Parkinson's disease. Eur. J. Neurosci.

[238] Rylander D, Recchia A, Mela F, Dekundy A, Danysz W, Cenci M A (2009). Pharmacological modulation of glutamate transmission in a rat model of L-DOPA-induced dyskinesia: effects on motor behavior and striatal nuclear signaling. J. Pharmacol. Exp. Ther.

[239] Mela F, Marti M, Dekundy A, Danysz W, Morari M,  
Cenci M A (2007). Antagonism of metabotropic glutamate receptor type 5 attenuates l-DOPA-induced dyskinesia and its molecular and 
neurochemical correlates in a rat model of Parkinson's disease. J. Neurochem.

[240] Hill M P, McGuire S G, Crossman A R, Brotchie J M (2001). The mGlu5 receptor antagonist SIB-1830 reduces l-DOPA-induced dyskinesia in the MPTP-lesioned primate model of Parkinson's disease. Abstr. Soc. Neurosci.

[241] Samadi P, Gregoire L, Morissette M, Calon F, Hadj T A, Dridi M, Belanger N, Meltzer L T, Bedard P J, Di P T (2008). mGluR5 metabotropic glutamate receptors and dyskinesias in MPTP monkeys. Neurobiol. Aging.

[242] Vernon A C, Palmer S, Datla K P, Zbarsky V, Croucher M J, Dexter D T (2005). Neuroprotective effects of metabotropic glutamate receptor ligands in a 6-hydroxydopamine rodent model of Parkinson's disease. Eur. J. Neurosci.

[243] Vernon A C, Zbarsky V, Datla K P, Croucher M J, Dexter D T (2007). Subtype selective antagonism of substantia nigra pars 
compacta Group I metabotropic glutamate receptors protects the nigrostriatal system against 6-hydroxydopamine toxicity *in vivo*. J. Neurochem.

[244] Blandini F, Nappi G, Greenamyre J T (2001). Subthalamic infusion of an NMDA antagonist prevents basal ganglia metabolic changes and nigral degeneration in a rodent model of Parkinson's disease. Ann. Neurol.

[245] Piallat B, Benazzouz A, Benabid A L (1996). Subthalamic nucleus lesion in rats prevents dopaminergic nigral neuron degeneration 
after striatal 6-OHDA injection: behavioural and immunohistochemical studies. Eur. J. Neurosci.

[246] Battaglia G, Busceti C L, Molinaro G, Biagioni F, Storto M, Fornai F, Nicoletti F, Bruno V (2004). Endogenous activation of mGlu5 metabotropic glutamate receptors contributes to the development 
of nigro-striatal damage induced by 1-Methyl-4-Phenyl-1,2,3,6-Tetrahydropyridine in mice. J. Neurosci.

[247] Samadi P, Rajput A, Calon F, Gregoire L, Hornykiewicz O, Rajput A H, Di P T (2009). Metabotropic glutamate receptor II in the brains of Parkinsonian patients. J. Neuropathol. Exp. Neurol.

[248] Dawson L, Chadha A, Megalou M, Duty S (2000). The group II metabotropic glutamate receptor agonist, DCG-IV, alleviates akinesia following intranigral or intraventricular administration in the reserpine-treated rat. Br. J. Pharmacol.

[249] Bradley S R, Marino M J, Wittmann M, Rouse S T, Awad H, Levey A I, Conn P J (2000). Activation of group II metabotropic glutamate receptors inhibits synaptic excitation of the substantia Nigra pars reticulata. J. Neurosci.

[250] Murray T K, Messenger M J, Ward M A, Woodhouse S, Osborne D J, Duty S, O'Neill M J (2002). Evaluation of the mGluR2/3 agonist LY379268 in rodent models of Parkinson's disease. Pharmacol. Biochem. Behav.

[251] Battaglia G, Busceti C L, Pontarelli F, Biagioni F, Fornai F, Paparelli A, Bruno V, Ruggieri S, Nicoletti F (2003). Protective role of group-II metabotropic glutamate receptors against nigro-striatal degeneration induced by 1-methyl-4-phenyl-1,2,3,6-tetrahydropyridine in mice. Neuropharmacology.

[252] Wang L, Kitai S T, Xiang Z (2005). Modulation of excitatory synaptic transmission by endogenous glutamate acting on presynaptic group II mGluRs in rat substantia nigra compacta. J. Neurosci. Res.

[253] Bradley S R, Standaert D G, Rhodes K J, Rees H D, Testa C M, Levey A I, Conn P J (1999). Immunohistochemical localization of subtype 4a metabotropic glutamate receptors in the rat and mouse basal ganglia. J. Comp. Neurol.

[254] Valenti O, Marino M J, Wittmann M, Lis E, DiLella A G, Kinney G G, Conn P J (2003). Group III metabotropic glutamate 
receptor-mediated modulation of the striatopallidal synapse. J. Neurosci.

[255] Lopez S, Turle-Lorenzo N, Acher F, De L E, Mele A, Amalric M (2007). Targeting group III metabotropic glutamate receptors produces complex behavioral effects in rodent models of Parkinson's disease. J. Neurosci.

[256] Marino M J, Williams D L, O'brien J A, Valenti O, McDonald T P, Clements M K, Wang R, DiLella A G, Hess J F, Kinney G G, Conn P J (2003). Allosteric modulation of group III metabotropic glutamate receptor 4: a potential approach to Parkinson's disease treatment. Proc. Natl. Acad Sci. USA.

[257] Beurrier C, Lopez S, Revy D, Selvam C, Goudet C, Lheron-del M, Gubellini P, Kerkerian-LeGoff L, Acher F, Pin J P, Amalric M (2009). Electrophysiological and behavioral evidence that modulation of metabotropic glutamate receptor 4 with a new agonist reverses experimental parkinsonism. FASEB J.

[258] Jiang Q, Yan Z, Feng J (2006). Activation of group III metabotropic glutamate receptors attenuates rotenone toxicity on dopaminergic neurons through a microtubule-dependent mechanism. J. Neurosci.

[259] Valenti O, Mannaioni G, Seabrook G R, Conn P J, Marino M J (2005). Group III metabotropic glutamate-receptor-mediated modulation of excitatory transmission in rodent substantia nigra pars compacta dopamine neurons. J. Pharmacol. Exp. Ther.

[260] Greco B, Lopez S, Van der Putten H P, Flor P J, Amalric M (2010). Metabotropic glutamate 7 receptor subtype modulates motor symptoms in rodent models of Parkinson's disease. J. Pharmacol. Exper. Therap.

[261] Hagerman R J, Hagerman P J (2002). The fragile X premutation: into the phenotypic fold. Curr. Opin. Genet Dev.

[262] Crawford D C, Acuna J M, Sherman S L (2001). FMR1 and the fragile X syndrome: human genome epidemiology review. Genet Med.

[263] Schütt J, Falley K, Richter D, Kreienkamp H J, Kindler S (2009). Fragile X mental retardation protein regulates the levels of scaffold proteins and glutamate receptors in postsynaptic densities. J. Biol. Chem.

[264] Zukin R S, Richter J D, Bagni C (2009). Signals synapses and synthesis: how new proteins control plasticity. Front. Neural. Circuits.

[265] (1994). The Dutch-Belgian Fragile X Consortium Fmr1 knockout mice: a model to study fragile X mental retardation. The Dutch-Belgian Fragile X Consortium. Cell.

[266] Kooy R F, D'Hooge R, Reyniers E, Bakker C E, Nagels G, De B K, Storm K, Clincke G, De Deyn P P, Oostra B A, Willems P J (1996). Transgenic mouse model for the fragile X syndrome. Am. J. Med. Genet.

[267] Yan Q J, Rammal M, Tranfaglia M, Bauchwitz R P (2005). Suppression of two major Fragile X Syndrome mouse model phenotypes by the mGluR5 antagonist MPEP. Neuropharmacology.

[268] Qin M, Kang J, Smith C B (2005). A null mutation for Fmr1 in female mice: effects on regional cerebral metabolic rate for glucose and relationship to behavior. Neuroscience.

[269] Qin M, Kang J, Smith C B (2002). Increased rates of cerebral glucose metabolism in a mouse model of fragile X mental retardation. Proc. Natl. Acad Sci. USA.

[270] Mineur Y S, Huynh L X, Crusio W E (2006). Social behavior deficits in the Fmr1 mutant mouse. Behav. Brain Res.

[271] Spencer C M, Alekseyenko O, Serysheva E, Yuva-Paylor L A, Paylor R (2005). Altered anxiety-related and social behaviors in the Fmr1 knockout mouse model of fragile X syndrome. Genes Brain Behav.

[272] Irwin S A, Patel B, Idupulapati M, Harris J B, Crisostomo R A, Larsen B P, Kooy F, Willems P J, Cras P, Kozlowski P B, Swain R A, Weiler I J, Greenough W T (2001). Abnormal 
dendritic spine characteristics in the temporal and visual cortices of patients with fragile-X syndrome: a quantitative examination. Am. J. Med. Genet.

[273] Hinton V J, Brown W T, Wisniewski K, Rudelli R D (1991). Analysis of neocortex in three males with the fragile X syndrome. Am. J. Med. Genet.

[274] Rudelli R D, Brown W T, Wisniewski K, Jenkins E C, Laure-Kamionowska M, Connell F, Wisniewski H M (1985). Adult fragile X syndrome. Clinico-neuropathologic findings. Acta Neuropathol.

[275] Grossman A W, Elisseou N M, McKinney B C, Greenough W T (2006). Hippocampal pyramidal cells in adult Fmr1 knockout mice exhibit an immature-appearing profile of dendritic spines. Brain Res.

[276] Galvez R, Greenough W T (2005). Sequence of abnormal dendritic spine development in primary somatosensory cortex of a mouse model of the fragile X mental retardation syndrome. Am. J. Med. Genet A.

[277] Comery T A, Harris J B, Willems P J, Oostra B A, Irwin S A, Weiler I J, Greenough W T (1997). Abnormal dendritic spines in fragile X knockout mice: maturation and pruning deficits. Proc. Natl. Acad Sci. USA.

[278] Bagni C, Greenough W T (2005). From mRNP trafficking to spine dysmorphogenesis: the roots of fragile X syndrome. Nat. Rev. Neurosci.

[279] Vaillend C, Poirier R, Laroche S (2008). Genes plasticity and mental retardation. Behav. Brain Res.

[280] Bear M F, Huber K M, Warren S T (2004). The mGluR theory of fragile X mental retardation. Trends Neurosci.

[281] Huber K M, Gallagher S M, Warren S T, Bear M F (2002). Altered synaptic plasticity in a mouse model of fragile X mental retardation. Proc. Natl. Acad Sci. USA.

[282] Sharma A, Hoeffer C A, Takayasu Y, Miyawaki T, McBride S M, Klann E, Zukin R S (2010). Dysregulation of mTOR signaling in fragile X syndrome. J. Neurosci.

[283] Waung M W, Huber K M (2009). Protein translation in synaptic plasticity: mGluR-LTD, Fragile X. Curr. Opin. Neurobiol.

[284] Nosyreva E D, Huber K M (2006). Metabotropic receptor-dependent long-term depression persists in the absence of protein synthesis in the mouse model of fragile X syndrome. J. Neurophysiol.

[285] Dölen G, Osterweil E, Rao B S, Smith G B, Auerbach B D, Chattarji S, Bear M F (2007). Correction of fragile X syndrome in mice. Neuron.

[286] Yuskaitis C J, Mines M A, King M K, Sweatt J D, Miller C A, Jope R S (2010). Lithium ameliorates altered glycogen synthase kinase-3 and behavior in a mouse model of fragile X syndrome. Biochem. Pharmacol.

[287] McBride S M, Choi C H, Wang Y, Liebelt D, Braunstein E, Ferreiro D, Sehgal A, Siwicki K K, Dockendorff T C, Nguyen H T, McDonald T V, Jongens T A (2005). Pharmacological rescue of synaptic plasticity, courtship behavior and mushroom body defects in a Drosophila model of fragile X syndrome. Neuron.

[288] Choi C H, McBride S M, Schoenfeld B P, Liebelt D A, Ferreiro D, Ferrick N J, Hinchey P, Kollaros M, Rudominer R L, Terlizzi A M, Koenigsberg E, Wang Y, Sumida A, Nguyen H T, Bell A J, McDonald T V, Jongens T A (2010). Age-dependent cognitive impairment in a Drosophila Fragile X model and its pharmacological rescue. Biogerontology.

[289] Tucker B, Richards R I, Lardelli M (2006). Contribution of mGluR and Fmr1 functional pathways to neurite morphogenesis craniofacial development and fragile X syndrome. Hum. Mol. Genet.

[290] de Vrij F M, Levenga J, van der Linde H C, Koekkoek S K, De Zeeuw C I, Nelson D L, Oostra B A, Willemsen R (2008). Rescue of behavioral phenotype and neuronal protrusion morphology in Fmr1 KO mice. Neurobiol. Dis.

[291] Berry-Kravis E, Hessl D, Coffey S, Hervey C, Schneider A, Yuhas J, Hutchison J, Snape M, Tranfaglia M, Nguyen D V, Hagerman R (2009). A pilot open label, single dose trial of fenobam 
in adults with fragile X syndrome. J. Med. Genet.

[292] Jacob W, Gravius A, Pietraszek M, Nagel J, Belozertseva I, Shekunova E, Malyshkin A, Greco S, Barberi C, Danysz W (2009). The anxiolytic and analgesic properties of fenobam, a potent mGlu5 receptor antagonist in relation to the impairment of 
learning. Neuropharmacology.

[293] Gravius A, Pietraszek M, Schafer D, Schmidt W J, Danysz W (2005). Effects of mGlu1 and mGlu5 receptor antagonists on negatively reinforced learning. Behav. Pharmacol.

[294] Bikbaev A, Neyman S, Ngomba R T, Conn P J, Nicoletti F, Manahan-Vaughan D (2008). MGluR5 mediates the interaction between late-LTP network activity and learning. PLoS One.

[295] Hagerman R J, Ono M Y, Hagerman P J (2005). Recent advances in fragile X: a model for autism and neurodegeneration. Curr. Opin. Psychiatry.

[296] Silverman J L, Tolu S S, Barkan C L, Crawley J N (2010). Repetitive Self-Grooming Behavior in the BTBR Mouse Model of Autism is Blocked by the mGluR5 Antagonist MPEP. Neuropsychopharmacology.

[297] Rego A C, de Almeida L P (2005). Molecular targets and therapeutic strategies in Huntington's disease. Curr. Drug Targets CNS Neurol. Disord.

[298] Li S H, Li X J (2004). Huntingtin-protein interactions and the pathogenesis of Huntington's disease. Trends Genet.

[299] Vonsattel J P, DiFiglia M (1998). Huntington disease. J. Neuropathol. Exp. Neurol.

[300] Taylor-Robinson S D, Weeks R A, Bryant D J, Sargentoni J, Marcus C D, Harding A E, Brooks D J (1996). Proton magnetic resonance spectroscopy in Huntington's disease: evidence in favour of the glutamate excitotoxic theory. Mov. Disord.

[301] Fan M M, Raymond L A (2007). N-methyl-D-aspartate (NMDA) 
receptor function and excitotoxicity in Huntington's disease. Prog. Neurobiol.

[302] Bruno V, Ksiazek I, Battaglia G, Lukic S, Leonhardt T, Sauer D, Gasparini F, Kuhn R, Nicoletti F, Flor P J (2000). Selective blockade of metabotropic glutamate receptor subtype 5 is neuroprotective. Neuropharmacology.

[303] Anborgh P H, Godin C, Pampillo M, Dhami G K, Dale L B, Cregan S P, Truant R, Ferguson S S (2005). Inhibition of metabotropic glutamate receptor signaling by the huntingtin-binding protein optineurin. J. Biol. Chem.

[304] Ribeiro F M, Paquet M, Ferreira L T, Cregan T, Swan P, Cregan S P, Ferguson S S (2010). Metabotropic glutamate receptor-mediated cell signaling pathways are altered in a mouse model of Huntington's disease. J. Neurosci.

[305] Orlando L R, Standaert D G, Penney J B, Young A B (1995). Metabotropic receptors in excitotoxicity: (S)-4-carboxy-3-hydroxyphenylglycine ((S)-4C3HPG) protects against rat striatal quinolinic acid lesions. Neurosci. Lett.

[306] Li J Y, Popovic N, Brundin P (2005). The use of the R6 transgenic mouse models of Huntington's disease in attempts to develop novel therapeutic strategies. Neuro. Rx.

[307] Cha J H, Kosinski C M, Kerner J A, Alsdorf S A, Man-giarini L, Davies S W, Penney J B, Bates G P, Young A B (1998). Altered brain neurotransmitter receptors in transgenic mice expressing a portion of an abnormal human huntington disease gene. Proc. Natl. Acad Sci. USA.

[308] Behrens P F, Franz P, Woodman B, Lindenberg K S, Landwehrmeyer G B (2002). Impaired glutamate transport and glutamate-glutamine cycling: downstream effects of the Huntington mutation. Brain.

[309] Schiefer J, Sprunken A, Puls C, Luesse H G, Milkereit A, Milkereit E, Johann V, Kosinski C M (2004). The metabotropic glutamate receptor 5 antagonist MPEP and the mGluR2 agonist LY379268 modify disease progression in a transgenic mouse model of Huntington's disease. Brain Res.

[310] Schiefer J, Landwehrmeyer G B, Luesse H G, Sprunken A, Puls C, Milkereit A, Milkereit E, Kosinski C M (2002). Riluzole prolongs survival time and alters nuclear inclusion formation in a transgenic mouse model of Huntington's disease. Mov. Disord.

[311] Landwehrmeyer G B, Dubois B, de Yebenes J G, Kremer B, Gaus W, Kraus P H, Przuntek H, Dib M, Doble A, Fischer W, Ludolph A C (2007). Riluzole in Huntington's disease: a 3-year, randomized controlled study. Ann. Neurol.

[312] Hardy J, Allsop D (1991). Amyloid deposition as the central event in the aetiology of Alzheimer's disease. Trends Pharmacol. Sci.

[313] Francis P T (2003). Glutamatergic systems in Alzheimer's disease. Int. J. Geriatr. Psychiatry.

[314] Bruno V, Battaglia G, Copani A, D'Onofrio M, Di I P, De B A, Melchiorri D, Flor P J, Nicoletti F (2001). Metabotropic glutamate receptor subtypes as targets for neuroprotective drugs. J. Cereb. Blood Flow. Metab.

[315] Albasanz J L, Dalfo E, Ferrer I, Martin M (2005). Impaired metabotropic glutamate receptor/phospholipase C signaling pathway in the cerebral cortex in Alzheimer's disease and dementia with Lewy bodies correlates with stage of Alzheimer's-disease-related changes. Neurobiol. Dis.

[316] Lee R K, Wurtman R J, Cox A J, Nitsch R M (1995). Amyloid precursor protein processing is stimulated by metabotropic glutamate receptors. Proc. Natl. Acad Sci. USA.

[317] Jolly-Tornetta C, Gao Z Y, Lee V M, Wolf B A (1998). Regulation of amyloid precursor protein secretion by glutamate receptors in human Ntera 2 neurons. J. Biol. Chem.

[318] Bruno V, Battaglia G, Ksiazek I, van der P H, Catania M V, Giuffrida R, Lukic S, Leonhardt T, Inderbitzin W, Gasparini F, Kuhn R, Hampson D R, Nicoletti F, Flor P J (2000). Selective activation of mGlu4 metabotropic glutamate receptors is protective against excitotoxic neuronal death. J. Neurosci.

[319] Lee H G, Zhu X, Casadesus G, Pallas M, Camins A, O'Neill M J, Nakanishi S, Perry G, Smith M A (2009). The effect of mGluR2 activation on signal transduction pathways and neuronal cell survival. Brain Res.

[320] Bond A, Jones N M, Hicks C A, Whiffin G M, Ward M A, O'Neill M F, Kingston A E, Monn J A, Ornstein P L, Schoepp D D, Lodge D, O'Neill M J (2000). Neuroprotective effects of LY379268 a selective mGlu2/3 receptor agonist: investigations into possible mechanism of action *in vivo*. J. Pharmacol. Exp. Ther.

[321] Lee H G, Ogawa O, Zhu X, O'Neill M J, Petersen R B, Castellani R J, Ghanbari H, Perry G, Smith M A (2004). Aberrant expression of metabotropic glutamate receptor 2 in the vulnerable neurons of Alzheimer's disease. Acta Neuropathol.

[322] Neugebauer V, Lucke T, Schaible H G (1994). Requirement of metabotropic glutamate receptors for the generation of inflammation-evoked hyperexcitability in rat spinal cord neurons. Eur. J. Neurosci.

[323] Goudet C, Magnaghi V, Landry M, Nagy F, Gereau R W, Pin J P (2009). Metabotropic receptors for glutamate and GABA in pain. Brain Res. Rev.

[324] Lourenco N F, Schadrack J, Berthele A, Zieglgansberger W, Tolle T R, Castro-Lopes J M (2000). Differential distribution of metabotropic glutamate receptor subtype mRNAs in the thalamus of the rat. Brain Res.

[325] Petralia R S, Wang Y X, Singh S, Wu C, Shi L, Wei J, Wenthold R J (1997). A monoclonal antibody shows discrete cellular and subcellular localizations of mGluR1 alpha metabotropic glutamate receptors. J. Chem. Neuroanat.

[326] Neugebauer V, Li W, Bird G C, Han J S (2004). The amygdala and persistent pain. Neuroscientist.

[327] Neugebauer V (2007). The amygdala: different pains, different mechanisms. Pain.

[328] Saab C Y, Wang J, Gu C, Garner K N, Al-Chaer E D (2007). Microglia: a newly discovered role in visceral hypersensitivity Neuron. Glia. Biol.

[329] Dolan S, Nolan A M (2000). Behavioural evidence supporting a differential role for group I and II metabotropic glutamate receptors in spinal nociceptive transmission. Neuropharmacology.

[330] Fisher K, Coderre T J (1996). The contribution of metabotropic glutamate receptors (mGluRs) to formalin-induced nociception. Pain.

[331] Fisher K, Fundytus M E, Cahill C M, Coderre T J (1998). Intrathecal administration of the mGluR compound, (S)-4CPG, attenuates hyperalgesia and allodynia associated with sciatic nerve constriction injury in rats. Pain.

[332] Lorrain D S, Correa L, Anderson J, Varney M (2002). Activation of spinal group I metabotropic glutamate receptors in rats evokes local glutamate release and spontaneous nociceptive behaviors: effects of 2-methyl-6-(phenylethynyl)-pyridine pretreatment. Neurosci. Lett.

[333] Fundytus M E, Fisher K, Dray A, Henry J L, Coderre T J (1998). *In vivo* antinociceptive activity of anti-rat mGluR1 and mGluR5 antibodies in rats. Neuroreport.

[334] Fundytus M E, Yashpal K, Chabot J G, Osborne M G, Lefebvre C D, Dray A, Henry J L, Coderre T J (2001). Knockdown of spinal metabotropic glutamate receptor 1 (mGluR(1)) alleviates pain and restores opioid efficacy after nerve injury in rats. Br. J. Pharmacol.

[335] Fundytus M E, Osborne M G, Henry J L, Coderre T J, Dray A (2002). Antisense oligonucleotide knockdown of mGluR1 alleviates hyperalgesia and allodynia associated with chronic inflammation. Pharmacol. Biochem. Behav.

[336] Noda K, Anzai T, Ogata M, Akita H, Ogura T, Saji M (2003). Antisense knockdown of spinal-mGluR1 reduces the sustained phase of formalin-induced nociceptive responses. Brain Res.

[337] Young M R, Blackburn-Munro G, Dickinson T, Johnson M J, Anderson H, Nakalembe I, Fleetwood-Walker S M (1998). Antisense ablation of type I metabotropic glutamate receptor mGluR1 inhibits spinal nociceptive transmission. J. Neurosci.

[338] Adwanikar H, Karim F, Gereau R W (2004). Inflammation persistently enhances nocifensive behaviors mediated by spinal group I mGluRs through sustained ERK activation. Pain.

[339] Salt T E, Turner J P (1998). Reduction of sensory and metabotropic glutamate receptor responses in the thalamus by the novel metabotropic glutamate receptor-1-selective antagonist S-2-methyl-4-carboxy-phenylglycine. Neuroscience.

[340] Han J S, Neugebauer V (2005). mGluR1 and mGluR5 antagonists in the amygdala inhibit different components of audible and ultrasonic vocalizations in a model of arthritic pain. Pain.

[341] Kingston A E, Griffey K, Johnson M P, Chamberlain M J, Kelly G, Tomlinson R, Wright R A, Johnson B G, Schoepp D D, Harris J R, Clark B P, Baker R S, Tizzano J T (2002). Inhibition of group I metabotropic glutamate receptor responses *in vivo* in rats by a new generation of carboxyphenylglycine-like amino acid antagonists. Neurosci. Lett.

[342] Lavreysen H, Janssen C, Bischoff F, Langlois X, Leysen J E, Lesage A S (2003). [3H]R214127: a novel high-affinity radioligand for the mGlu1 receptor reveals a common binding site shared by multiple allosteric antagonists. Mol. Pharmacol.

[343] Varty G B, Grilli M, Forlani A, Fredduzzi S, Grzelak M E, Guthrie D H, Hodgson R A, Lu S X, Nicolussi E, Pond A J, Parker E M, Hunter J C, Higgins G A, Reggiani A, Bertorelli R (2005). The antinociceptive and anxiolytic-like effects of the metabotropic glutamate receptor 5 (mGluR5) antagonists MPEP and MTEP and the mGluR1 antagonist LY456236 in rodents: a comparison of efficacy and side-effect profiles. Psychopharmacology (Berl).

[344] Zhang L, Lu Y, Chen Y, Westlund K N (2002). Group I metabotropic glutamate receptor antagonists block secondary thermal hyperalgesia in rats with knee joint inflammation. J. Pharmacol. Exp. Ther.

[345] Salt T E, Turner J P, Kingston A E (1999). Evaluation of agonists and antagonists acting at Group I metabotropic glutamate receptors in the thalamus *in vivo*. Neuropharmacology.

[346] Lee K S, Kim J, Yoon Y W, Lee M G, Hong S K, Han H C (2007). The peripheral role of group I metabotropic glutamate receptors on nociceptive behaviors in rats with knee joint inflammation. Neurosci. Lett.

[347] El-Kouhen O, Lehto S G, Pan J B, Chang R, Baker S J, Zhong C, Hollingsworth P R, Mikusa J P, Cronin E A, Chu K L, McGaraughty S P, Uchic M E, Miller L N, Rodell N M, Patel M, Bhatia P, Mezler M, Kolasa T, Zheng G Z, Fox G B, Stewart A O, Decker M W, Moreland R B, Brioni J D, Honore P (2006). Blockade of mGluR1 receptor results in analgesia and disruption of motor and cognitive performances: effects of A-841720 a novel non-competitive mGluR1 receptor antagonist. Br. J. Pharmacol.

[348] Zheng G Z, Bhatia P, Daanen J, Kolasa T, Patel M, Latshaw S, El Kouhen O F, Chang R, Uchic M E, Miller L, Nakane M, Lehto S G, Honore M P, Moreland R B, Brioni J D, Stewart A O (2005). Structure-activity relationship of triazafluorenone derivatives as potent and selective mGluR1 antagonists. J. Med. Chem.

[349] Zhu C Z, Baker S, Ei-Kouhen O, Lehto S G, Hollingsworth P R, Gauvin D M, Hernandez G, Zheng G, Chang R, Moreland R B, Stewart A O, Brioni J D, Honore P (2008). Analgesic 
activity of metabotropic glutamate receptor 1 antagonists on 
spontaneous post-operative pain in rats. Eur. J. Pharmacol.

[350] Binns K E, Salt T E (2001). Actions of the systemically active metabotropic glutamate antagonist MPEP on sensory responses of thalamic neurones. Neuropharmacology.

[351] Dogrul A, Ossipov M H, Lai J, Malan T P, Porreca F (2000). Peripheral and spinal antihyperalgesic activity of SIB-1757
metabotropic glutamate receptor (mGLUR(5)) antagonist in 
experimental neuropathic pain in rats. Neurosci. Lett.

[352] Zhu C Z, Wilson S G, Mikusa J P, Wismer C T, Gauvin D M, Lynch J J, Wade C L, Decker M W, Honore P (2004). Assessing the role of metabotropic glutamate receptor 5 in multiple nociceptive modalities. Eur. J. Pharmacol.

[353] Palazzo E, Marabese I, de N V, Oliva P, Rossi F, Berrino L, Rossi F, Maione S (2001). Metabotropic and NMDA glutamate receptors participate in the cannabinoid-induced antinociception. Neuropharmacology.

[354] Wroblewska B (2006). NAAG as a neurotransmitter. Adv. Exp. Med. Biol.

[355] Nagel J, Belozertseva I, Greco S, Kashkin V, Malyshkin A, Jirgensons A, Shekunova E, Eilbacher B, Bespalov A, Danysz W (2006). Effects of NAAG peptidase inhibitor 2-PMPA in model chronic pain - relation to brain concentration. Neuropharmacology.

[356] Yamamoto T, Hirasawa S, Wroblewska B, Grajkowska E, Zhou J, Kozikowski A, Wroblewski J, Neale J H (2004). Antinociceptive effects of N-acetylaspartylglutamate (NAAG) peptidase 
inhibitors ZJ-11, ZJ-17 and ZJ-43 in the rat formalin test and in the rat neuropathic pain model. Eur. J. Neurosci.

[357] Dolan S, Nolan A M (2002). Behavioral evidence supporting a differential role for spinal group I and II metabotropic glutamate receptors in inflammatory hyperalgesia in sheep. Neuropharmacology.

[358] Li W, Neugebauer V (2006). Differential changes of group II and group III mGluR function in central amygdala neurons in a model of arthritic pain. J. Neurophysiol.

[359] Neugebauer V, Chen P S, Willis W D (2000). Groups II and III metabotropic glutamate receptors differentially modulate brief and prolonged nociception in primate STT cells. J. Neurophysiol.

[360] Sharpe E F, Kingston A E, Lodge D, Monn J A, Headley P M (2002). Systemic pre-treatment with a group II mGlu agonist, LY379268, reduces hyperalgesia *in vivo*. Br. J. Pharmacol.

[361] Simmons R M, Webster A A, Kalra A B, Iyengar S (2002). Group II mGluR receptor agonists are effective in persistent and neuropathic pain models in rats. Pharmacol. Biochem. Behav.

[362] Varney M A, Gereau R W (2002). Metabotropic glutamate receptor involvement in models of acute and persistent pain: prospects for the development of novel analgesics. Curr. Drug Targets CNS Neurol. Disord.

[363] Yang D, Gereau R W (2003). Peripheral group II metabotropic glutamate receptors mediate endogenous antiallodynia in inflammation. Pain.

[364] Neugebauer V, Carlton S M (2002). Peripheral metabotropic glutamate receptors as drug targets for pain relief. Expert Opin. Ther. 
Targets.

[365] Jones C K, Eberle E L, Peters S C, Monn J A, Shannon H E (2005). Analgesic effects of the selective group II (mGlu2/3) metabotropic glutamate receptor agonists LY379268 and LY389795 in persistent and inflammatory pain models after acute and repeated dosing. Neuropharmacology.

[366] Chen S R, Pan H L (2005). Distinct roles of group III metabotropic glutamate receptors in control of nociception and dorsal horn 
neurons in normal and nerve-injured Rats. J. Pharmacol. Exp. Ther.

[367] Goudet C, Chapuy E, Alloui A, Acher F, Pin J P, Eschalier A (2008). Group III metabotropic glutamate receptors inhibit hyperalgesia in animal models of inflammation and neuropathic pain. Pain.

[368] Han J S, Bird G C, Neugebauer V (2004). Enhanced group III mGluR-mediated inhibition of pain-related synaptic plasticity in the amygdala. Neuropharmacology.

[369] Palazzo E, Fu Y, Ji G, Maione S, Neugebauer V (2008). Group III mGluR7 and mGluR8 in the amygdala differentially modulate nocifensive and affective pain behaviors. Neuropharmacology.

[370] Bradford H F (1995). Glutamate GABA and epilepsy. Prog. Neurobiol.

[371] Chapman A G, Elwes R D, Millan M H, Polkey C E,  
Meldrum B S (1996). Role of glutamate and aspartate in epileptogenesis contribution of microdialysis studies in animal and man. Epilepsy Res. Suppl.

[372] Chapman A G (2000). Glutamate and epilepsy. J. Nutr.

[373] Chapman A, Meldrum B (1991). Excitatory amino acids in epilepsy and novel anti-epileptic drugs. Epilepsy Res. Suppl.

[374] Meldrum B S (1991). Excitatory amino acid transmitters in epilepsy. Epilepsia.

[375] Meldrum B S (1992). Excitatory amino acids in epilepsy and potential novel therapies. Epilepsy Res.

[376] Notenboom R G, Hampson D R, Jansen G H, van Rijen P C, van Veelen C W, van N O, de Graan P N (2006). Up-regulation of hippocampal metabotropic glutamate receptor 5 in temporal lobe epilepsy patients. Brain.

[377] Aronica E, Gorter J A, Jansen G H, van Veelen C W, van Rijen P C, Ramkema M, Troost D (2003). Expression and cell distribution of group I and group II metabotropic glutamate receptor subtypes in taylor-type focal cortical dysplasia. Epilepsia.

[378] Chapman A G (1998). Glutamate receptors in epilepsy. Prog. Brain Res.

[379] Moldrich R X, Beart P M (2003). Emerging signalling and protein interactions mediated *via* metabotropic glutamate receptors. Curr. Drug Targets CNS Neurol. Disord.

[380] Tang F R (2005). Agonists and antagonists of metabotropic glutamate receptors: anticonvulsants and antiepileptogenic agents?. Curr Neuropharmacol.

[381] Tizzano J P, Griffey K I, Johnson J A, Fix A S, Helton D R, Schoepp D D (1993). Intracerebral 1S,3R-1-aminocyclopentane-1,3-dicarboxylic acid (1S,3R-ACPD) produces limbic seizures that are not blocked by ionotropic glutamate receptor antagonists. Neurosci. Lett.

[382] Thomsen C, Klitgaard H, Sheardown M, Jackson H C, Eskesen K, Jacobsen P, Treppendahl S, Suzdak P D (1994). (S)-4-carboxy-3-hydroxyphenylglycine an antagonist of metabotropic glutamate receptor (mGluR) 1a and an agonist of mGluR2 protects against audiogenic seizures in DBA/2 mice. J. Neurochem.

[383] Dalby N O, Thomsen C (1996). Modulation of seizure activity in mice by metabotropic glutamate receptor ligands. J. Pharmacol. Exp. Ther.

[384] Chapman A G, Yip P K, Yap J S, Quinn L P, Tang  
E, Harris J R, Meldrum B S (1999). Anticonvulsant actions of LY 367385 ((+)-2-methyl-4-carboxyphenylglycine) and AIDA ((RS)-1-aminoindan-1,5-dicarboxylic acid). Eur. J. Pharmacol.

[385] Burgess D L, Jones J M, Meisler M H, Noebels J L (1997). Mutation of the Ca^2+^ channel beta subunit gene Cchb4 is associated with ataxia and seizures in the lethargic (lh) mouse. Cell.

[386] Nagaraja R Y, Grecksch G, Reymann K G, Schroeder H, Becker A (2004). Group I metabotropic glutamate receptors interfere in different ways with pentylenetetrazole seizures, kindling and kindling-related learning deficits. Naunyn. Schmiedebergs. Arch. Pharmacol.

[387] Thomsen C, Dalby N O (1998). Roles of metabotropic glutamate receptor subtypes in modulation of pentylenetetrazole-induced seizure activity in mice. Neuropharmacology.

[388] Smolders I, Lindekens H, Clinckers R, Meurs A, O'Neill M J, Lodge D, Ebinger G, Michotte Y (2004). *In vivo* modulation of extracellular hippocampal glutamate and GABA levels and limbic seizures by group I and II metabotropic glutamate receptor ligands. J. Neurochem.

[389] Renaud J, Emond M, Meilleur S, Psarropoulou C, Carmant L (2002). AIDA a class I metabotropic glutamate-receptor antagonist limits kainate-induced hippocampal dysfunction. Epilepsia.

[390] Chapman A G, Nanan K, Williams M, Meldrum B S (2000). Anticonvulsant activity of two metabotropic glutamate group I antagonists selective for the mGlu5 receptor: 2-methyl-6-(phenylethynyl)-pyridine (MPEP): and (E)-6-methyl-2-styryl-pyridine (SIB 1893). Neuropharmacology.

[391] Ermolinsky B, Pacheco Otalora L F, Arshadmansab M F, Zarei M M, Garrido-Sanabria E R (2008). Differential changes in mGlu2 and mGlu3 gene expression following pilocarpine-induced status epilepticus: a comparative real-time PCR analysis. Brain Res.

[392] Garrido-Sanabria E R, Otalora L F, Arshadmansab M F, Herrera B, Francisco S, Ermolinsky B S (2008). Impaired expression and function of group II metabotropic glutamate receptors in pilocarpine-treated chronically epileptic rats. Brain Res.

[393] Attwell P J, Singh K N, Jane D E, Croucher M J, Bradford H F (1998). Anticonvulsant and glutamate release-inhibiting properties of the highly potent metabotropic glutamate receptor agonist (2S,2'R, 3'R)-2-(2',3'-dicarboxycyclopropyl)glycine (DCG-IV). Brain Res.

[394] Tang E, Yip P K, Chapman A G, Jane D E, Meldrum B S (1997). Prolonged anticonvulsant action of glutamate metabotropic receptor agonists in inferior colliculus of genetically epilepsy-prone rats. Eur. J. Pharmacol.

[395] Miyamoto M, Ishida M, Shinozaki H (1997). Anticonvulsive and neuroprotective actions of a potent agonist (DCG-IV) for group II metabotropic glutamate receptors against intraventricular kainate in the rat. Neuroscience.

[396] Moldrich R X, Jeffrey M, Talebi A, Beart P M, Chapman A G, Meldrum B S (2001). Anti-epileptic activity of group II metabotropic
glutamate receptor agonists (--)-2-oxa-4-aminobicyclo[3.1.0]
hexane-4,6-dicarboxylate (LY379268) and (--)-2-thia-4-aminobicyclo
[3.1.0]hexane-4,6-dicarboxylate (LY389795). Neuropharmacology.

[397] Duvoisin R M, Zhang C, Pfankuch T F, O'Connor H, Gayet-Primo J, Quraishi S, Raber J (2005). Increased measures of anxiety and weight gain in mice lacking the group III metabotropic glutamate receptor mGluR8. Eur. J. Neurosci.

[398] Sansig G, Bushell T J, Clarke V R, Rozov A, Burnashev N, Portet C, Gasparini F, Schmutz M, Klebs K, Shigemoto R, Flor P J, Kuhn R, Knoepfel T, Schroeder M, Hampson D R, Collett V J, Zhang C, Duvoisin R M, Collingridge G L,  
van der P H (2001). Increased seizure susceptibility in mice lacking 
metabotropic glutamate receptor 7. J. Neurosci.

[399] Kral T, Erdmann E, Sochivko D, Clusmann H, Schramm J, Dietrich D (2003). Down-regulation of mGluR8 in pilocarpine epileptic rats. Synapse.

[400] Chen J, Larionov S, Pitsch J, Hoerold N, Ullmann C,  
Elger C E, Schramm J, Becker A J ( 2005). Expression analysis of 
metabotropic glutamate receptors I and III in mouse strains with different susceptibility to experimental temporal lobe epilepsy. Neurosci. Let.

[401] Ghauri M, Chapman A G, Meldrum B S (1996). Convulsant and anticonvulsant actions of agonists and antagonists of group III mGluRs. Neuroreport.

[402] Chapman A G, Talebi A, Yip P K, Meldrum B S (2001). Anticonvulsant activity of a mGlu(4alpha) receptor selective agonist, (1S,3R,4S)-1-aminocyclopentane-1 2 4-tricarboxylic acid. Eur. J. Pharmacol.

[403] Chapman A G, Nanan K, Yip P, Meldrum B S (1999). Anticonvulsant activity of a metabotropic glutamate receptor 8 preferential agonist (R,S)-4-phosphonophenylglycine. Eur. J. Pharmacol.

[404] Gasparini F, Bruno V, Battaglia G, Lukic S, Leonhardt T, Inderbitzin W, Laurie D, Sommer B, Varney M A, Hess S D, Johnson E C, Kuhn R, Urwyler S, Sauer D, Portet C, Schmutz M, Nicoletti F, Flor P J (1999). (R,S)-4-phosphonophenyl-
glycine a potent and selective group III metabotropic glutamate receptor agonist is anticonvulsive and neuroprotective *in vivo*. J. Pharmacol. Exp. Ther.

[405] Folbergrova J, Druga R, Haugvicova R, Mares P, Otahal J (2008). Anticonvulsant and neuroprotective effect of (S)-3,4-dicarboxyphenylglycine against seizures induced in immature rats by 
homocysteic acid. Neuropharmacology.

[406] Hosford D A, Wang Y (1997). Utility of the lethargic (lh/lh) mouse model of absence seizures in predicting the effects of lamotrigine vigabatrin tiagabine gabapentin and topiramate against human absence seizures. Epilepsia.

